# Supplementary material for: Scalable psychological interventions for Syrian refugees in Europe and the Middle East: STRENGTHS study protocol for a prospective individual participant data meta-analysis
Source: BMJ Open. 2022 Apr 20;12(4):e058101. doi: 10.1136/bmjopen-2021-058101 (PMC9021771; doi:10.1136/bmjopen-2021-058101)
Supplement: Author's manuscript [file bmjopen-2021-058101.draft_revisions.pdf]

BMJ Open is committed to open peer review. As part of this commitment we make the peer review history of every article we publish publicly available.

When an article is published we post the peer reviewers' comments and the authors' responses online. We also post the versions of the paper that were used during peer review. These are the versions that the peer review comments apply to.

The versions of the paper that follow are the versions that were submitted during the peer review process. They are not the versions of record or the final published versions. They should not be cited or distributed as the published version of this manuscript.

BMJ Open is an open access journal and the full, final, typeset and author-corrected version of record of the manuscript is available on our site with no access controls, subscription charges or pay-per-view fees (http://bmjopen.bmj.com).

If you have any questions on BMJ Open's open peer review process please email info.bmjopen@bmj.com

BMJ Open

# Scalable Psychological Interventions for Syrian Refugees in Europe and the Middle East: STRENGTHS Study Protocol for a Prospective Individual Participant Data Meta-Analysis



| | |
|---|---|
| Journal: | *BMJ Open* |
| Manuscript ID | bmjopen-2021-058101 |
| Article Type: | Protocol |
| Date Submitted by the Author: | 06-Oct-2021 |





| | |
|---|---|
| Complete List of Authors: | de Graaff, Anne; Vrije Universiteit Amsterdam, Department of Clinical, Neuro- and Developmental Psychology and World Health Organization Collaborating Center for Research and Dissemination of Psychological Interventions<br>Cuijpers, Pim; Vrije Universiteit Amsterdam, Department of Clinical, Neuro- and Developmental Psychology and World Health Organization Collaborating Center for Research and Dissemination of Psychological Interventions<br>Acarturk, Ceren ; Koç University,<br>Akhtar, Aemal; University of New South Wales<br>Alkneme, Mhd; Freie Universitat Berlin<br>Aoun, May; War Child<br>Awwad, Manar; International Medical Corps<br>Bawaneh, Ahmad; International Medical Corps<br>Brown, Felicity; War Child; University of Amsterdam<br>Bryant, Richard ; University of New South Wales, Sydney, Australia,<br>Burchert, Sebastian; Freie Universität Berlin Fachbereich Erziehungswissenschaft und Psychologie, Clinical-Psychological Intervention<br>Carswell, Kenneth; World Health Organization, Department of Mental Health & Substance Abuse<br>Drogendijk, Annelieke; ARQ National Psychotrauma Centre<br>Engels, Michelle; International Federation of Red Cross and Red Crescent Societies Reference Centre for Psychosocial Support<br>Fuhr, Daniela; London School of Hygiene and Tropical Medicine,<br>Hansen, Pernille; International Federation of Red Cross and Red Crescent Societies Reference Centre for Psychosocial Support<br>van 't Hof, Edith; World Health Organization<br>Giardinelli, Luana; International Medical Corps<br>Hemmo, Mahmoud; University Hospital Zurich<br>Hessling, Jonas; Freie Universität Berlin Fachbereich Erziehungswissenschaft und Psychologie, Clinical-Psychological Intervention<br>Ilkkursun, Zeynep; Koç University<br>Jordans, Mark J. D.; War Child; University of Amsterdam<br>Kiselev, Nikolai; University Hospital Zurich<br>Knaevelsrud, Christine; Freie Universität Berlin, Department of Clinical Psychological Intervention and Psychotherapy<br>Kurt, Gulsah; Koç University |

Martinmaki, Saara; ARQ National Psychotrauma Centre
McDaid, David ; LSE, PSSRU
Morina, Naser; University Hospital Zurich
Naser, Hadeel; International Medical Corps
Park, A-La; LSE, PSSRU
Pfaltz, Monique; University Hospital Zurich; Mid Sweden University
Roberts, Bayard; London School of Hygiene and Tropical Medicine,
Schick, Matthis; University Hospital Zurich
Schnyder, Ulrich; University Hospital Zurich, Department of Psychiatry and Psychotherapy
Spaaij, Julia; University Hospital Zurich
Steen, Frederik; War Child
Taha, Karine; War Child
Uygun, Ersin; Istanbul Bilgi University
Ventevogel, Peter; UNHCR Geneva, Public Health
Witteveen, Anke; Vrije Universiteit Amsterdam, Department of Clinical, Neuro- and Developmental Psychology and World Health Organization Collaborating Center for Research and Dissemination of Psychological Interventions
Whitney, Claire; International Medical Corps
Sijbrandij, Marit; Vrije Universiteit Amsterdam, Department of Clinical, Neuro- and Developmental Psychology and World Health Organization Collaborating Center for Research and Dissemination of Psychological Interventions

Keywords: MENTAL HEALTH, Anxiety disorders < PSYCHIATRY, Depression & mood disorders < PSYCHIATRY

SCHOLARONE™ Manuscripts

# Scalable Psychological Interventions for Syrian Refugees in Europe and the Middle East: STRENGTHS Study Protocol for a Prospective Individual Participant Data Meta-Analysis

Anne M. de Graaff (a.m.de.graaff@vu.nl),[1] Pim Cuijpers (p.cuijpers@vu.nl),[1] Ceren Acarturk (cacarturk@ku.edu.tr),[2] Aemal Akhtar (a.akhtar@unsw.edu.au),[3] Mhd Salem Alkneme (salem.alkneme@fu-berlin.de),[4] May Aoun (may.aoun@warchild.nl),[5] Manar Awwad (mawwad@InternationalMedicalCorps.org),[6] Ahmad Y. Bawaneh (abawaneh@InternationalMedicalCorps.org),[6] Felicity L. Brown (felicity.brown@warchild.nl), [5, 7] Richard Bryant (r.bryant@unsw.edu.au),[3] Sebastian Burchert (s.burchert@fu-berlin.de),[4] Kenneth Carswell (carswellk@who.int),[8] Annelieke Drogendijk (a.drogendijk@arq.org),[9] Michelle Engels (mieng@rodekors.dk),[10] Daniela C. Fuhr (Daniela.Fuhr@lshtm.ac.uk),[11] Pernille Hansen (pehan@rodekors.dk),[10] Edith van 't Hof (vanthofe@who.int),[8] Luana Giardinelli (luana.giardinelli@gmail.com),[6] Mahmoud Hemmo (Mahmoud.Hemmo@usz.ch),[12] Jonas M. Hessling (j.hessling@fu-berlin.de),[4] Zeynep Ilkkursun (zilkkursun20@ku.edu.tr),[2] Mark J.D. Jordans (mark.jordans@warchild.nl),[5, 7] Nikolai Kiselev (Nikolai.Kiselev@usz.ch),[12] Christine Knaevelsrud (christine.knaevelsrud@fu-berlin.de),[4] Gulsah Kurt (gkurt18@ku.edu.tr),[2] Saara Martinmaki (s.martinmaki@impact.arq.org),[9] David McDaid (D.Mcdaid@lse.ac.uk),[13] Naser Morina (naser.morina@usz.ch),[12] Hadeel Naser (hnaser@InternationalMedicalCorps.org),[6] A-La Park (a.park@lse.ac.uk),[13] Monique C. Pfaltz (Monique.Pfaltz@usz.ch) [12, 14] Bayard Roberts (bayard.roberts@lshtm.ac.uk),[11] Matthis Schick (matthis.schick@usz.ch),[12] Ulrich Schnyder (ulrich.schnyder@access.uzh.ch),[12] Julia Spaaij (julia.spaaij@usz.ch),[12] Frederik Steen (frederik.steen@warchild.nl),[5] Karine Taha (karine.taha@warchild.nl),[5] Ersin Uygun (ersin.uygun@bilgi.edu.tr),[15] Peter Ventevogel (ventevog@unhcr.org),[16] Anke Witteveen (a.b.witteveen@vu.nl),[1] Claire Whitney (cwhitney@InternationalMedicalCorps.org),[6] Marit Sijbrandij (e.m.sijbrandij@vu.nl),[1] on behalf of the STRENGTHS consortium

For peer review only


For peer review only - http://bmjopen.bmj.com/site/about/guidelines.xhtml

[1] Department of Clinical, Neuro- and Developmental Psychology, WHO Collaborating Center for Research and Dissemination of Psychological Interventions, Amsterdam Public Health Institute, Vrije Universiteit Amsterdam, Amsterdam, The Netherlands

[2] Koc University, Department of Psychology, Istanbul, Turkey

[3] School of Psychology, University of New South Wales, Sydney, Australia

[4] Division of Clinical-Psychological Intervention, Department of Education and Psychology, Freie Universität Berlin, Berlin, Germany

[5] Research and Development Department, War Child Holland, Amsterdam, The Netherlands

[6] Technical Unit, International Medical Corps, London, UK

[7] Amsterdam Institute of Social Science Research, University of Amsterdam, Amsterdam, the Netherlands

[8] Department of Mental Health and Substance Use, World Health Organization, Geneva, Switzerland

[9] ARQ National Psychotrauma Centre, ARQ International, Diemen, The Netherlands

[10] International Federation of Red Cross and Red Crescent Societies Reference Centre for Psychosocial Support, Copenhagen, Denmark

[11] London School of Hygiene and Tropical Medicine, Department of Health Services Research and Policy, London, UK

[12] University Hospital Zurich, Department of Consultation-Liaison Psychiatry and Psychosomatic Medicine, Zurich, Switzerland

[13] Care Policy and Evaluation Centre, Department of Health Policy, London School of Economics and Political Science, London, UK

[14] Department of Psychology and Social Work, Mid Sweden University, Ostersund, Sweden

[15] Bilgi University, Trauma and Disaster Mental Health Master Programme, Istanbul, Turkey

[16] United Nations High Commissioner for Refugees, Public Health Section, Genève, Switzerland


For peer review only - http://bmjopen.bmj.com/site/about/guidelines.xhtml

**Corresponding author:** Anne M. de Graaff, a.m.de.graaff@vu.nl, Department of Clinical, Neuro- and Developmental Psychology, WHO Collaborating Center for Research and Dissemination of Psychological Interventions, Amsterdam Public Health Research Institute, Vrije Universiteit Amsterdam, Van der Boechorststraat 7, 1081 BT, Amsterdam, The Netherlands

**Word count**: 3980

**Abstract word count**: 272

For peer review only


For peer review only - http://bmjopen.bmj.com/site/about/guidelines.xhtml

## Abstract

### Introduction

The World Health Organization's (WHO) scalable psychological interventions, such as Problem Management Plus (PM+) and Step-by-Step are designed to be cost-effective non-specialist delivered interventions to reduce symptoms of common mental disorders, such as anxiety, depression and posttraumatic stress disorder. The STRENGTHS research consortium aims to evaluate the effectiveness and cost-effectiveness of the individual format of PM+ and its group version (gPM+), as well as of the digital SbS intervention among Syrian refugees in seven countries in Europe and the Middle East. This is a study protocol for a prospective individual participant data (IPD) meta-analysis to evaluate 1) overall effectiveness and cost-effectiveness, and 2) treatment moderators of PM+, gPM+ and SbS with Syrian refugees.

### Methods and analysis

Five pilot RCTs and seven fully-powered RCTs conducted within STRENGTHS will be combined into one IPD meta-analytic dataset. The RCTs include Syrian refugees and other Arabic-speaking refugees of 18 years and above with elevated psychological distress (Kessler Psychological Distress Scale (K10 >15)) and impaired daily functioning (WHO Disability Assessment Schedule 2.0 (WHODAS 2.0 >16)), and are randomized into the intervention arm or care as usual control arm. We will conduct a one-stage IPD meta-analysis including only Syrian refugees using linear mixed models to evaluate overall effectiveness, cost-effectiveness and treatment moderators. Quality of evidence will be assessed using the Grading of Recommendations, Assessment, Development and Evaluation (GRADE) approach, and the economic evaluation approach will be assessed using the CHEC-list.

### Ethics and dissemination

Local ethical approval has been obtained for each RCT. This IPD meta-analysis does not require ethical approval. The results of this study will be published in peer-reviewed journals.

### Registration


For peer review only - http://bmjopen.bmj.com/site/about/guidelines.xhtml

Pre-registered on OSF: DOI 10.17605/OSF.IO/WUHGF

**Strengths and limitations of this study**

- We will conduct a prospective individual participant data meta-analysis to investigate the overall effectiveness of three scalable psychological interventions targeting psychological distress in Syrian refugees.
- We will perform moderation analyses to identify which individuals are more or less likely to benefit from scalable psychological interventions.
- Selected randomized controlled trials are currently being conducted as part of the STRENGTHS research consortium, and are intentionally similar in study design, outcome measures and target population to allow for optimal pooling of data.
- The meta-analyses will have reduced power if the number of observations for specific variables are not available across all studies.

**Key words:** Mental Health; Anxiety Disorders; Depression and Mood Disorders

For peer review only


For peer review only - http://bmjopen.bmj.com/site/about/guidelines.xhtml

## Introduction

In 2019, the world counted 54 active state-based armed conflicts (Strand et al., 2020). These armed conflicts, as well as other disasters such as natural hazards (e.g., hurricanes, floods) (Augustinavicius et al., 2021), are the main drivers for the 82.4 million individuals who are forcibly displaced worldwide (UNHCR, 2021a). About 26 million are refugees who are, due to their exposure to potentially traumatic events and other stressors before, during, and after displacement at risk of developing mental disorders (Schick et al., 2016; Steel et al., 2009; UNHCR, 2021a). The majority of refugees reside in low- and middle-income countries (LAMICs) (UNHCR, 2020), where health systems are often under-resourced to respond to the mental health needs of refugee populations (Fuhr et al., 2020; WHO, 2013).

Recognizing the gaps in health service availability in LAMICs and the mental health needs of underserved communities, including refugee populations, the World Health Organization (WHO) launched the Mental Health Gap Action Programme (mhGAP) to improve access to mental health and psychosocial support (MHPSS) in resource-constrained settings (WHO, 2008). As part of the mhGAP programme, the WHO is developing and testing a range of psychological interventions to scale up coverage for priority conditions, including depression and anxiety (WHO, 2017). Scalable interventions are strongly protocolized and use evidence-based psychological techniques from therapies such as cognitive behavioural therapy (CBT) and interpersonal therapy (IPT). Typical features include the duration of the intervention (i.e., few sessions instead of lengthy treatment programs), the target of the intervention (i.e., transdiagnostic instead of focused on a single disorder), and the delivery agent (i.e., task-shifting to non-specialist providers) (Singla et al., 2017; WHO, 2017).

One of the scalable interventions developed by the WHO is Problem Management Plus (PM+) which can be delivered by trained and supervised non-specialists to reduce psychological distress in communities affected by adversity (Dawson et al., 2015). The first randomized controlled trials (RCTs) on individual and group versions of PM+ have been conducted in communities affected by


For peer review only - http://bmjopen.bmj.com/site/about/guidelines.xhtml

violence in Pakistan and Kenya (Bryant et al., 2017; Rahman et al., 2016, 2019), and in a disaster-prone community in Nepal (Jordans et al., 2021). Individual PM+ led to improvements in anxiety, depression, posttraumatic stress disorder (PTSD), psychosocial functioning, and self-identified problems three months after the intervention (Bryant et al., 2017; Rahman et al., 2016). Furthermore, cost-effectiveness analysis of PM+ in addition to care as usual (CAU) was found to be costlier compared to CAU alone (Hamdani et al., 2020). PM+ delivered in groups (gPM+) to women in Pakistan was effective in reducing symptoms of anxiety, depression, and self-identified problems, but not for symptoms of PTSD (Rahman et al., 2019). In Nepal, gPM+ for men and women was also associated with improvements in psychological distress, depression, and "heart-mind" problems, but not for psychosocial functioning and symptoms of PTSD (Jordans et al., 2021). Another non-specialist delivered intervention based on PM+ that is currently being tested is the online Step-by-Step (SbS) intervention (Carswell et al., 2018). SbS is a minimally guided e-mental health intervention with 'e-helper' support. Pilot studies on SbS in Lebanon, including Syrian participants, found that the intervention was acceptable (Harper Shehadeh et al., 2020), and likely effective in improving psychosocial outcomes (Heim et al., 2021).

The Syrian REfuGees MeNTal HealTH Care Systems (STRENGTHS) research consortium evaluates the effectiveness, cost-effectiveness, and implementation of scalable interventions among Syrian refugees in countries in Europe and the Middle-East (Akhtar et al., 2020; de Graaff et al., 2020; Sijbrandij et al., 2017). Since the outbreak of the civil war in Syria, over 6.5 million people from Syria have sought refuge. The majority fled to neighbouring countries, such as Turkey (3.6 million), Lebanon (910,600), and Jordan (654,700); another one million to Europe by boat or overland (UNHCR, 2020). Refugees often reside in unstable and insecure locations in urban areas or refugee camps (Alshawawreh et al., 2017; Silove et al., 2017; UNHCR, 2021b). The COVID-19 pandemic further worsened living circumstances in overcrowded camps and urban settlements without opportunity to self-isolate and with limited access to health and welfare services (Iacobucci, 2020).


For peer review only - http://bmjopen.bmj.com/site/about/guidelines.xhtml

Although the WHO's scalable interventions have been developed for under-resourced settings specifically, such interventions might also be potentially (cost-)effective solutions for underserved communities in high-income settings (Sijbrandij et al., 2017). The rapidly escalated refugee crisis poses challenges for health systems in well-resourced settings in Europe (Lebano et al., 2020), where mental health services are underutilized due to major barriers such as communication difficulties and fear of stigma, and access is hampered by long wait lists (Kiselev et al., 2020; Satinsky et al., 2019).

Pilot studies among Syrian refugees indicated that PM+ is an acceptable and feasible intervention, and likely effective in reducing psychological distress (Acarturk et al., *submitted*; Akhtar et al., 2021b; de Graaff et al., 2020; Spaaij et al., *submitted*). Currently, fully-powered RCTs are being conducted among adult Syrian refugees in seven countries in Europe and the Middle East to test the effectiveness of PM+ in individual format in Switzerland and the Netherlands (de Graaff et al., 2020), PM+ in group format (gPM+) in Jordan (Akhtar et al., 2020) and Turkey (Uygun et al., 2020), and the digital intervention SbS in Germany, Sweden and Egypt (Sijbrandij et al., 2017). Initial results of PM+ delivered by trained peer-refugees to individuals and groups are promising. However, individual differences in symptom severity, socio-demographic background, comorbidities, living circumstances, and life events may result in individual differences in treatment response. Single trials do not have sufficient power to detect genuine interaction effects between participant characteristics and treatment outcome (Riley et al., 2020). To determine which individuals are more or less likely to benefit from these scalable psychological interventions, an individual participant data (IPD) meta-analytic approach can be used (Berlin et al., 2002; Fisher et al., 2017). This approach does not rely on aggregate (study-level) data used in conventional meta-analysis techniques, but uses the original data of each individual participant (participant-level) from eligible RCTs. The IPD meta-analysis approach offers greater statistical power and precision to better understand the predictive nature of individual characteristics on treatment outcome (Riley et al., 2020; Stewart & Parmar, 1993). Furthermore, it also allows us to


For peer review only - http://bmjopen.bmj.com/site/about/guidelines.xhtml

get a better estimate of overall effectiveness and cost-effectiveness, by pooling several large data sets together.

This protocol outlines a planned IPD meta-analysis on combined datasets from the STRENGTHS consortium, aiming to examine 1) the overall effectiveness and cost-effectiveness of scalable psychological interventions (i.e., PM+, gPM+, SbS) as compared to CAU alone on improving depression and anxiety (primary outcomes), PTSD symptoms, psychosocial functioning, and self-identified problems (secondary outcomes), and 2) the moderating effects of participants' sociodemographic, migratory, and clinical characteristics on treatment outcome (i.e., treatment-covariate interactions) of scalable psychological interventions as compared to CAU alone with Syrian adult refugees.

**Methods**

The protocol is registered in Open Science Framework (DOI 10.17605/OSF.IO/WUHGF). The PRISMA-P checklist (Moher et al., 2015) is appended.

**Identification of eligible studies**

We will include all STRENGTHS randomized controlled trials (RCTs) on the effectiveness of PM+ for individuals (Switzerland and the Netherlands), PM+ for groups (gPM+) (Jordan and Turkey), and SbS (Sweden, Germany and Egypt). These RCTs involve a relatively homogeneous population, and have similar study designs and outcomes measures.

The dates of the trials are April 2018-March 2019 [pilot RCT] and February 2019-ongoing [fully-powered RCT] (Netherlands), December 2018-March 2019 [pilot RCT] and February 2020-ongoing [fully-powered RCT] (Switzerland), January 2019-March 2019 [pilot RCT], October 2019-July 2020 [fully-powered RCT] (Jordan), September 2018-January 2019 [pilot RCT] and August 2019-ongoing [fully-powered RCT] (Turkey), August 2020-ongoing [fully-powered RCT] (Sweden),


For peer review only - http://bmjopen.bmj.com/site/about/guidelines.xhtml

December 2019-October 2020 [pilot RCT], August 2020-ongoing [fully-powered RCT] (Germany), March 2021-ongoing [fully-powered RCT] (Egypt).

**Participants and procedure**

In the trials, Arabic-speaking Syrian refugees of 18 years and above are included if they report psychological distress (Kessler Psychological Distress Scale (K10 >15)) (Kessler et al., 2002) and impaired daily functioning (WHO Disability Assessment Schedule 2.0 (WHODAS 2.0 >16)) (WHO, 2010). The trials on PM+ in Switzerland and the Netherlands also include Arabic-speaking refugees from other countries. In Jordan, an additional criterion is having a child between the ages 10-16 years.

Across trials, participants are excluded if there are signs of imminent suicide risk (PM+ manual questionnaire) (WHO, 2016). Furthermore, all trials except those on SbS in Sweden, Germany and Egypt exclude participants with acute medical conditions, expressed acute needs or protection risks, and indications of severe mental disorders (e.g., psychotic disorders or substance-dependence) or cognitive impairment (e.g., severe intellectual disability or dementia) (PM+ manual observation checklist) (WHO, 2016). The fully-powered PM+ trials in Switzerland and the Netherlands also exclude participants currently receiving specialized psychological treatment. Other exclusion criteria in Switzerland include being under guardianship, and inability to follow study procedures.

For the IPD meta-analysis, we will include only Syrian refugees. The RCTs in Turkey, Switzerland and the Netherlands are conducted in a community setting, while the RCTs in Jordan are conducted in a refugee camp. At this moment, all pilot trials have been completed, with sample sizes ranging from 46 to 64 (total $N$=302), while all fully-powered RCTs are still ongoing. Recruitment has completed for the fully-powered trials in Turkey ($N$=369), Jordan ($N$=410), and Egypt ($N$=537). All trials randomise individuals 1:1 to the intervention (PM+/gPM+/SbS + CAU) or control group (CAU only). The trials include assessments at baseline, one-week after the intervention, three months after the intervention, and 12 months after baseline. The 12-month assessment is only included in the fully-


For peer review only - http://bmjopen.bmj.com/site/about/guidelines.xhtml

powered RCTs. The pilot RCT in Jordan only included a baseline and one-week follow-up assessment. Treatment conditions are further explained below.

## The interventions

The manuals for all interventions were adapted by the STRENGTHS consortium according to a framework for the cultural adaptation of psychological interventions (Bernal & Sáez-Santiago, 2006), and materials to provide training for trainers and helpers/facilitators were developed (Akhtar et al., 2021a; Burchert et al., 2019).

## Problem Management Plus (PM+) for individuals and groups

PM+ is a brief, transdiagnostic psychological intervention developed to reduce symptoms of common mental disorders such as depression, anxiety, and PTSD (Dawson et al., 2015). PM+ includes five weekly sessions provided by trained non-specialist helpers. During this 5-week intervention, the participant learns skills in problem-solving, arousal reduction, behavioural activation and accessing social support. These strategies include a relaxation exercise using slow breathing (session 1), a 7-step plan to manage practical problems (session 2), behavioural activation by re-engaging with pleasant and task-oriented activities (session 3), and a strategy to strengthen social support (session 4). Homework is scheduled between sessions and strategies are reviewed in each subsequent session. Session 5 focuses on relapse prevention. The individual PM+ intervention consists of five 90-minute sessions. The group PM+ intervention consists of five 2-hour sessions in groups of 6-10 participants of the same gender led by two gender-matched group facilitators (Akhtar et al., 2021a; Dawson et al., 2015).

PM+ is delivered by Arabic-speaking peer-refugees who have completed high school, and have a background in education, social work, health care, or another related field. In Jordan, group facilitators could also be members of the host community. Helpers/group facilitators in Switzerland,


For peer review only - http://bmjopen.bmj.com/site/about/guidelines.xhtml

the Netherlands and Turkey also needed to be proficient in English or the local language (i.e., German, Dutch or Turkish). Helpers/group facilitators received and eight-day training of helpers (ToH) on common mental disorders, basic counselling skills, delivery of intervention strategies and self-care. The ToH was followed by practice cases. Helpers/group facilitators receive weekly supervision by trained PM+ trainers and supervisors throughout the trial. PM+ trainers/supervisors are mental health care professionals who received a five-day training of trainers (ToT) on elements covered in the ToH, as well as on training and supervision skills. They receive monthly supervision by a PM+ master trainer (Dawson et al., 2015).

**Step-by-Step (SbS)**

The SbS intervention is a 5-session, digital self-help intervention for iOS, Android and Web platforms, aimed at reducing depressive symptoms primarily through behavioural activation with additional components including psychoeducation, stress management, identifying strengths, positive self-talk, increasing social support and relapse prevention. WHO removed problem-solving from SbS, as it was found to be too complex to implement in a digital application with or without helper support (Carswell et al., 2018). The intervention uses an illustrated, narrative story of a fictional character that seeks help for depression from a professional. The narrative provides educative information on the strategies, supplemented by interactive exercises (e.g., activity scheduling, slow breathing). Sessions last 30 minutes, split into two parts, with optional contact-on-demand to non-specialist, Arabic-speaking "e-helpers" available through a text and audio messaging system. E-helpers are university students trained and supervised by mental health professionals on basic helping skills, providing basic support, and on the identification, assessment and management of risk and (serious) adverse events.

**Care as usual (CAU)**

For peer review only


For peer review only - http://bmjopen.bmj.com/site/about/guidelines.xhtml

The identified studies compared PM+, gPM+ or SbS in addition to CAU, to CAU alone. CAU includes all (mental) health services available to refugees across settings. In HICs, this includes referral to (specialized) mental health services through primary care practitioners (e.g., general practitioner). In Turkey, refugees have free access to mental health services in public hospitals and migrant health centres. Refugees residing in refugee camps in Jordan can access services inside the camp provided by national and international non-governmental organisations. However, across sites, access to mental health services is hampered by numerous barriers, such as the lack of Arabic-speaking health providers in European countries, and overburdened health systems of neighbouring countries hosting most refugees (Kiselev et al., 2020; Satinsky et al., 2019). All study participants received information about available mental health services.

**Outcomes**

In the planned IPD meta-analyses, our primary outcomes will be symptoms of depression and anxiety at 3-month follow-up, measured by the subscales of the Hopkins Symptom Checklist (HSCL-25) (Derogatis et al., 1974). Secondary outcomes include daily functioning (WHODAS 2.0), posttraumatic stress disorder symptoms (PTSD Checklist for DSM-5 (PCL-5)) (Blevins et al., 2015), and self-identified problems (PSYCHLOPS) (Ashworth et al., 2004). To calculate cost-effectiveness, in addition to documented resources required for intervention delivery, we will examine the pooled changes in resource use associated with use of health-related services, and time out of usual activities that have been collected using an adapted version of the Client Service Receipt Inventory (CSRI)) (Beecham & Knapp, 1992).

**Study-level variables**

We will obtain study-level data for each trial including country and setting in which the study was conducted, and intervention type (e.g., PM+ gPM+, SbS).


For peer review only - http://bmjopen.bmj.com/site/about/guidelines.xhtml

### Individual-level variables

We will extract individual-level data from each dataset to test a range of variables that may moderate the effects of the interventions on primary and secondary outcomes, to determine differential treatment effects based on participant characteristics. We will gather and synthesize all socio-demographic variables (i.e., age, gender, education level, work status, marital status), clinical characteristics (i.e., symptoms of depression, anxiety and PTSD, daily functioning, the exposure and type of traumatic events, post-migration stressors), and migration variables (i.e., time spent in host country, refugee status).

### IPD collection and aggregation

All research teams have agreed to contribute RCT data to these IPD meta-analyses. De-identified primary datasets will be made available through Castor EDC (Ciwit BV, 2018), an online software package used to monitor RCTs. Data will be stored in a secure cloud service (Surfdrive) developed for the Dutch education and research community, which can only be accessed by the IPD meta-analysis research team. Data transfer, storage and handling will follow the EU General Data Protection Regulations (GDPR).

Data accuracy of the primary datasets will be examined to explore if it matches data reported in published reports. We will specifically check frequencies of socio-demographic variables (e.g., age, gender, marital status, education level) and descriptive statistics (e.g., mean scores) of continuous scales. If discrepancies between the primary dataset and published report arise, clarification will be sought from authors. After the primary datasets are checked for accuracy, all eligible datasets will be merged into the IPD meta-analytic dataset.

### Quality of evidence


For peer review only - http://bmjopen.bmj.com/site/about/guidelines.xhtml

We will assess the quality of evidence by applying the Grading of Recommendations, Assessment, Development and Evaluation (GRADE) approach (The GRADE Working Group, 2013). The GRADE approach is a system for rating the quality (i.e., high, moderate, low, and very low) applied to a body of evidence, and not to the individual studies. GRADE offers a transparent and structured process for developing and presenting evidence summaries and for carrying out the steps involved in developing recommendations. The GRADE approach consists of rating eight criteria, including five that may lead to rating down the quality of evidence (i.e., risk of bias, inconsistency, indirectness, imprecision, publication bias) and three that may lead to rating up (i.e., large effect, dose-response gradient, minimal influence of residual plausible confounding). Results will be summarized in a GRADE evidence profile (Balshem et al., 2011). For the economic analysis, we will assess the economic evaluation approach using the CHEC-list (Evers et al., 2005).

**The IPD meta-analysis**

All analyses will be conducted in R version 4.0.3 (R Core Team, 2021) using the packages metafor (Viechtbauer, 2010) and lme4 (Bates et al., 2015). The economic evaluation will be conducted in STATA statistical software (StataCorp, 2021). We will use a 'one-stage IPD meta-analytic' approach, in which the IPD of all identified studies will be merged into one large dataset. The resulting dataset is analysed in one step, as if all participants, clustered within studies, belonged to a single trial. This approach is preferred over the 'two-stage IPD meta-analytic' approach, in which aggregated summary points are pooled in an appropriate meta-analysis model. The one-stage approach allows for more advanced modelling of the moderators, can adopt more appropriate likelihood functions and has fewer assumptions (Burke et al., 2017).

The IPD meta-analysis will be conducted according to the intention-to-treat principle (i.e., all randomized participants will be included in the analyses). Missing values for outcome data will be estimated under the missing-at-random assumption, using multiple imputation (100 imputations). To

For peer review only


For peer review only - http://bmjopen.bmj.com/site/about/guidelines.xhtml

estimate the missing values, valid predictor variables such as individual clinical and socio-demographic variables will be used (e.g., distress levels at baseline, age, gender). Sensitivity analyses will be conducted on complete cases only (i.e., with outcome data) to test for differences between imputed and completed values, and with completers of the interventions only.

To examine the effects of PM+, gPM+ and SbS on primary and secondary outcomes at 3-month follow-up, we will perform a mixed effect linear regression model with random intercepts with each trial having a random effect and a fixed effect for the intervention and the outcome measures. The severity of depressive and anxiety symptoms, PTSD symptoms, daily functioning, and self-identified problems, and costs of care at the 3-month follow-up will be used as dependent variables, and condition (e.g., treatment versus control) will be the independent variable while adjusting for baseline scores. The standardized β coefficient indicates how many standard deviations (*SD*) the dependent variables (i.e., anxiety, depression, PTSD, daily functioning, self-identified problems, and costs of care) change per *SD* increase in the independent variable. Thus, the larger the β coefficient, the greater the effect of the independent variable on the respective dependent variable, with no association among the variables if β is 0.

To ensure robustness of our findings, the analysis of the primary outcomes will be repeated using the 'two-stage IPD meta-analytic approach'. In a two-stage approach, Cohen's *d* will be calculated for all outcomes for every single trial separately (step 1), and will then be combined to calculate the pooled effect sizes using the random-effects model (step 2) (Riley et al., 2010).

For the economic analysis incremental cost-effectiveness ratios comparing changes in effect and costs between the intervention group and controls on the primary outcome will also be estimated. Uncertainty in mean differences in intervention resource use as well as the use of health and other services between baseline and 3-month follow-up between the two groups will be accounted for using bias-corrected and accelerated bootstrapping. Purchasing power parity adjusted unit costs expressed in international dollars will be applied to resource use. Discounting will not be applied given the short


For peer review only - http://bmjopen.bmj.com/site/about/guidelines.xhtml

duration of the study. Cost-effectiveness acceptability curves (CEACs) will be generated to showing the likelihood that intervention is cost-effective at different willingness-to-pay thresholds.

**Participant- and study-level moderators**

We will test whether socio-demographic, clinical and migration variables moderate the effects of psychological interventions among Syrian refugees at 3-month follow-up. To examine the effects of potential moderators, we will add an interaction term between each moderator variable and condition on depressive and anxiety symptoms into the mixed-effects linear/logistic regression models. Each potential moderator variable will be added into separate bivariate models.

**Heterogeneity**

We will calculate the $I^2$ statistic to assess statistical heterogeneity indicated in percentages, with 0% indicating no heterogeneity, 25% low heterogeneity, 50% moderate heterogeneity, and 75% high heterogeneity (Higgins et al., 2003). We will also calculate 95% Confidence Intervals (CI) around $I^2$ using the non-central chi-squared-based approach to provide the full magnitude of heterogeneity (Orsini et al., 2005).

**Small sample bias**

The present study protocol describes a prospective IPD meta-analysis of RCTs conducted within the STRENGTHS project. We will examine small sample bias by inspecting the funnel plot in which sample size is plotted against the effect estimates of the primary outcome measures. Egger's test of the intercept will be carried out to inspect the degree of asymmetry of the plot (Egger et al., 1997). Lastly, we will estimate the effect size adjusted for bias using the trim and fill procedure (Duval & Tweedie, 2000).


For peer review only - http://bmjopen.bmj.com/site/about/guidelines.xhtml

## Discussion

Scalable psychological interventions such as PM+, gPM+ and SbS for communities affected by adversity have been found to be effective in various RCTs (Bryant et al., 2017; Jordans et al., 2021; Rahman et al., 2016, 2019). Pilot studies on PM+ adapted for Syrian refugees indicated the intervention's feasibility and likely effectiveness when delivered by peer-refugees to individuals (de Graaff et al., 2020; Spaaij et al., *submitted*) or groups (Acarturk et al., *submitted*; Akhtar et al., 2021b). The STRENGTHS consortium tests the effectiveness of different types of scalable interventions (i.e., PM+, gPM+ and SbS) among Syrian refugees across different settings (e.g., refugee camp, community setting). The present paper describes the procedures of an IPD meta-analysis aimed at examining the overall effectiveness and cost-effectiveness of the interventions compared with CAU alone, and at examining individual participant differences in treatment response.

This study protocol has several strengths. An essential advantage of IPD meta-analytic approaches over using conventional meta-analytic approaches concerns the acquired power to study overall effect estimates, predictors and moderators, by synthesizing the original data from all individual RCTs into one large dataset. Single RCTs frequently do not have enough statistical power to investigate moderators. In conventional meta-analysis, moderation analyses are commonly done using subgroup analyses of aggregate data (e.g., effect sizes), but this limits statistical power and accuracy due to a loss of degrees of freedom and variability in the moderator of interest (Burke et al., 2017). IPD meta-analysis also allows us to investigate outcome variables that primary studies have not reported. Another strength of our protocol concerns the availability of study trials with similar population, study design and outcome variables. This enables us to determine who benefits more or less from scalable psychological interventions using a meta-analytic approach that is least biased and most reliable for addressing this question.

This study may have limitations. Overall statistical power may be reduced if the number of observations for specific variables are not available across all studies. Although the target group


For peer review only - http://bmjopen.bmj.com/site/about/guidelines.xhtml

comes from Syria, study participants reside in different settings, ranging from refugee camps with limited access to mental health care, to community settings in high-income countries where specialist services are widely available although not always easily accessible to refugees. Migratory patterns as well as the ongoing stressors might differ considerably across settings (Drescher et al., 2021; UNHCR, 2020).

This IPD meta-analysis protocol will extend our current knowledge on the effectiveness of scalable psychological interventions for refugees and other populations affect by adversity. It will allow for more accurate estimations of treatment effects, cost-effectiveness, and the exploration of important treatment moderators of scalable interventions for sub-groups of refugees in Europe and the Middle East. The results of this study will be disseminated through publication in peer-reviewed journals.

**Authors' contributions**

AG drafted the study protocol. All authors provided input for the protocol writing, and read and approved the final version. MSij is the guarantor of the review.

**Funding**

The STRENGTHS project is funded under Horizon 2020 – the Framework Programme for Research and Innovation (2014-2020). The authors alone are responsible for the views expressed in this article and they do not necessarily represent the views, decisions or policies of the institutions with which they are affiliated. The European Community is not liable for any use that may be made of the information contained therein.

**Competing interests**

None declared.


For peer review only - http://bmjopen.bmj.com/site/about/guidelines.xhtml

## Patient consent for publication

Not required.

## ORCID IDs

Anne M. de Graaff https://orcid.org/0000-0001-6686-4432

Pim Cuijpers https://orcid.org/0000-0001-5497-2743

Ceren Acarturk https://orcid.org/0000-0001-7093-1554

Aemal Akhtar https://orcid.org/0000-0002-8510-3636

Mhd Salem Alkneme https://orcid.org/0000-0002-4593-6705

Felicity Brown: https://orcid.org/0000-0001-6800-1657

Richard Bryant https://orcid.org/0000-0002-9607-819X

Sebastian Burchert https://orcid.org/0000-0003-3126-5485

Kenneth Carswell: https://orcid.org/0000-0001-5344-5802

Michelle Engels https://orcid.org/0000-0002-3782-1609

Daniela C. Fuhr https://orcid.org/0000-0001-9020-4629

Pernille Hansen https://orcid.org/0000-0002-4782-458X

Mahmoud Hemmo https://orcid.org/0000-0002-2958-0702

Edith van 't Hof https://orcid.org/0000-0002-5856-3573

Zeynep Ilkkursun https://orcid.org/0000-0001-6691-1809

Mark J.D. Jordans https://orcid.org/0000-0001-5925-8039

Nikolai Kiselev https://orcid.org/0000-0003-0617-0353

Christine Knaevelsrud https://orcid.org/0000-0003-1342-7006

Saara E. Martinmäki https://orcid.org/0000-0003-0230-4641

David McDaid https://orcid.org/0000-0003-0744-2664

For peer review only


For peer review only - http://bmjopen.bmj.com/site/about/guidelines.xhtml

Naser Morina https://orcid.org/0000-0002-6470-4408

A-La Park https://orcid.org/0000-0002-4704-4874

Monique Pfaltz https://orcid.org/0000-0002-7762-6226

Ulrich Schnyder https://orcid.org/0000-0003-3556-7990

Marit Sijbrandij https://orcid.org/0000-0001-5430-9810

Peter Ventevogel https://orcid.org/0000-0002-3567-8861

Claire Whitney https://orcid.org/0000-0002-1100-5363

Anke Witteveen https://orcid.org/0000-0002-9636-7522

For peer review only



For peer review only - http://bmjopen.bmj.com/site/about/guidelines.xhtml

## Literature

Akhtar, A., Engels, M. H., Bawaneh, A., Bird, M., Bryant, R., Cuijpers, P., Hansen, P., & Al-hayek, H. (2021). Cultural Adaptation of a Low-Intensity Group Psychological Intervention for Syrian Refugees. *Intervention*, *19*(1), 48–57. https://doi.org/10.4103/INTV.INTV

Akhtar, A., Giardinelli, L., Bawaneh, A., Awwad, M., Al-Hayek, H., Whitney, C., Jordans, M. J. D., Sijbrandij, M., Cuijpers, P., Dawson, K., & Bryant, R. (2021). Feasibility trial of a scalable transdiagnostic group psychological intervention for Syrians residing in a refugee camp. *European Journal of Psychotraumatology*, *12*, 1932295. https://doi.org/10.1080/20008198.2021.1932295

Akhtar, A., Giardinelli, L., Bawaneh, A., Awwad, M., Naser, H., Whitney, C., Jordans, M. J. D., Sijbrandij, M., & Bryant, R. A. (2020). Group problem management plus (gPM+) in the treatment of common mental disorders in Syrian refugees in a Jordanian camp: Study protocol for a randomized controlled trial. *BMC Public Health*, *20*(1), 1–8. https://doi.org/10.1186/s12889-020-08463-5

Alshawawreh, L. A., Smith, R. S., & Wood, J. B. (2017). Assessing the Sheltering Response in the Middle East: Studying Syrian Camps in Jordan. *International Scholarly and Scientific Research & Innovation*, *11*(8), 1751–1757. https://reliefweb.int/sites/reliefweb.int/files/resources/01 Azraq Fact Sheet January 2021.pdf

Ashworth, M., Shepher, M., Christey, J., Matthews, V., Wright, K., Parmentier, H., Robinson, S., & Godfrey, E. (2004). A client-generated psychometric instrument: the development of "PSYCHLOPS." *Counseling and Psychotherapy Research*, *4*(2), 27–32. https://doi.org/10.1093/astrogeo/atw101

Augustinavicius, J. L., Lowe, S. R., Massazza, A., Hayes, K., Denckla, C., White, R. G., Caban-Aleman, C., Clayton, S., Verdeli, L., & Berry, H. (2021). *Global climate change and trauma: An*

For peer review only


For peer review only - http://bmjopen.bmj.com/site/about/guidelines.xhtml

*International Society for Traumatic Stress Studies Briefing Paper*.

Balshem, H., Helfand, M., Schünemann, H. J., Oxman, A. D., Kunz, R., Brozek, J., Vist, G. E., Falck-Ytter, Y., Meerpohl, J., Norris, S., & Guyatt, G. H. (2011). GRADE guidelines: 3. Rating the quality of evidence. *Journal of Clinical Epidemiology*, *64*(4), 401–406. https://doi.org/10.1016/j.jclinepi.2010.07.015

Bates, D., Mächler, M., Bolker, B. M., & Walker, S. C. (2015). Fitting linear mixed-effects models using lme4. *Journal of Statistical Software*, *67*(1). https://doi.org/10.18637/jss.v067.i01

Beecham, J., & Knapp, M. (1992). Costing psychiatric interventions. In G. Thornicroft, C. Brewin, & J. Wing (Eds.), *Measuring Mental Health Needs* (1st ed., pp. 163–183). Gaskell.

Berlin, J. A., Santanna, J., Schmid, C. H., Szczech, L. A., & Feldman, H. I. (2002). Individual patient-versus group-level data meta-regressions for the investigation of treatment effect modifiers: Ecological bias rears its ugly head. *Statistics in Medicine*, *21*(3), 371–387. https://doi.org/10.1002/sim.1023

Bernal, G., & Sáez-Santiago, E. (2006). Culturally centered psychosocial interventions. *Journal of Community Psychology*, *34*(2), 121–132. https://doi.org/10.1002/jcop.20096

Blevins, C. A., Weathers, F. W., Davis, M. T., Witte, T. K., & Domino, J. L. (2015). The Posttraumatic Stress Disorder Checklist for DSM-5 (PCL-5): Development and initial psychometric evaluation. *Journal of Traumatic Stress*, *28*, 489–498. https://doi.org/10.1002/jts.22059

Bryant, R. A., Schafer, A., Dawson, K. S., Anjuri, D., Mulili, C., Ndogoni, L., Koyiet, P., Sijbrandij, M., Ulate, J., Harper Shehadeh, M., Hadzi-Pavlovic, D., & van Ommeren, M. (2017). Effectiveness of a brief behavioural intervention on psychological distress among women with a history of gender-based violence in urban Kenya: A randomised clinical trial. *PLoS Medicine*, *14*(8), e1002371. https://doi.org/https:// doi.org/10.1371/journal.pmed.1002371

Burchert, S., Alkneme, M. S., Bird, M., Carswell, K., Cuijpers, P., Hansen, P., Heim, E., Shehadeh, M.

For peer review only



For peer review only - http://bmjopen.bmj.com/site/about/guidelines.xhtml

H., Sijbrandij, M., Van't Hof, E., & Knaevelsrud, C. (2019). User-centered app adaptation of a low-intensity e-mental health intervention for Syrian refugees. *Frontiers in Psychiatry*, *10*(JAN). https://doi.org/10.3389/fpsyt.2018.00663

Burke, D. L., Ensor, J., & Riley, R. D. (2017). Meta-analysis using individual participant data: one-stage and two-stage approaches, and why they may differ. *Statistics in Medicine*, *36*(5), 855–875. https://doi.org/10.1002/sim.7141

Carswell, K., Harper-Shehadeh, M., Watts, S., van't Hof, E., Abi Ramia, J., Heim, E., Wenger, A., & van Ommeren, M. (2018). Step-by-Step: a new WHO digital mental health intervention for depression. *MHealth*, *4*, 34–34. https://doi.org/10.21037/mhealth.2018.08.01

Ciwit BV. (2018). *Castor Electronic Data Capture*.

Dawson, K. S., Bryant, R. A., Harper, M., Kuowei Tay, A., Rahman, A., Schafer, A., & Van Ommeren, M. (2015). Problem Management Plus (PM+): a WHO transdiagnostic psychological intervention for common mental health problems. *World Psychiatry*, *14*(3), 354–357. https://doi.org/10.1002/wps.20255

Dawson, K. S., Watts, S., Carswell, K., Shehadeh, M. H., Jordans, M. J., Bryant, R. A., Miller, K. E., Malik, A., Brown, F. L., Servili, C., & Van Ommeren, M. (2019). Improving access to evidence-based interventions for young adolescents: Early Adolescent Skills for Emotions (EASE). *World Psychiatry*, *18*(1), 105–107. https://doi.org/10.1002/wps.20597

de Graaff, A., Cuijpers, P., Acarturk, C., Bryant, R., Burchert, S., Fuhr, D., Huizink, A., Jong, J., Kieft, B., Knaevelsrud, C., McDaid, D., Morina, N., Park, A.-L., Uppendahl, J., Ventevogel, P., Whitney, C., Wiedemann, N., Woodward, A., & Sijbrandij, M. (2020). Effectiveness of a peer-refugee delivered psychological intervention to reduce psychological distress among adult Syrian refugees in the Netherlands: study protocol. *European Journal of Psychotraumatology*, *11*(1), 1694347. https://doi.org/10.1080/20008198.2019.1694347

de Graaff, A. M., Cuijpers, P., McDaid, D., Park, A., Woodward, A., Bryant, R. A., Fuhr, D. C., Kieft,

For peer review only


For peer review only - http://bmjopen.bmj.com/site/about/guidelines.xhtml

B., Minkenberg, E., & Sijbrandij, M. (2020). Peer-provided Problem Management Plus (PM+) for adult Syrian refugees; a pilot randomized controlled trial on effectiveness and cost-effectiveness. *Epidemiology and Psychiatric Sciences*, *29*(e126), 1–24.

Derogatis, L. R., Lipman, R. S., Rickels, K., Uhlenhuth, E. H., & Covi, L. (1974). The Hopkins Symptom Checklist (HSCL): A self-report inventory. *Behavioral Science*, *19*, 1–15.

Drescher, A., Kiselev, N., Akhtar, A., Acarturk, C., Bryant, R. A., Ilkkursun, Z., von Känel, R., Miller, K. E., Pfaltz, M. C., Schick, M., Schnyder, U., Sijbrandij, M., Spaaij, J., & Morina, N. (2021). Problems after flight: understanding and comparing Syrians' perspectives in the Middle East and Europe. *BMC Public Health*, *21*(1), 1–12. https://doi.org/10.1186/s12889-021-10498-1

Duval, S., & Tweedie, R. (2000). Trim and Fill: A Simple Funnel-Plot-Based Method. *Biometrics*, *56*(June), 455–463.

Egger, M., Smith, G. D., Schneider, M., & Minder, C. (1997). Bias in meta-analysis detected by a simple, graphical test. *BMJ*, *315*, 629–634.

Evers, S., Goossens, M., De Vet, H., Van Tulder, M., & Ament, A. (2005). Criteria list for assessment of methodological quality of economic evaluations: Consensus on Health Economic Criteria. *International Journal of Technology Assessment in Health Care*, *21*(2), 240–245. https://doi.org/10.1017/s0266462305050324

Fisher, D. J., Carpenter, J. R., Morris, T. P., Freeman, S. C., & Tierney, J. F. (2017). Meta-analytical methods to identify who benefits most from treatments: Daft, deluded, or deft approach? *BMJ (Online)*, *356*, 1–6. https://doi.org/10.1136/bmj.j573

Fuhr, D. C., Acarturk, C., McGrath, M., Ilkkursun, Z., Sondorp, E., Sijbrandij, M., Ventevogel, P., Cuijpers, P., McKee, M., & Roberts, B. (2020). Treatment gap and mental health service use among Syrian refugees in Sultanbeyli, Istanbul: a cross-sectional survey. *Epidemiology and Psychiatric Sciences*, *29*, e70. https://doi.org/10.1017/S2045796019000660

Hamdani, S. U., Huma, Z. E., Rahman, A., Wang, D., Chen, T., Van Ommeren, M., Chisholm, D., &

For peer review only


For peer review only - http://bmjopen.bmj.com/site/about/guidelines.xhtml

Farooq, S. (2020). Cost-effectiveness of WHO Problem Management plus for adults with mood and anxiety disorders in a post-conflict area of Pakistan: Randomised controlled trial. *British Journal of Psychiatry*, *217*(5), 623–629. https://doi.org/10.1192/bjp.2020.138

Harper Shehadeh, M. J., Abi Ramia, J., Cuijpers, P., El Chammay, R., Heim, E., Kheir, W., Saeed, K., van Ommeren, M., van't Hof, E., Watts, S., Wenger, A., Zoghbi, E., & Carswell, K. (2020). Step-by-Step, an E-Mental Health Intervention for Depression: A Mixed Methods Pilot Study From Lebanon. *Frontiers in Psychiatry*, *10*(February), 1–11. https://doi.org/10.3389/fpsyt.2019.00986

Heim, E., Ramia, J. A., Hana, R. A., Burchert, S., Carswell, K., Cornelisz, I., Cuijpers, P., El Chammay, R., Noun, P., van Klaveren, C., van Ommeren, M., Zoghbi, E., & van't Hof, E. (2021). Step-by-step: Feasibility randomised controlled trial of a mobile-based intervention for depression among populations affected by adversity in Lebanon. *Internet Interventions*, *24*, 100380. https://doi.org/10.1016/j.invent.2021.100380

Higgins, J. P. T., Thompson, S. G., Deeks, J. J., & Altman, D. G. (2003). Measuring inconsistency in meta-analyses. *British Medical Journal*, *327*(7414), 557–560. https://doi.org/10.1136/bmj.327.7414.557

Iacobucci, G. (2020). Covid-19: Doctors warn of humanitarian catastrophe at Europe's largest refugee camp. *BMJ (Clinical Research Ed.)*, *368*(March), m1097. https://doi.org/10.1136/bmj.m1097

Jacobson, N. S., & Truax, P. (1991). Clinical significance: A statistical approach to defining meaningful change in psychotherapy research. *Journal of Consulting and Clinical Psychology*, *59*(1), 12–19. https://doi.org/10.1037/0022-006X.59.1.12

Jordans, M. J. D., Kohrt, B. A., Sangraula, M., Turner, E. L., Wang, X., Shrestha, P., Ghimire, R., Van't Hof, E., Bryant, R. A., Dawson, K. S., Marahatta, K., Luitel, N. P., & Van OmmerenI, M. (2021). Effectiveness of Group Problem Management Plus, a brief psychological intervention for adults affected by humanitarian disasters in Nepal: A cluster randomized controlled trial. *PLoS*


For peer review only - http://bmjopen.bmj.com/site/about/guidelines.xhtml

*Medicine*, *18*(6), 1–22. https://doi.org/10.1371/journal.pmed.1003621

Kessler, R. C., Andrews, G., Colpe, L. J., Hiripi, E., Mroczek, D. K., Normand, S.-L. T., Walters, E. E., & Zaslavsky, A. M. (2002). Short screening scales to monitor population prevalences and trends in non-specific psychological distress. *Psychological Medicine*, *32*(6), 959–976. https://doi.org/10.1017/S0033291702006074

Kiselev, N., Pfaltz, M., Haas, F., Schick, M., Kappen, M., Sijbrandij, M., De Graaff, A. M., Bird, M., Hansen, P., Ventevogel, P., Fuhr, D. C., Schnyder, U., & Morina, N. (2020). Structural and socio-cultural barriers to accessing mental healthcare among Syrian refugees and asylum seekers in Switzerland. *European Journal of Psychotraumatology*, *11*(1). https://doi.org/10.1080/20008198.2020.1717825

Lebano, A., Hamed, S., Bradby, H., Gil-Salmerón, A., Durá-Ferrandis, E., Garcés-Ferrer, J., Azzedine, F., Riza, E., Karnaki, P., Zota, D., & Linos, A. (2020). Migrants' and refugees' health status and healthcare in Europe: A scoping literature review. *BMC Public Health*, *20*(1). https://doi.org/10.1186/s12889-020-08749-8

Moher, D., Shamseer, L., Clarke, M., Ghersi, D., Liberati, A., Petticrew, M., Shekelle, P., Stewart, L. A., & Group, P.-P. (2015). Preferred reporting items for systematic review and meta-analysis protocols (PRISMA-P) 2015 statement. *Systematic Reviews*, *4*(1), 1. https://doi.org/10.1186/2046-4053-4-1

Orsini, N., Bottai, M., Higgins, J., & Buchan, I. (2005). *Heterogi: Stata module to quantify heterogeneity in a meta-analysis* (revised 2006-01-25; p. S449201). Boston College Department of Economics.

R Core Team. (2021). *R: A language and environment for statistical computing*. R Foundation for Statistical Computing.

Rahman, A., Hamdani, S. U., Awan, N. R., Bryant, R. A., Dawson, K. S., Khan, M. F., Mukhtar-ul-Haq Azeemi, M., Akhtar, P., Nazir, H., Chiumento, A., Sijbrandij, M., Wang, D., Farooq, S., &


For peer review only - http://bmjopen.bmj.com/site/about/guidelines.xhtml

Van Ommeren, M. (2016). Effect of a multicomponent behavioral intervention in adults ipairment by psychological distress in a conflict-affected area of Pakistan. A randomized controlled trial. *JAMA*, *316*(24), 2609–2617. https://doi.org/10.1001/jama.2016.17165

Rahman, A., Khan, M. N., Hamdani, S. U., Chiumento, A., Akhtar, P., Nazir, H., Nisar, A., Masood, A., Din, I. U., Khan, N. A., Bryant, R. A., Dawson, K. S., Sijbrandij, M., Wang, D., & van Ommeren, M. (2019). Effectiveness of a brief group psychological intervention for women in a post-conflict setting in Pakistan: a single-blind, cluster, randomised controlled trial. *The Lancet*. https://doi.org/10.1016/S0140-6736(18)32343-2

Riley, R. D., Debray, T. P. A., Fisher, D., Hattle, M., Marlin, N., Hoogland, J., Gueyffier, F., Staessen, J. A., Wang, J., Moons, K. G. M., Reitsma, J. B., & Ensor, J. (2020). Individual participant data meta-analysis to examine interactions between treatment effect and participant-level covariates: Statistical recommendations for conduct and planning. *Statistics in Medicine*, *39*(15), 2115–2137. https://doi.org/10.1002/sim.8516

Riley, R. D., Lambert, P. C., & Abo-Zaid, G. (2010). Meta-analysis of individual participant data: Rationale, conduct, and reporting. *BMJ*, *340*(7745), 521–525. https://doi.org/10.1136/bmj.c221

Satinsky, E., Fuhr, D. C., Woodward, A., Sondorp, E., & Roberts, B. (2019). Mental health care utilisation and access among refugees and asylum seekers in Europe: A systematic review. *Health Policy*, *123*, 851–863. https://doi.org/10.1016/j.healthpol.2019.02.007

Schick, M., Zumwald, A., Knopfli, B., Nickerson, A., Bryant, R. A., Schnyder, U., Muller, J., & Morina, N. (2016). Challenging future, challenging past: The relationship of social integration and psychological impairment in traumatized refugees. *European Journal of Psychotraumatology*, *7*, 1–9. https://doi.org/10.3402/ejpt.v7.28057

Sijbrandij, M., Acarturk, C., Bird, M., Bryant, R. A., Burchert, S., Carswell, K., de Jong, J., Dinesen, C., Dawson, K. S., El Chammay, R., van Ittersum, L., Jordans, M., Knaevelsrud, C., McDaid, D., Miller, K., Morina, N., Park, A.-L., Roberts, B., van Son, Y., … Cuijpers, P. (2017).

For peer review only


For peer review only - http://bmjopen.bmj.com/site/about/guidelines.xhtml

Strengthening mental health care systems for Syrian refugees in Europe and the Middle East: integrating scalable psychological interventions in eight countries. *European Journal of Psychotraumatology*, *8*, 1388102. https://doi.org/10.1080/20008198.2017.1388102

Silove, D., Ventevogel, P., & Rees, S. (2017). The contemporary refugee crisis: an overview of mental health challenges. *World Psychiatry*, *16*(2), 130–139. https://doi.org/10.1002/wps.20438

Singla, D. R., Kohrt, B. A., Murray, L. K., Anand, A., Chorpita, B. F., & Patel, V. (2017). Psychological treatment for the world: Lessons from low- and middle-income countries. *Annual Review of Clinical Psychology*, *49*, 149–181. https://doi.org/10.1146/annurev-clinpsy-032816-045217.Psychological

Spaaij, J., Kiselev, N., Berger, C., Bryant, R., Cuijpers, P., de Graaff, A., Fuhr, D., Hemmo, M., McDaid, D., Moergeli, H., Park, A., Pfaltz, M., Schick, M., Schnyder, U., Wenger, A., Sijbrandij, M., & Morina, N. (n.d.). *Feasibility and acceptability of Problem Management Plus (PM+) among Syrian refugees in Switzerland: a pilot randomized controlled trial.*

StataCorp. (2021). *Stata Statistical Software* (Release 17). StataCorp LLC.

Steel, Z., Chey, T., Silove, D., Marnane, C., Bryant, R. A., & Van Ommeren, M. (2009). Association of torture and other potentially traumatic events with mental health outcomes among populations exposed to mass conflict and displacement: A systematic review and meta-analysis. *JAMA - Journal of the American Medical Association*, *302*(5), 537–549. https://doi.org/10.1001/jama.2009.1132

Sterne, J. A. C., Savović, J., Page, M. J., Elbers, R. G., Blencowe, N. S., Boutron, I., Cates, C. J., Cheng, H. Y., Corbett, M. S., Eldridge, S. M., Emberson, J. R., Hernán, M. A., Hopewell, S., Hróbjartsson, A., Junqueira, D. R., Jüni, P., Kirkham, J. J., Lasserson, T., Li, T., … Higgins, J. P. T. (2019). RoB 2: A revised tool for assessing risk of bias in randomised trials. *The BMJ*, *366*, 1–8. https://doi.org/10.1136/bmj.l4898

Stewart, L., & Parmar, M. (1993). Meta-analysis pof the literature or of individual patient data: is there

For peer review only


For peer review only - http://bmjopen.bmj.com/site/about/guidelines.xhtml

a difference? *Lancet*, *341*, 418–422.

Strand, H., Rustad, S. A., Nygard, H. M., & Hegre, H. (2020). *Trends in Armed Conflict, 1946-2019.*

The GRADE Working Group. (2013). *GRADE handbook for grading quality of evidence and strength of recommendations* (H. Schünemann, J. Brożek, G. Guyatt, & A. Oxman (eds.)). guidelinedevelopment.org/handbook

UNHCR. (2020). Trends at a glance: Global trends forced displacement in 2019. *UNHCR The UN Refugee Agency*, 1–84. https://www.unhcr.org/5ee200e37.pdf

UNHCR. (2021a). *Global Trends Forced Displacement in 2020.* https://www.unhcr.org/flagship-reports/globaltrends/

UNHCR. (2021b). *Zaatari Camp Fact Sheet* (Issue January). https://reliefweb.int/sites/reliefweb.int/files/resources/01 Zaatari Fact Sheet January 2021.pdf

Uygun, E., Ilkkursun, Z., Sijbrandij, M., Aker, A. T., Bryant, R., Cuijpers, P., Fuhr, D. C., De Graaff, A. M., De Jong, J., McDaid, D., Morina, N., Park, A. La, Roberts, B., Ventevogel, P., Yurtbakan, T., & Acarturk, C. (2020). Protocol for a randomized controlled trial: Peer-to-peer Group Problem Management plus (PM+) for adult Syrian refugees in Turkey. *Trials*, *21*(1), 1–9. https://doi.org/10.1186/s13063-020-4166-x

Viechtbauer, W. (2010). Conducting meta-analyses in R with the metafor. *Journal of Statistical Software*, *36*(3), 1–48. https://doi.org/10.18637/jss.v036.i03

WHO. (2008). Mental Health Gap Action Programme: scaling up care for mental, neurological and substance use disorders. In *mhGAP: Mental Health Gap Action Programme.* http://www.who.int/mental_health/evidence/mhGAP/en/

WHO. (2010). *Measuring Health and Disability: Manual for WHO Disability Assessment Schedule WHODAS 2.0* (T. B. Üstun, N. Kostansjek, S. Chatterji, & J. Rehm (eds.)). WHO.

WHO. (2013). Building Back Better: Sustainable Mental Health Car after Emergencies. In *World Health Organization.*


For peer review only - http://bmjopen.bmj.com/site/about/guidelines.xhtml

WHO. (2016). *Problem Management Plus (PM+). Individual psychological help for adults impaired by distress in communities exposed to adversity* (Generic fi). WHO. http://www.who.int/entity/mental_health/emergencies/problem_management_plus/en/index.html

WHO. (2017). *Scalable psychological interventions for people in communities affected by adversity. A new area of mental health and psychosocial work at WHO.*

Zahra, D., & Hedge, C. (2010). The reliable change index: Why isn't it more popular in academic psychology? *Psychology Postgraduate Affairs Group Quarterly*, *76*, 14–19.

For peer review only


For peer review only - http://bmjopen.bmj.com/site/about/guidelines.xhtml

## PRISMA-P (Preferred Reporting Items for Systematic review and Meta-Analysis Protocols) 2015 checklist: recommended items to address in a systematic review protocol*

| Section and topic | Item No | Checklist item | Page |
|---|---|---|---|
| **ADMINISTRATIVE INFORMATION** | | | |
| Title: | | | |
| Identification | 1a | Identify the report as a protocol of a systematic review | 1 |
| Update | 1b | If the protocol is for an update of a previous systematic review, identify as such | N/A |
| Registration | 2 | If registered, provide the name of the registry (such as PROSPERO) and registration number | |
| Authors: | | | |
| Contact | 3a | Provide name, institutional affiliation, e-mail address of all protocol authors; provide physical mailing address of corresponding author | 1-2 |
| Contributions | 3b | Describe contributions of protocol authors and identify the guarantor of the review | 14 |
| Amendments | 4 | If the protocol represents an amendment of a previously completed or published protocol, identify as such and list changes; otherwise, state plan for documenting important protocol amendments | N/A |
| Support: | | | |
| Sources | 5a | Indicate sources of financial or other support for the review | 14 |
| Sponsor | 5b | Provide name for the review funder and/or sponsor | 14 |
| Role of sponsor or funder | 5c | Describe roles of funder(s), sponsor(s), and/or institution(s), if any, in developing the protocol | 14 |
| **INTRODUCTION** | | | |
| Rationale | 6 | Describe the rationale for the review in the context of what is already known | 5-7 |
| Objectives | 7 | Provide an explicit statement of the question(s) the review will address with reference to participants, interventions, comparators, and outcomes (PICO) | 7 |
| **METHODS** | | | |
| Eligibility criteria | 8 | Specify the study characteristics (such as PICO, study design, setting, time frame) and report characteristics (such as years considered, language, publication status) to be used as criteria for eligibility for the review | 7-8 |
| Information sources | 9 | Describe all intended information sources (such as electronic databases, contact with study authors, trial registers or other grey literature sources) with planned dates of coverage | 7 |
| Search strategy | 10 | Present draft of search strategy to be used for at least one electronic database, including planned limits, such that it could be repeated | N/A |
| Study records: | | | |

For peer review only

| | | | |
|---|---|---|---|
| Data management | 11a | Describe the mechanism(s) that will be used to manage records and data throughout the review | 10 |
| Selection process | 11b | State the process that will be used for selecting studies (such as two independent reviewers) through each phase of the review (that is, screening, eligibility and inclusion in meta-analysis) | N/A |
| Data collection process | 11c | Describe planned method of extracting data from reports (such as piloting forms, done independently, in duplicate), any processes for obtaining and confirming data from investigators | 10 |
| Data items | 12 | List and define all variables for which data will be sought (such as PICO items, funding sources), any pre-planned data assumptions and simplifications | 10-12 |
| Outcomes and prioritization | 13 | List and define all outcomes for which data will be sought, including prioritization of main and additional outcomes, with rationale | 10 |
| Risk of bias in individual studies | 14 | Describe anticipated methods for assessing risk of bias of individual studies, including whether this will be done at the outcome or study level, or both; state how this information will be used in data synthesis | 11 |
| Data synthesis | 15a | Describe criteria under which study data will be quantitatively synthesised | 11-12 |
| | 15b | If data are appropriate for quantitative synthesis, describe planned summary measures, methods of handling data and methods of combining data from studies, including any planned exploration of consistency (such as $I^2$, Kendall's $\tau$) | 11-12 |
| | 15c | Describe any proposed additional analyses (such as sensitivity or subgroup analyses, meta-regression) | 11 |
| | 15d | If quantitative synthesis is not appropriate, describe the type of summary planned | N/A |
| Meta-bias(es) | 16 | Specify any planned assessment of meta-bias(es) (such as publication bias across studies, selective reporting within studies) | 12-13 |
| Confidence in cumulative evidence | 17 | Describe how the strength of the body of evidence will be assessed (such as GRADE) | 11 |

*** It is strongly recommended that this checklist be read in conjunction with the PRISMA-P Explanation and Elaboration (cite when available) for important clarification on the items. Amendments to a review protocol should be tracked and dated.** **The copyright for PRISMA-P (including checklist) is held by the PRISMA-P Group and is distributed under a Creative Commons Attribution Licence 4.0.**

*From: Shamseer L, Moher D, Clarke M, Ghersi D, Liberati A, Petticrew M, Shekelle P, Stewart L, PRISMA-P Group. Preferred reporting items for systematic review and meta-analysis protocols (PRISMA-P) 2015: elaboration and explanation. BMJ. 2015 Jan 2;349(jan02 1):g7647.*

For peer review only

BMJ Open

# Scalable Psychological Interventions for Syrian Refugees in Europe and the Middle East: STRENGTHS Study Protocol for a Prospective Individual Participant Data Meta-Analysis



| | |
|---|---|
| Journal: | *BMJ Open* |
| Manuscript ID | bmjopen-2021-058101.R1 |
| Article Type: | Protocol |
| Date Submitted by the Author: | 24-Jan-2022 |





| | |
|---|---|
| Complete List of Authors: | de Graaff, Anne; Vrije Universiteit Amsterdam, Department of Clinical, Neuro- and Developmental Psychology, World Health Organization Collaborating Center for Research and Dissemination of Psychological Interventions, Amsterdam Public Health Research Institute<br>Cuijpers, Pim; Vrije Universiteit Amsterdam, Department of Clinical, Neuro- and Developmental Psychology, World Health Organization Collaborating Center for Research and Dissemination of Psychological Interventions, Amsterdam Public Health Research Institute<br>Acarturk, Ceren ; Koç University, Department of Psychology<br>Akhtar, Aemal; University of New South Wales, School of Psychology<br>Alkneme, Mhd; Freie Universitat Berlin, Division of Clinical-Psychological Intervention, Department of Education and Psychology<br>Aoun, May; War Child, Research and Development Department<br>Awwad, Manar; International Medical Corps, Technical Unit<br>Bawaneh, Ahmad; International Medical Corps, Technical Unit<br>Brown, Felicity; War Child, Research and Development Department; University of Amsterdam, Amsterdam Institute of Social Science Research<br>Bryant, Richard ; University of New South Wales<br>Burchert, Sebastian; Freie Universitat Berlin, Division of Clinical-Psychological Intervention, Department of Education and Psychology<br>Carswell, Kenneth; World Health Organization, Department of Mental Health and Substance Abuse<br>Drogendijk, Annelieke; ARQ National Psychotrauma Centre, ARQ International<br>Engels, Michelle; International Federation of Red Cross and Red Crescent Societies Reference Centre for Psychosocial Support<br>Fuhr, Daniela; London School of Hygiene and Tropical Medicine, Department of Health Services Research and Policy<br>Hansen, Pernille; International Federation of Red Cross and Red Crescent Societies Reference Centre for Psychosocial Support<br>van 't Hof, Edith; World Health Organization, Department of Mental Health and Substance Abuse<br>Giardinelli, Luana; International Medical Corps, Technical Unit<br>Hemmo, Mahmoud; University Hospital Zurich, Department of Consultation-Liaison Psychiatry and Psychosomatic Medicine<br>Hessling, Jonas; Freie Universitat Berlin, Division of Clinical-Psychological Intervention, Department of Education and Psychology<br>Ilkkursun, Zeynep; Koç University, Department of Psychology |

Jordans, Mark J. D.; War Child, Research and Development Department; University of Amsterdam, Amsterdam Institute of Social Science Research
Kiselev, Nikolai; University Hospital Zurich, Department of Consultation-Liaison Psychiatry and Psychosomatic Medicine
Knaevelsrud, Christine; Freie Universitat Berlin, Division of Clinical-Psychological Intervention, Department of Education and Psychology
Kurt, Gulsah; Koç University, Department of Psychology
Martinmaki, Saara; ARQ National Psychotrauma Centre, ARQ International
McDaid, David ; The London School of Economics and Political Science, Care Policy and Evaluation Centre, Department of Health Policy
Morina, Naser; University Hospital Zurich, Department of Consultation-Liaison Psychiatry and Psychosomatic Medicine
Naser, Hadeel; International Medical Corps, Technical Unit
Park, A-La; London School of Hygiene and Tropical Medicine, Care Policy and Evaluation Centre, Department of Health Policy
Pfaltz, Monique; University Hospital Zurich, Department of Consultation-Liaison Psychiatry and Psychosomatic Medicine; Mid Sweden University, Department of Psychology and Social Work
Roberts, Bayard; London School of Hygiene and Tropical Medicine, Department of Health Services Research and Policy
Schick, Matthis; University Hospital Zurich, Department of Consultation-Liaison Psychiatry and Psychosomatic Medicine
Schnyder, Ulrich; University Hospital Zurich, Department of Consultation-Liaison Psychiatry and Psychosomatic Medicine
Spaaij, Julia; University Hospital Zurich, Department of Consultation-Liaison Psychiatry and Psychosomatic Medicine
Steen, Frederik; War Child, Research and Development Department
Taha, Karine; War Child, Research and Development Department
Uygun, Ersin; Istanbul Bilgi University, Trauma and Disaster Mental Health Master Programme
Ventevogel, Peter; United Nations High Commissioner for Refugees, Public Health
Whitney, Claire; International Medical Corps, Technical Unit
Witteveen, Anke; Vrije Universiteit Amsterdam, Department of Clinical, Neuro- and Developmental Psychology, World Health Organization Collaborating Center for Research and Dissemination of Psychological Interventions, Amsterdam Public Health Research Institute
Sijbrandij, Marit; Vrije Universiteit Amsterdam, Department of Clinical, Neuro- and Developmental Psychology, World Health Organization Collaborating Center for Research and Dissemination of Psychological Interventions, Amsterdam Public Health Research Institute

| | |
|---|---|
| <b>Primary Subject Heading</b>: | Mental health |
| Secondary Subject Heading: | Global health, Health economics |
| Keywords: | MENTAL HEALTH, Anxiety disorders < PSYCHIATRY, Depression & mood disorders < PSYCHIATRY |

SCHOLARONE™
Manuscripts

# Scalable Psychological Interventions for Syrian Refugees in Europe and the Middle East: STRENGTHS Study Protocol for a Prospective Individual Participant Data Meta-Analysis

Anne M. de Graaff (a.m.de.graaff@vu.nl),[1] Pim Cuijpers (p.cuijpers@vu.nl),[1] Ceren Acarturk (cacarturk@ku.edu.tr),[2] Aemal Akhtar (a.akhtar@unsw.edu.au),[3] Mhd Salem Alkneme (salem.alkneme@fu-berlin.de),[4] May Aoun (mayaoun71@gmail.com),[5] Manar Awwad (mawwad@InternationalMedicalCorps.org),[6] Ahmad Y. Bawaneh (abawaneh@InternationalMedicalCorps.org),[6] Felicity L. Brown (felicity.brown@warchild.nl), [5, 7] Richard Bryant (r.bryant@unsw.edu.au),[3] Sebastian Burchert (s.burchert@fu-berlin.de),[4] Kenneth Carswell (carswellk@who.int),[8] Annelieke Drogendijk (a.drogendijk@arq.org),[9] Michelle Engels (mieng@rodekors.dk),[10] Daniela C. Fuhr (Daniela.Fuhr@lshtm.ac.uk),[11] Pernille Hansen (pehan@rodekors.dk),[10] Edith van 't Hof (vanthofe@who.int),[8] Luana Giardinelli (luana.giardinelli@gmail.com),[6] Mahmoud Hemmo (Mahmoud.Hemmo@usz.ch),[12] Jonas M. Hessling (j.hessling@fu-berlin.de),[4] Zeynep Ilkkursun (zilkkursun20@ku.edu.tr),[2] Mark J.D. Jordans (mark.jordans@warchild.nl),[5, 7] Nikolai Kiselev (Nikolai.Kiselev@usz.ch),[12] Christine Knaevelsrud (christine.knaevelsrud@fu-berlin.de),[4] Gulsah Kurt (gkurt18@ku.edu.tr),[2] Saara Martinmaki (s.martinmaki@impact.arq.org),[9] David McDaid (D.Mcdaid@lse.ac.uk),[13] Naser Morina (naser.morina@usz.ch),[12] Hadeel Naser (hnaser@InternationalMedicalCorps.org),[6] A-La Park (a.park@lse.ac.uk),[13] Monique C. Pfaltz (monique.pfaltz@miun.se) [12, 14] Bayard Roberts (bayard.roberts@lshtm.ac.uk),[11] Matthis Schick (matthis.schick@usz.ch),[12] Ulrich Schnyder (ulrich.schnyder@access.uzh.ch),[12] Julia Spaaij (julia.spaaij@usz.ch),[12] Frederik Steen (frederik.steen@warchild.nl),[5] Karine Taha (karine.taha@hotmail.com),[5] Ersin Uygun (ersin.uygun@bilgi.edu.tr),[15] Peter Ventevogel (ventevog@unhcr.org),[16] Claire Whitney (cwhitney@InternationalMedicalCorps.org),[6] Anke B. Witteveen (a.b.witteveen@vu.nl),[1] Marit Sijbrandij (e.m.sijbrandij@vu.nl),[1] on behalf of the STRENGTHS consortium


For peer review only - http://bmjopen.bmj.com/site/about/guidelines.xhtml

[1] Department of Clinical, Neuro- and Developmental Psychology, WHO Collaborating Center for Research and Dissemination of Psychological Interventions, Amsterdam Public Health Research Institute, Vrije Universiteit Amsterdam, Amsterdam, The Netherlands

[2] Koc University, Department of Psychology, Istanbul, Turkey

[3] School of Psychology, University of New South Wales, Sydney, Australia

[4] Division of Clinical-Psychological Intervention, Department of Education and Psychology, Freie Universität Berlin, Berlin, Germany

[5] Research and Development Department, War Child Holland, Amsterdam, The Netherlands

[6] Technical Unit, International Medical Corps, London, UK

[7] Amsterdam Institute of Social Science Research, University of Amsterdam, Amsterdam, the Netherlands

[8] Department of Mental Health and Substance Use, World Health Organization, Geneva, Switzerland

[9] ARQ National Psychotrauma Centre, ARQ International, Diemen, The Netherlands

[10] International Federation of Red Cross and Red Crescent Societies Reference Centre for Psychosocial Support, Copenhagen, Denmark

[11] London School of Hygiene and Tropical Medicine, Department of Health Services Research and Policy, London, UK

[12] University Hospital Zurich, Department of Consultation-Liaison Psychiatry and Psychosomatic Medicine, Zurich, Switzerland

[13] Care Policy and Evaluation Centre, Department of Health Policy, London School of Economics and Political Science, London, UK

[14] Department of Psychology and Social Work, Mid Sweden University, Ostersund, Sweden

[15] Bilgi University, Trauma and Disaster Mental Health Master Programme, Istanbul, Turkey

[16] United Nations High Commissioner for Refugees, Public Health Section, Genève, Switzerland


For peer review only - http://bmjopen.bmj.com/site/about/guidelines.xhtml
For peer review only

**Corresponding author:** Anne M. de Graaff, a.m.de.graaff@vu.nl, Department of Clinical, Neuro- and Developmental Psychology, WHO Collaborating Center for Research and Dissemination of Psychological Interventions, Amsterdam Public Health Research Institute, Vrije Universiteit Amsterdam, Van der Boechorststraat 7, 1081 BT, Amsterdam, The Netherlands

**Word count**: 4069

**Abstract word count**: 290

For peer review only


For peer review only - http://bmjopen.bmj.com/site/about/guidelines.xhtml

## Abstract

### Introduction

The World Health Organization's (WHO) scalable psychological interventions, such as Problem Management Plus (PM+) and Step-by-Step are designed to be cost-effective non-specialist delivered interventions to reduce symptoms of common mental disorders, such as anxiety, depression and posttraumatic stress disorder (PTSD). The STRENGTHS consortium aims to evaluate the effectiveness, cost-effectiveness and implementation of the individual format of PM+ and its group version (gPM+), as well as of the digital Step-by-Step (SbS) intervention among Syrian refugees in seven countries in Europe and the Middle East. This is a study protocol for a prospective individual participant data (IPD) meta-analysis to evaluate 1) overall effectiveness and cost-effectiveness, and 2) treatment moderators of PM+, gPM+ and SbS with Syrian refugees.

### Methods and analysis

Five pilot RCTs and seven fully-powered RCTs conducted within STRENGTHS will be combined into one IPD meta-analytic dataset. The RCTs include Syrian refugees of 18 years and above with elevated psychological distress (Kessler Psychological Distress Scale (K10 >15)) and impaired daily functioning (WHO Disability Assessment Schedule 2.0 (WHODAS 2.0 >16)). Participants are randomized into the intervention or care as usual control group, and complete follow-up assessments at 1-week, 3-month and 12-month follow-up. Primary outcomes are symptoms of depression and anxiety (25-item Hopkins Symptom Checklist). Secondary outcomes include daily functioning (WHODAS 2.0), PTSD symptoms (PTSD Checklist for DSM-5), and self-identified problems (PSYCHLOPS). We will conduct a one-stage IPD meta-analysis using linear mixed models. Quality of evidence will be assessed using the GRADE approach, and the economic evaluation approach will be assessed using the CHEC-list.

### Ethics and dissemination


For peer review only


For peer review only - http://bmjopen.bmj.com/site/about/guidelines.xhtml

Local ethical approval has been obtained for each RCT. This IPD meta-analysis does not require ethical approval. The results of this study will be published in international peer-reviewed journals.

**Registration**

Pre-registered on OSF: DOI 10.17605/OSF.IO/WUHGF

**Strengths and limitations of this study**

- An advantage of individual participant data (IPD) meta-analysis concerns the acquired power to study overall treatment effect estimates, predictors and moderators.
- Selected randomized controlled trials are intentionally similar in study design, outcome measures and target population to allow for optimal pooling of data.
- The study sample will be large (estimated > 1000 participants) and relatively homogeneous (i.e., Syrian refugees).
- The meta-analyses will have reduced power in case individual RCTs do not meet the required sample size.

**Key words:** Mental Health; Anxiety Disorders; Depression and Mood Disorders

For peer review only


For peer review only - http://bmjopen.bmj.com/site/about/guidelines.xhtml

## Introduction

Since the outbreak of the civil war in Syria, over 13 million Syrians have been displaced. The leading destinations of Syrians displaced across borders include Turkey (3.6 million), Lebanon (910,600), and Jordan (654,700). One million fled to Europe by boat or overland [1]. Refugees often reside in unstable and insecure locations in urban areas or refugee camps [2–4], and may face uncertainty about their legal status in seeking asylum [5]. In this paper, we use 'Syrian refugees' to refer to Syrians who have been granted a refugee status and to those whose request for sanctuary still has to be processed. Due to their exposure to potentially traumatic events and other stressors before, during, and after displacement, refugees are at risk of developing mental disorders [6–9]. However, health systems are often under-resourced to respond to the mental health needs of refugee populations, especially in low- and middle-income countries, where health systems are already overburdened [10,11]. The rapidly escalated refugee crisis also poses challenges for health systems in well-resourced settings in Europe [12], where mental health services are underutilized due to barriers such as communication difficulties and fear of stigma, and access is hampered by lack of culturally appropriate mental health services and long wait lists [13–15].

The Syrian REfuGees MeNTal HealTH Care Systems (STRENGTHS) research consortium aims to evaluate the effectiveness, cost-effectiveness, and implementation of scalable psychological interventions among Syrian refugees in Europe and the Middle-East [16]. The interventions have been developed by the World Health Organization to scale up coverage for priority conditions, including depression and anxiety [17], to improve access to mental health and psychosocial support in resource-constrained settings [18]. Scalable interventions are strongly protocolized and use evidence-based psychological techniques from therapies such as cognitive behavioural therapy and interpersonal therapy. Typical features include the duration of the intervention (i.e., few sessions instead of lengthy treatment programs), the target of the intervention (i.e., transdiagnostic instead of focused on a single disorder), and the delivery agent (i.e., task-shifting to non-specialist providers) [17,19].


For peer review only - http://bmjopen.bmj.com/site/about/guidelines.xhtml

One of the interventions that is currently being tested by the STRENGTHS consortium is Problem Management Plus (PM+), which can be delivered by trained and supervised non-specialists to reduce psychological distress in communities affected by adversity [20]. The first randomized controlled trials (RCTs) on PM+ have been conducted in communities affected by violence in Pakistan and Kenya [21–23], and in a disaster-prone community in Nepal [24]. PM+ for individuals led to improvements in anxiety, depression, posttraumatic stress disorder (PTSD), psychosocial functioning, and self-identified problems three months after the intervention [21,23]. Although PM+ was more effective in reducing symptoms of common mental disorders, it was also costlier compared to CAU alone [25]. PM+ delivered in groups (gPM+) to women in Pakistan was effective in reducing symptoms of anxiety, depression, and self-identified problems, but not for symptoms of PTSD [22]. In Nepal, gPM+ for men and women was also associated with improvements in psychological distress, depression, and "heart-mind" problems, but not for psychosocial functioning and symptoms of PTSD [24]. Another non-specialist delivered intervention based on PM+ that is currently being tested by the STRENGTHS consortium is the digital Step-by-Step (SbS) intervention [26]. SbS is a minimally guided e-mental health intervention with 'e-helper' support. Pilot studies on SbS in Lebanon, including Syrian participants, found that the intervention was acceptable [27], and likely effective in improving psychosocial outcomes [28].

Pilot studies among Syrian refugees carried out by the STRENGTHS consortium indicated that PM+ delivered by peers is an acceptable and feasible intervention, and likely effective in reducing psychological distress [29–32]. Currently, fully-powered (i.e., based on a priori power analysis) pragmatic RCTs among adult Syrian refugees in Europe and the Middle East are being conducted to test the effectiveness of PM+ in individual format in Switzerland and the Netherlands [33], PM+ in group format (gPM+) in Jordan [34] and Turkey [35], and the digital Step-by-Step (SbS) intervention in Germany, Sweden and Egypt [16]. The interventions are compared with care as usual (CAU). CAU refers to all (mental) health services available to refugees in the setting where the pragmatic trial is


For peer review only - http://bmjopen.bmj.com/site/about/guidelines.xhtml

conducted [36]. Although study sites differ with regard to the availability of services, barriers to accessing care have been identified across host countries [13,15,37].

Initial results of PM+/gPM+ delivered by trained peer-refugees are promising when looking at overall effects. However, individual differences in symptom severity, socio-demographic background, comorbidities, living circumstances, and life events may result in individual differences in treatment response. Single trials do not have sufficient power to detect genuine interaction effects between participant characteristics and treatment outcome [38]. To determine which individuals are more or less likely to benefit from these scalable psychological interventions, an individual participant data (IPD) meta-analytic approach can be used [39,40]. This approach does not rely on aggregate (study-level) data used in conventional meta-analysis techniques, but uses the original data of each individual participant (participant-level) from eligible RCTs. The IPD meta-analysis approach offers greater statistical power and precision to better understand the predictive nature of individual characteristics on treatment outcome [38,41]. Furthermore, it also allows us to get a better estimate of overall effectiveness and cost-effectiveness, by pooling several large data sets together.

This protocol outlines a planned IPD meta-analysis on combined datasets from the STRENGTHS project, aiming to examine 1) the overall effectiveness and cost-effectiveness of scalable psychological interventions (i.e., PM+, gPM+, SbS) as compared to CAU alone on improving depression and anxiety (primary outcomes), PTSD symptoms, psychosocial functioning, and self-identified problems (secondary outcomes) among Syrian refugees, and 2) sociodemographic, migratory, and clinical predictors and moderators of treatment outcome (i.e., treatment-covariate interactions).

## Methods

This protocol was pre-registered in Open Science Framework (DOI 10.17605/OSF.IO/WUHGF), and all individual RCTs were prospectively registered online (see Table 1). The PRISMA-P checklist [42]



For peer review only - http://bmjopen.bmj.com/site/about/guidelines.xhtml

is appended. The IPD meta-analysis, including merging all datasets and data analyses will be conducted between January 2022 and July 2022.

[INSERT TABLE 1 ABOUT HERE]

**Patient and public involvement**

The STRENGTHS project installed an independent external expert board (Project Advisory Board) including mental health professionals from Syria. The expert board is regularly consulted on the quality of the results and output of STRENGTHS, as well as on the dissemination and exploitation of the results. Prior to conducting the RCTs, qualitative studies among Syrian refugees in each participating country were conducted to inform intervention development, study design and recruitment strategies.

**Inclusion of datasets**

We will include all STRENGTHS randomized controlled trials (RCTs) on the effectiveness of PM+ for individuals (Switzerland and the Netherlands), PM+ for groups (gPM+) (Jordan and Turkey), and SbS (Sweden, Germany and Egypt). These RCTs involve a relatively homogeneous population, and have similar study design and outcomes measures. The trials are presented in Table 1.

**Participants and procedure**

In the trials, Arabic-speaking Syrian refugees of 18 years and above are included if they report psychological distress (Kessler Psychological Distress Scale (K10 >15)) [43] and impaired daily functioning (WHO Disability Assessment Schedule 2.0 (WHODAS 2.0 >16)) [44]. The K10 and WHODAS have been found to be valid screening instruments among refugee populations [45–47].

For peer review only



For peer review only - http://bmjopen.bmj.com/site/about/guidelines.xhtml

The fully-powered RCT on PM+ in Switzerland also includes Arabic-speaking refugees from other countries. In Jordan, an additional criterion is having a child/dependent between the ages 10-16 years.

Across trials, participants are excluded if there are signs of imminent suicide risk (PM+ manual questionnaire) [48]. Furthermore, all trials except those on SbS in Sweden, Germany and Egypt exclude participants with acute medical conditions, expressed acute needs or protection risks, and indications of severe mental disorders (e.g., psychotic disorders or substance-dependence) or cognitive impairment (e.g., severe intellectual disability or dementia) (PM+ manual observation checklist) [48]. The fully-powered PM+ trial in the Netherlands also excludes participants currently receiving specialized psychological treatment. Other exclusion criteria in Switzerland include being under guardianship and inability to follow study procedures.

In line with the main aim of the STRENGTHS project, the IPD meta-analysis will include only Syrian refugees. The RCTs in Turkey, Switzerland and the Netherlands are conducted in a community setting, while the RCTs in Jordan are conducted in a refugee camp. At this moment, all pilot trials have been completed, with sample sizes ranging from 46 to 64 (total $N$=302). Recruitment has been completed for the fully-powered trials in Turkey ($N$=369), Jordan ($N$=410), and Egypt ($N$±538). All trials randomise individuals 1:1 to the intervention (PM+/CAU, gPM+/CAU, or SbS/CAU) or control group (CAU only). Assessments are conducted at baseline, one-week after the intervention, three months after the intervention, and 12 months after baseline. The 12-month assessment was not part of the pilot RCTs. The pilot RCT in Jordan only included a baseline and one-week follow-up assessment. Treatment conditions are further explained below.

**The interventions**

The manuals for all interventions were adapted by the STRENGTHS consortium according to a framework for the cultural adaptation of psychological interventions [49], and materials to provide training for trainers and helpers/facilitators were developed [50,51].


For peer review only - http://bmjopen.bmj.com/site/about/guidelines.xhtml

**Problem Management Plus (PM+) for individuals and groups**

PM+ is a brief, transdiagnostic psychological intervention developed to reduce symptoms of common mental disorders such as depression, anxiety, and PTSD [20]. PM+ includes five weekly sessions provided by trained non-specialist helpers. During this 5-week intervention, the participant learns skills in arousal reduction, problem-solving, behavioural activation and accessing social support. These strategies include a relaxation exercise using slow breathing (session 1), a 7-step plan to manage practical problems (session 2), behavioural activation by re-engaging with pleasant and task-oriented activities (session 3), and a strategy to strengthen social support (session 4). Homework is scheduled between sessions and strategies are reviewed in each subsequent session. Session 5 focuses on relapse prevention. The individual PM+ intervention consists of five 90-minute sessions. The group PM+ intervention consists of five 2-hour sessions in groups of 6-10 participants of the same gender led by two gender-matched group facilitators [20,51].

PM+ is delivered by Arabic-speaking peer-refugees who have completed high school, and have a background in education, social work, health care, or another related field. In Jordan, group facilitators were members of the host community. Helpers/group facilitators in Switzerland, the Netherlands and Turkey also needed to be proficient in English or the local language (i.e., German, Dutch or Turkish). Helpers/group facilitators received an eight-day training of helpers (ToH) on common mental disorders, basic counselling skills, delivery of intervention strategies and self-care. The ToH was followed by practice cases. Helpers/group facilitators receive weekly supervision by trained PM+ trainers and supervisors throughout the trial. PM+ trainers/supervisors are mental health care professionals who received a five-day training of trainers (ToT) on elements covered in the ToH, as well as on training and supervision skills. They receive monthly supervision by a PM+ master trainer [20].

For peer review only


For peer review only - http://bmjopen.bmj.com/site/about/guidelines.xhtml

### Step-by-Step (SbS)

The SbS intervention is a 5-session, digital self-help intervention for iOS, Android and Web platforms, aimed at reducing depressive symptoms. The strategies that are introduced in this 5-week intervention include psychoeducation and trying small and pleasant activities (session 1), behavioural activation (session 2), stress management (session 3), accessing social support (session 4), and positive self-verbalization and relapse prevention (session 5). Homework practice is scheduled between sessions. WHO removed problem-solving from SbS, as it was found to be too complex to implement in a digital application with or without helper support [26]. The intervention uses an illustrated, narrative story of a fictional character that seeks help for depression from a professional. The narrative provides educative information on the strategies, supplemented by interactive exercises (e.g., activity scheduling). Sessions last 30 minutes, and involve an introduction part, practice part, and reinforcement part with optional contact-on-demand to non-specialist, Arabic-speaking "e-helpers" available through a text and audio messaging system. E-helpers are university students trained and supervised by mental health professionals on basic helping skills, providing basic support, and on the identification, assessment and management of risk and (serious) adverse events.

### Care as usual (CAU)

The identified studies compared PM+, gPM+ or SbS in addition to CAU, to CAU alone. CAU includes all (mental) health services available to refugees across settings. In high-income countries, this includes referral to (specialized) mental health services through primary care practitioners (e.g., general practitioner). In Turkey, refugees have free access to mental health services in public hospitals and migrant health centres. Refugees residing in refugee camps in Jordan can access services inside the camp provided by national and international non-governmental organisations. However, across sites, access to mental health services is hampered by numerous barriers, such as the lack of Arabic-speaking health providers in European countries, and overburdened health systems of neighbouring


For peer review only - http://bmjopen.bmj.com/site/about/guidelines.xhtml
For peer review only

countries hosting most refugees [13,14]. All study participants received information about available mental health services.

**Outcomes**

Similar to the individual RCTs, the primary outcomes in this IPD meta-analysis will be symptoms of depression and anxiety at 3-month follow-up, measured by the subscales of the Hopkins Symptom Checklist (HSCL-25) [52–54]. Secondary outcomes include daily functioning (WHODAS 2.0), PTSD symptoms (PTSD Checklist for DSM-5; PCL-5) [55], and self-identified problems (PSYCHLOPS) [56]. Measures were selected based on their acceptable validity and reliability in refugee and Arabic-speaking groups, as well as their availability in the Arabic language and prior use with Syrian refugees [45,46,53,54,57,58]. We will also examine these outcomes at the 1-week post-assessment and 12-month follow-up.

To calculate cost-effectiveness, in addition to documented resources required for intervention delivery, we will examine the pooled changes in resource use associated with use of health-related services, and time out of usual activities that have been collected using an adapted version of the Client Service Receipt Inventory (CSRI) [59]. All measures were pilot tested using cognitive interviews with members of the target community [34].

**Study-level variables**

We will obtain study-level data for each trial including country and setting in which the study was conducted, and the type of intervention tested (e.g., PM+, gPM+, SbS). We will thus test overall effectiveness for all interventions together as well as the differential effect of each type of intervention.

**Individual-level variables**


For peer review only - http://bmjopen.bmj.com/site/about/guidelines.xhtml

We will extract individual-level data from each dataset to test a range of variables that may moderate the effects of the interventions on primary and secondary outcomes, to determine differential treatment effects based on participant characteristics. We will gather and synthesize all socio-demographic variables (i.e., age, gender, education level, work status, marital status), clinical characteristics (i.e., symptoms of depression, anxiety and PTSD, daily functioning, the exposure and type of traumatic events, post-migration stressors), and migration variables (i.e., time spent in host country, refugee status).

## IPD collection and aggregation

All research teams have agreed to contribute RCT data to these IPD meta-analyses. De-identified primary datasets will be shared using secure password protected data links. Data will be stored in a secure cloud service (Surfdrive) developed for the Dutch education and research community, which can only be accessed by the IPD meta-analysis research team. Data transfer, storage and handling will follow the EU General Data Protection Regulations (GDPR).

Data accuracy of the primary datasets will be examined to explore if it matches data reported in published reports. We will specifically check frequencies of socio-demographic variables (e.g., age, gender, marital status, education level) and descriptive statistics (e.g., mean scores) of continuous scales. If discrepancies between the primary dataset and published report arise, clarification will be sought from authors. After the primary datasets are checked for accuracy, all eligible datasets will be merged into the IPD meta-analytic dataset.

## Quality of evidence

We will assess the quality of evidence by applying the Grading of Recommendations, Assessment, Development and Evaluation (GRADE) approach [60]. The GRADE approach is a system for rating the quality (i.e., high, moderate, low, and very low) applied to a body of evidence, and not to the

For peer review only


For peer review only - http://bmjopen.bmj.com/site/about/guidelines.xhtml

individual studies. GRADE offers a transparent and structured process for developing and presenting evidence summaries and for carrying out the steps involved in developing recommendations. The GRADE approach consists of rating eight criteria, including five that may lead to rating down the quality of evidence (i.e., risk of bias, inconsistency, indirectness, imprecision, publication bias) and three that may lead to rating up (i.e., large effect, dose-response gradient, minimal influence of residual plausible confounding). Results will be summarized in a GRADE evidence profile [61]. For the economic analysis, we will assess the economic evaluation approach using the Consensus Health Economic Criteria list (CHEC-list) [62].

**The IPD meta-analysis**

All analyses will be conducted in R version 4.0.3 [63] using the packages metafor [64] and lme4 [65]. The economic evaluation will be conducted in STATA statistical software [66]. We will use a 'one-stage IPD meta-analytic' approach, in which the IPD of all identified studies will be merged into one large dataset. The resulting dataset is analysed in one step, as if all participants, clustered within studies, belonged to a single trial. This approach is preferred over the 'two-stage IPD meta-analytic' approach, in which aggregated summary points are pooled in an appropriate meta-analysis model. The one-stage approach allows for more advanced modelling of the moderators, can adopt more appropriate likelihood functions and has fewer assumptions [67].

The IPD meta-analysis will be conducted according to the intention-to-treat principle (i.e., all randomized participants will be included in the analyses). Missing values for outcome data will be estimated under the missing-at-random assumption, using multiple imputation (100 imputations) [68,69]. To estimate the missing values, valid predictor variables such as individual clinical and socio-demographic variables will be used (e.g., distress levels at baseline, age, gender). Sensitivity analyses will be conducted on complete cases only (i.e., with outcome data) to test for differences between imputed and completed values, and with completers of the interventions only.



For peer review only - http://bmjopen.bmj.com/site/about/guidelines.xhtml

To examine the effects of PM+, gPM+ and SbS on primary and secondary outcomes at 3-month follow-up, we will perform a mixed effect linear regression model with random intercepts with each trial having a random effect and a fixed effect for the intervention and the outcome measures. The severity of depressive and anxiety symptoms, PTSD symptoms, daily functioning, and self-identified problems, and costs of care at the 3-month follow-up will be used as dependent variables, and condition (e.g., treatment versus control) will be the independent variable while adjusting for baseline scores. The standardized $\beta$ coefficient indicates how many standard deviations (*SD*) the dependent variables (i.e., anxiety, depression, PTSD, daily functioning, self-identified problems, and costs of care) change per *SD* increase in the independent variable. Thus, the larger the $\beta$ coefficient, the greater the effect of the independent variable on the respective dependent variable, with no association among the variables if $\beta$ is 0.

To ensure robustness of our findings, the analysis of the primary outcomes will be repeated using the 'two-stage IPD meta-analytic approach'. In a two-stage approach, Cohen's *d* will be calculated for all outcomes for every single trial separately (step 1), and will then be combined to calculate the pooled effect sizes using the random-effects model (step 2) [70].

For the economic analysis incremental cost-effectiveness ratios comparing changes in effect and costs between the intervention group and controls on the primary outcome will also be estimated. Uncertainty in mean differences in intervention resource use as well as the use of health and other services between baseline and 3-month follow-up between the two groups will be accounted for using bias-corrected and accelerated bootstrapping. Purchasing power parity adjusted unit costs expressed in international dollars will be applied to resource use. Discounting will not be applied given the short duration of the study. Cost-effectiveness acceptability curves (CEACs) will be generated to showing the likelihood that intervention is cost-effective at different willingness-to-pay thresholds.

**Participant- and study-level moderators**


For peer review only - http://bmjopen.bmj.com/site/about/guidelines.xhtml
For peer review only

We will test whether socio-demographic, clinical and migration variables moderate the effects of psychological interventions among Syrian refugees at 3-month follow-up. To examine the effects of potential moderators, we will add an interaction term between each moderator variable and condition on depressive and anxiety symptoms into the mixed-effects linear/logistic regression models. Each potential moderator variable will be added into separate bivariate models.

**Heterogeneity**

We will calculate the $I^2$ statistic to assess statistical heterogeneity indicated in percentages, with 0% indicating no heterogeneity, 25% low heterogeneity, 50% moderate heterogeneity, and 75% high heterogeneity [71]. We will also calculate 95% Confidence Intervals (CI) around $I^2$ using the non-central chi-squared-based approach to provide the full magnitude of heterogeneity [72].

**Small sample bias**

The present study protocol describes a prospective IPD meta-analysis of RCTs conducted within the STRENGTHS project. We will examine small sample bias by inspecting the funnel plot in which sample size is plotted against the effect estimates of the primary outcome measures. Egger's test of the intercept will be carried out to inspect the degree of asymmetry of the plot [73]. Lastly, we will estimate the effect size adjusted for bias using the trim and fill procedure [74].

**Ethics and dissemination**

Local ethical approval has been obtained for each RCT. This IPD meta-analysis does not require ethical approval. The results of this study will be published in international peer-reviewed journals, and will be made publicly available through the STRENGTHS website (www.STRENGTHS-project.eu).


For peer review only - http://bmjopen.bmj.com/site/about/guidelines.xhtml

## Discussion

Scalable psychological interventions such as PM+, gPM+ and SbS for communities affected by adversity have been found to be effective in various RCTs [21–24]. Pilot studies on PM+ adapted for Syrian refugees indicated the intervention's feasibility and likely effectiveness when delivered by peer-refugees to individuals [30,31] or groups [29,32]. The STRENGTHS consortium tests the effectiveness of different types of scalable interventions (i.e., PM+, gPM+ and SbS) among Syrian refugees across different settings (e.g., refugee camp, community setting). The present paper describes the procedures of an IPD meta-analysis aimed at examining the overall effectiveness and cost-effectiveness of the interventions compared with CAU alone, and at examining individual participant differences in treatment response.

This study protocol has several strengths. An essential advantage of IPD meta-analytic approaches over using conventional meta-analytic approaches concerns the acquired power to study overall effect estimates, predictors and moderators, by synthesizing the original data from all individual RCTs into one large dataset. Single RCTs frequently do not have enough statistical power to investigate moderators. In conventional meta-analysis, moderation analyses are commonly done using subgroup analyses of aggregate data (e.g., effect sizes), but this limits statistical power and accuracy due to a loss of degrees of freedom and variability in the moderator of interest [67]. IPD meta-analysis also allows us to investigate outcome variables that primary studies have not reported. Another strength of our protocol concerns the availability of study trials with similar population, study design and outcome variables. This enables us to determine who benefits more or less from scalable psychological interventions using a meta-analytic approach that is least biased and most reliable for addressing this question.

This study has important limitations that have to be taken into account when interpreting the results. First, overall statistical power may be reduced in case individual RCTs do not meet the required sample size based on a priori power calculation. Second, study participants reside in different


For peer review only - http://bmjopen.bmj.com/site/about/guidelines.xhtml

settings, including refugee camps with limited access to mental health care, and community settings in high-income countries where specialist services are widely available although not always easily accessible to refugees. Although we will register the CAU delivered at each site using the CSRI measure, it cannot be precluded that differences in CAU may affect the results. Furthermore, migratory patterns as well as the ongoing stressors might differ considerably across settings [1,75]. Third, the use of different recruitment strategies across and within trials may have affected sample composition and treatment effects [76]. Fear of stigma has been found to be one of the reasons refugees may not access mental health services [15]. For trials where participants are mainly self-selected (e.g., through social media campaigns in Germany or the Netherlands), participants may also hold more positive attitudes towards mental health services. Participants in Jordan, however, were actively recruited door-to-door by members of the research team. Thus, study samples across sites may systematically differ from each other regarding their motivation to engage in mental health services, which in turn may affect the interpretation of the IPD meta-analysis results [76]. Furthermore, the results may be influenced by response-shifts at follow-up on self-report measures such as the HSCL-25 (e.g., lack of unidimensionality and temporal invariance) [77], especially when response-shifts differ between treatment and control groups [e.g., 78].

This IPD meta-analysis protocol will extend our current knowledge on the effectiveness of scalable psychological interventions for refugees and other populations affect by adversity. It will allow for more accurate estimations of treatment effects, cost-effectiveness, and the exploration of important predictors and treatment moderators of scalable interventions for sub-groups of refugees in Europe and the Middle East. The results of this study will be disseminated through publication in peer-reviewed journals.

**Authors' contributions**


For peer review only - http://bmjopen.bmj.com/site/about/guidelines.xhtml

AG drafted the study protocol. AG, PC, CA, AA, MSA, MAw, AB, RB, SB, ME, DF, LG, MH, JH, ZI, NK, CK, GK, DM, NM, HN, AP, MP, RB, MSch, US, JS, EU, CW, AW and MSij contributed to the original data acquisition. DM and AP provided input for the economic analysis. AG, PC, CA, AA, MSA, MAo, MAw, AB, FB, RB, SB, KC, AD, ME, DF, PH, EH, LG, MH, JH, ZI, MJ, NK, CK, GK, SM, DM, NM, HN, AP, MP, BR, MSch, US, FS, JS, KT, EU, PV, CW, AW and MSij all provided input for the study design. AG, PC, CA, AA, MSA, MAo, MAw, AB, FB, RB, SB, KC, AD, ME, DF, PH, EH, LG, MH, JH, ZI, MJ, NK, CK, GK, SM, DM, NM, HN, AP, MP, BR, MSch, US, FS, JS, KT, EU, PV, CW, AW and MSij all read, commented and approved the final protocol. MSij is the guarantor of the protocol. This protocol is written on behalf of the STRENGTHS consortium.

**Funding**

The STRENGTHS project is funded under Horizon 2020 – the Framework Programme for Research and Innovation (2014-2020). The authors alone are responsible for the views expressed in this article and they do not necessarily represent the views, decisions or policies of the institutions with which they are affiliated. The European Community is not liable for any use that may be made of the information contained therein.

**Competing interests**

David McDaid: Payment to institution for advice (not related to the interventions evaluated in STRENGTHS) given to the British Association for Counselling and Psychotherapy in January 2022.

**Patient consent for publication**

Not required.

**Data**

The Vrije Universiteit Amsterdam (VU) will keep a central data repository of all data collected in the STRENGTHS project. The data will be available upon reasonable request to the STRENGTHS



For peer review only - http://bmjopen.bmj.com/site/about/guidelines.xhtml

consortium. Data access might not be granted to third parties when this would interfere with relevant data protection and legislation in the countries participating in this project and any applicable EU legislation regarding data protection. Interested researchers can contact Dr Marit Sijbrandij at e.m.sijbrandij@vu.nl to initiate the process.

## ORCID IDs

Anne M. de Graaff https://orcid.org/0000-0001-6686-4432

Pim Cuijpers https://orcid.org/0000-0001-5497-2743

Ceren Acarturk https://orcid.org/0000-0001-7093-1554

Aemal Akhtar https://orcid.org/0000-0002-8510-3636

Mhd Salem Alkneme https://orcid.org/0000-0002-4593-6705

Felicity Brown: https://orcid.org/0000-0001-6800-1657

Richard Bryant https://orcid.org/0000-0002-9607-819X

Sebastian Burchert https://orcid.org/0000-0003-3126-5485

Kenneth Carswell: https://orcid.org/0000-0001-5344-5802

Michelle Engels https://orcid.org/0000-0002-3782-1609

Daniela C. Fuhr https://orcid.org/0000-0001-9020-4629

Pernille Hansen https://orcid.org/0000-0002-4782-458X

Mahmoud Hemmo https://orcid.org/0000-0002-2958-0702

Edith van ‘t Hof https://orcid.org/0000-0002-5856-3573

Zeynep Ilkkursun https://orcid.org/0000-0001-6691-1809

Mark J.D. Jordans https://orcid.org/0000-0001-5925-8039

Nikolai Kiselev https://orcid.org/0000-0003-0617-0353

Christine Knaevelsrud https://orcid.org/0000-0003-1342-7006

Saara E. Martinmäki https://orcid.org/0000-0003-0230-4641

For peer review only


For peer review only - http://bmjopen.bmj.com/site/about/guidelines.xhtml

David McDaid https://orcid.org/0000-0003-0744-2664

Naser Morina https://orcid.org/0000-0002-6470-4408

A-La Park https://orcid.org/0000-0002-4704-4874

Monique Pfaltz https://orcid.org/0000-0002-7762-6226

Ulrich Schnyder https://orcid.org/0000-0003-3556-7990

Marit Sijbrandij https://orcid.org/0000-0001-5430-9810

Peter Ventevogel https://orcid.org/0000-0002-3567-8861

Claire Whitney https://orcid.org/0000-0002-1100-5363

Anke Witteveen https://orcid.org/0000-0002-9636-7522

For peer review only


For peer review only - http://bmjopen.bmj.com/site/about/guidelines.xhtml

## Literature

1. UNHCR. Trends at a glance: Global trends forced displacement in 2019. 2020. Available from: https://www.unhcr.org/5ee200e37.pdf

2. Silove D, Ventevogel P, Rees S. The contemporary refugee crisis: an overview of mental health challenges. World Psychiatry. 2017;16:130–9.

3. Alshawawreh LA, Smith RS, Wood JB. Assessing the Sheltering Response in the Middle East: Studying Syrian Camps in Jordan. Int Sch Sci Res Innov. 2017;11:1751–7. Available from: https://reliefweb.int/sites/reliefweb.int/files/resources/01 Azraq Fact Sheet January 2021.pdf

4. UNHCR. Zaatari Camp Fact Sheet. 2021. Available from: https://reliefweb.int/sites/reliefweb.int/files/resources/01 Zaatari Fact Sheet January 2021.pdf

5. Cange CW, Brunell C, Acarturk C, Fouad FM. Considering chronic uncertainty among Syrian refugees resettling in Europe. Lancet Public Heal. 2019;4:e14. Available from: http://dx.doi.org/10.1016/S2468-2667(18)30261-5

6. Steel Z, Chey T, Silove D, Marnane C, Bryant RA, Van Ommeren M. Association of torture and other potentially traumatic events with mental health outcomes among populations exposed to mass conflict and displacement: A systematic review and meta-analysis. JAMA - J Am Med Assoc. 2009;302:537–49.

7. Schick M, Zumwald A, Knopfli B, Nickerson A, Bryant RA, Schnyder U, et al. Challenging future, challenging past: The relationship of social integration and psychological impairment in traumatized refugees. Eur J Psychotraumatol. 2016;7:1–9.

8. UNHCR. Global Trends Forced Displacement in 2020. 2021 [cited 2021 Sep 27]. Available from: https://www.unhcr.org/flagship-reports/globaltrends/

9. Fazel M, Reed R V, Panter-Brick C, Stein A. Mental health of displaced and refugee children resettled in high-income countries: Risk and protective factors. Lancet. 2012;379:266–82. Available from: mina.fazel@psych.ox.ac.uk



For peer review only - http://bmjopen.bmj.com/site/about/guidelines.xhtml

10. WHO. Building Back Better: Sustainable Mental Health Car after Emergencies. World Heal. Organ. 2013.

11. Fuhr DC, Acarturk C, McGrath M, Ilkkursun Z, Sondorp E, Sijbrandij M, et al. Treatment gap and mental health service use among Syrian refugees in Sultanbeyli, Istanbul: a cross-sectional survey. Epidemiol Psychiatr Sci. 2020;29:e70.

12. Lebano A, Hamed S, Bradby H, Gil-Salmerón A, Durá-Ferrandis E, Garcés-Ferrer J, et al. Migrants' and refugees' health status and healthcare in Europe: A scoping literature review. BMC Public Health. BMC Public Health; 2020;20.

13. Satinsky E, Fuhr DC, Woodward A, Sondorp E, Roberts B. Mental health care utilisation and access among refugees and asylum seekers in Europe: A systematic review. Health Policy (New York). Elsevier Ireland Ltd; 2019;123:851–63. Available from: https://doi.org/10.1016/j.healthpol.2019.02.007

14. Kiselev N, Pfaltz M, Haas F, Schick M, Kappen M, Sijbrandij M, et al. Structural and socio-cultural barriers to accessing mental healthcare among Syrian refugees and asylum seekers in Switzerland. Eur J Psychotraumatol. Taylor & Francis; 2020;11. Available from: https://doi.org/10.1080/20008198.2020.1717825

15. Due C, Green E, Ziersch A. Psychological trauma and access to primary healthcare for people from refugee and asylum-seeker backgrounds: A mixed methods systematic review. Int J Ment Health Syst. BioMed Central; 2020;14:1–18. Available from: https://doi.org/10.1186/s13033-020-00404-4

16. Sijbrandij M, Acarturk C, Bird M, Bryant RA, Burchert S, Carswell K, et al. Strengthening mental health care systems for Syrian refugees in Europe and the Middle East: integrating scalable psychological interventions in eight countries. Eur J Psychotraumatol. Taylor & Francis; 2017;8:1388102. Available from: https://www.tandfonline.com/doi/full/10.1080/20008198.2017.1388102

For peer review only


For peer review only - http://bmjopen.bmj.com/site/about/guidelines.xhtml

17. WHO. Scalable psychological interventions for people in communities affected by adversity. A new area of mental health and psychosocial work at WHO. 2017.

18. WHO. Mental Health Gap Action Programme: scaling up care for mental, neurological and substance use disorders. mhGAP Ment. Heal. Gap Action Program. 2008. Available from: http://www.who.int/mental_health/evidence/mhGAP/en/

19. Singla DR, Kohrt BA, Murray LK, Anand A, Chorpita BF, Patel V. Psychological treatment for the world: Lessons from low- and middle-income countries. Annu Rev Clin Psychol. 2017;49:149–81.

20. Dawson KS, Bryant RA, Harper M, Kuowei Tay A, Rahman A, Schafer A, et al. Problem Management Plus (PM+): a WHO transdiagnostic psychological intervention for common mental health problems. World Psychiatry. 2015;14:354–7.

21. Rahman A, Hamdani SU, Awan NR, Bryant RA, Dawson KS, Khan MF, et al. Effect of a multicomponent behavioral intervention in adults ipairment by psychological distress in a conflict-affected area of Pakistan. A randomized controlled trial. JAMA. 2016;316:2609–17.

22. Rahman A, Khan MN, Hamdani SU, Chiumento A, Akhtar P, Nazir H, et al. Effectiveness of a brief group psychological intervention for women in a post-conflict setting in Pakistan: a single-blind, cluster, randomised controlled trial. Lancet. Elsevier Ltd; 2019; Available from: https://linkinghub.elsevier.com/retrieve/pii/S0140673618323432

23. Bryant RA, Schafer A, Dawson KS, Anjuri D, Mulili C, Ndogoni L, et al. Effectiveness of a brief behavioural intervention on psychological distress among women with a history of gender-based violence in urban Kenya: A randomised clinical trial. PLoS Med. 2017;14:e1002371.

24. Jordans MJD, Kohrt BA, Sangraula M, Turner EL, Wang X, Shrestha P, et al. Effectiveness of Group Problem Management Plus, a brief psychological intervention for adults affected by humanitarian disasters in Nepal: A cluster randomized controlled trial. PLoS Med. 2021;18:1–22.

25. Hamdani SU, Huma ZE, Rahman A, Wang D, Chen T, Van Ommeren M, et al. Cost-

For peer review only



For peer review only - http://bmjopen.bmj.com/site/about/guidelines.xhtml

effectiveness of WHO Problem Management plus for adults with mood and anxiety disorders in a post-conflict area of Pakistan: Randomised controlled trial. Br J Psychiatry. 2020;217:623–9.

26. Carswell K, Harper-Shehadeh M, Watts S, van't Hof E, Abi Ramia J, Heim E, et al. Step-by-Step: a new WHO digital mental health intervention for depression. mHealth. 2018;4:34–34.

27. Harper Shehadeh MJ, Abi Ramia J, Cuijpers P, El Chammay R, Heim E, Kheir W, et al. Step-by-Step, an E-Mental Health Intervention for Depression: A Mixed Methods Pilot Study From Lebanon. Front Psychiatry. 2020;10:1–11.

28. Heim E, Ramia JA, Hana RA, Burchert S, Carswell K, Cornelisz I, et al. Step-by-step: Feasibility randomised controlled trial of a mobile-based intervention for depression among populations affected by adversity in Lebanon. Internet Interv. Elsevier B.V.; 2021;24:100380. Available from: https://doi.org/10.1016/j.invent.2021.100380

29. de Graaff AM, Cuijpers P, McDaid D, Park A, Woodward A, Bryant RA, et al. Peer-provided Problem Management Plus (PM+) for adult Syrian refugees; a pilot randomized controlled trial on effectiveness and cost-effectiveness. Epidemiol Psychiatr Sci. 2020;29:1–24.

30. Akhtar A, Giardinelli L, Bawaneh A, Awwad M, Naser H, Whitney C, et al. Group problem management plus (gPM+) in the treatment of common mental disorders in Syrian refugees in a Jordanian camp: Study protocol for a randomized controlled trial. BMC Public Health. BMC Public Health; 2020;20:1–8.

31. Acarturk C, Uygun E, Ilkkursun Z, Yurtbakan T, Adam-Troian J, Senay I, et al. Group Problem Management Plus (PM+) to decrease psychological distress among Syrian refugees in Turkey: A pilot randomised controlled trial. BMC Psychiatry. 2022;22:8.

32. Spaaij J, Kiselev N, Berger C, Bryant R, Cuijpers P, de Graaff A, et al. Feasibility and acceptability of Problem Management Plus (PM+) among Syrian refugees in Switzerland: a pilot randomized controlled trial.

33. Akhtar A, Giardinelli L, Bawaneh A, Awwad M, Al-Hayek H, Whitney C, et al. Feasibility trial

For peer review only


For peer review only - http://bmjopen.bmj.com/site/about/guidelines.xhtml

of a scalable transdiagnostic group psychological intervention for Syrians residing in a refugee camp. European Journal of Psychotraumatology; 2021;12:1932295. Available from: https://doi.org/10.1080/20008198.2021.1932295

34. de Graaff A, Cuijpers P, Acarturk C, Bryant R, Burchert S, Fuhr D, et al. Effectiveness of a peer-refugee delivered psychological intervention to reduce psychological distress among adult Syrian refugees in the Netherlands: study protocol. European Journal of Psychotraumatology; 2020;11:1694347.

35. Uygun E, Ilkkursun Z, Sijbrandij M, Aker AT, Bryant R, Cuijpers P, et al. Protocol for a randomized controlled trial: Peer-to-peer Group Problem Management plus (PM+) for adult Syrian refugees in Turkey. Trials. Trials; 2020;21:1–9.

36. Cuijpers P, Quero S, Papola D, Cristea IA, Karyotaki E. Care-as-usual control groups across different settings in randomized trials on psychotherapy for adult depression: a meta-analysis. Psychol Med. 2021;51:634–44.

37. Cratsley K, Brooks MA, Mackey TK. Refugee Mental Health, Global Health Policy, and the Syrian Crisis. Front Public Heal. 2021;9:1–20.

38. Riley RD, Debray TPA, Fisher D, Hattle M, Marlin N, Hoogland J, et al. Individual participant data meta-analysis to examine interactions between treatment effect and participant-level covariates: Statistical recommendations for conduct and planning. Stat Med. 2020;39:2115–37.

39. Berlin JA, Santanna J, Schmid CH, Szczech LA, Feldman HI. Individual patient- versus group-level data meta-regressions for the investigation of treatment effect modifiers: Ecological bias rears its ugly head. Stat Med. 2002;21:371–87.

40. Fisher DJ, Carpenter JR, Morris TP, Freeman SC, Tierney JF. Meta-analytical methods to identify who benefits most from treatments: Daft, deluded, or deft approach? BMJ. 2017;356:1–6.

41. Stewart L, Parmar M. Meta-analysis pof the literature or of individual patient data: is there a difference? Lancet. 1993;341:418–22.



For peer review only - http://bmjopen.bmj.com/site/about/guidelines.xhtml

42. Moher D, Shamseer L, Clarke M, Ghersi D, Liberati A, Petticrew M, et al. Preferred reporting items for systematic review and meta-analysis protocols (PRISMA-P) 2015 statement. Syst Rev. 2015;4:1.

43. Kessler RC, Andrews G, Colpe LJ, Hiripi E, Mroczek DK, Normand S-LT, et al. Short screening scales to monitor population prevalences and trends in non-specific psychological distress. Psychol Med. 2002;32:959–76. Available from: http://www.journals.cambridge.org/abstract_S0033291702006074

44. WHO. Measuring Health and Disability: Manual for WHO Disability Assessment Schedule WHODAS 2.0. Üstun TB, Kostansjek N, Chatterji S, Rehm J, editors. Geneva, Switzerland: WHO; 2010.

45. Sulaiman-Hill CM, Thompson SC. Selecting instruments for assessing psychological wellbeing in Afghan and Kurdish refugee groups. BMC Res Notes. 2010;3:237.

46. Akhtar A, Cuijpers P, Morina N, Sijbrandij M, Bryant R. Exploratory and confirmatory factor analysis of the 12-item Arabic World Health Organization Disability Assessment Schedule (WHODAS 2.0) as a screening tool for Syrian refugees. BJPsych Open. 2021;7:1–7.

47. Slewa-Younan S, Mond JM, Bussion E, Melkonian M, Mohammad Y, Dover H, et al. Psychological trauma and help seeking behaviour amongst resettled Iraqi refugees in attending English tuition classes in Australia. Int J Ment Health Syst. 2015;9:1–6.

48. WHO. Problem Management Plus (PM+). Individual psychological help for adults impaired by distress in communities exposed to adversity. Generic fi. Geneva: WHO; 2016. Available from: http://www.who.int/entity/mental_health/emergencies/problem_management_plus/en/index.html

49. Bernal G, Sáez-Santiago E. Culturally centered psychosocial interventions. J Community Psychol. 2006;34:121–32.

50. Burchert S, Alkneme MS, Bird M, Carswell K, Cuijpers P, Hansen P, et al. User-centered app adaptation of a low-intensity e-mental health intervention for Syrian refugees. Front Psychiatry.

For peer review only


For peer review only - http://bmjopen.bmj.com/site/about/guidelines.xhtml

2019;10.

51. Akhtar A, Engels MH, Bawaneh A, Bird M, Bryant R, Cuijpers P, et al. Cultural Adaptation of a Low-Intensity Group Psychological Intervention for Syrian Refugees. Intervention. 2021;19:48–57.

52. Derogatis LR, Lipman RS, Rickels K, Uhlenhuth EH, Covi L. The Hopkins Symptom Checklist (HSCL): A self-report inventory. Behav Sci. 1974;19:1–15.

53. Mahfoud Z, Kobeissi L, Peters TJ, Araya R, Ghantous Z, Khoury B. The Arabic validation of the Hopkins Symptoms Checklist-25 against MINI in a disadvantaged suburb of Beirut, Lebanon. Int J Educ Psychol Assess. 2013;13:17–33.

54. Selmo P, Koch T, Brand J, Wagner B, Knaevelsrud C. Psychometric Properties of the Online Arabic Versions of BDI-II, HSCL-25, and PDS. Eur J Psychol Assess. 2016;35:46–54. Available from: http://econtent.hogrefe.com/doi/10.1027/1015-5759/a000367

55. Blevins CA, Weathers FW, Davis MT, Witte TK, Domino JL. The Posttraumatic Stress Disorder Checklist for DSM-5 (PCL-5): Development and initial psychometric evaluation. J Trauma Stress. 2015;28:489–98.

56. Ashworth M, Shepher M, Christey J, Matthews V, Wright K, Parmentier H, et al. A client-generated psychometric instrument: the development of “PSYCHLOPS.” Couns Psychother Res. 2004;4:27–32.

57. Ibrahim H, Ertl V, Catani C, Ismail AA, Neuner F. The validity of Posttraumatic Stress Disorder Checklist for DSM-5 (PCL-5) as screening instrument with Kurdish and Arab displaced populations living in the Kurdistan region of Iraq. BMC Psychiatry. BMC Psychiatry; 2018;18:259.

58. Acarturk C, Konuk E, Cetinkaya M, Senay I, Sijbrandij M, Gulen B, et al. The efficacy of eye movement desensitization and reprocessing for post-traumatic stress disorder and depression among Syrian refugees: Results of a randomized controlled trial. Psychol Med. 2016;46:2583–93.

For peer review only



For peer review only - http://bmjopen.bmj.com/site/about/guidelines.xhtml

59. Beecham J, Knapp M. Costing psychiatric interventions. In: Thornicroft G, Brewin C, Wing J, editors. Meas Ment Heal Needs. 1st ed. London: Gaskell; 1992. p. 163–83.

60. The GRADE Working Group. GRADE handbook for grading quality of evidence and strength of recommendations. Schünemann H, Brożek J, Guyatt G, Oxman A, editors. 2013. Available from: guidelinedevelopment.org/handbook

61. Balshem H, Helfand M, Schünemann HJ, Oxman AD, Kunz R, Brozek J, et al. GRADE guidelines: 3. Rating the quality of evidence. J Clin Epidemiol. 2011;64:401–6.

62. Evers S, Goossens M, De Vet H, Van Tulder M, Ament A. Criteria list for assessment of methodological quality of economic evaluations: Consensus on Health Economic Criteria. Int J Technol Assess Health Care. 2005;21:240–5.

63. R Core Team. R: A language and environment for statistical computing. Vienna, Austria: R Foundation for Statistical Computing; 2021.

64. Viechtbauer W. Conducting meta-analyses in R with the metafor. J Stat Softw. 2010;36:1–48.

65. Bates D, Mächler M, Bolker BM, Walker SC. Fitting linear mixed-effects models using lme4. J Stat Softw. 2015;67.

66. StataCorp. Stata Statistical Software. College Station, TX: StataCorp LLC; 2021.

67. Burke DL, Ensor J, Riley RD. Meta-analysis using individual participant data: one-stage and two-stage approaches, and why they may differ. Stat Med. 2017;36:855–75.

68. Bell ML, Fiero M, Horton NJ, Hsu CH. Handling missing data in RCTs; A review of the top medical journals. BMC Med Res Methodol. 2014;14:1–8.

69. White I, Horton N, Carpenter J, Pocock S. Strategy for intention to treat analysis in randomised trials with missing outcome data. BMJ. 2011;342:d40. Available from: https://www.ncbi.nlm.nih.gov/pmc/articles/PMC3230114/

70. Riley RD, Lambert PC, Abo-Zaid G. Meta-analysis of individual participant data: Rationale, conduct, and reporting. BMJ. 2010;340:521–5.

For peer review only


For peer review only - http://bmjopen.bmj.com/site/about/guidelines.xhtml

71. Higgins JPT, Thompson SG, Deeks JJ, Altman DG. Measuring inconsistency in meta-analyses. Br Med J. 2003;327:557–60.

72. Orsini N, Bottai M, Higgins J, Buchan I. Heterogi: Stata module to quantify heterogeneity in a meta-analysis. Boston: Boston College Department of Economics; 2005. p. S449201.

73. Egger M, Smith GD, Schneider M, Minder C. Bias in meta-analysis detected by a simple, graphical test. BMJ. 1997;315:629–34.

74. Duval S, Tweedie R. Trim and Fill: A Simple Funnel-Plot-Based Method. Biometrics. 2000;56:455–63.

75. Drescher A, Kiselev N, Akhtar A, Acarturk C, Bryant RA, Ilkkursun Z, et al. Problems after flight: understanding and comparing Syrians' perspectives in the Middle East and Europe. BMC Public Health. BMC Public Health; 2021;21:1–12.

76. Winhusen T, Winstanley EL, Somoza E, Brigham G. The potential impact of recruitment method on sample characteristics and treatment outcomes in a psychosocial trial for women with co-occurring substance use disorder and PTSD. Drug Alcohol Depend. Elsevier Ireland Ltd; 2012;120:225–8. Available from: http://dx.doi.org/10.1016/j.drugalcdep.2011.06.014

77. Fried EI, van Borkulo CD, Epskamp S, Schoevers RA, Tuerlinckx F, Borsboom D. Measuring depression over time...or not? lack of unidimensionality and longitudinal measurement invariance in four common rating scales of depression. Psychol Assess. 2016;28:1354–67.

78. Fokkema M, Smits N, Kelderman H, Cuijpers P. Response shifts in mental health interventions: an illustration of longitudinal measurement invariance. Psychol Assess. 2013;25:520–31.

For peer review only


For peer review only - http://bmjopen.bmj.com/site/about/guidelines.xhtml

Table 1.

*Trial Descriptions*

| Study site | Trial | Intervention | Setting | Inclusion criteria | Exclusion criteria | Pre-registration trial | Trial dates [a] |
|---|---|---|---|---|---|---|---|
| Netherlands | Pilot | PM+ | Community | STRENGTHS criteria [b] | STRENGTHS criteria [c] | NTR: NL6665 | April 2018-March 2019 |
| | RCT | PM+ | Community | STRENGTHS criteria [b] | STRENGTHS criteria [c]; *Current receipt of specialized psychological treatment* | NTR: NL7552 | Feb 2019-*ongoing* |
| Switzerland | Pilot | PM+ | Community | STRENGTHS criteria [b] | STRENGTHS criteria [c]; *Being under guardianship; Inability to follow study procedures* | ClinicalTrials.gov: NCT03830008 | Dec 2018-March 2020 |
| | RCT | PM+ | Community | STRENGTHS criteria [b] + *Arabic-speaking refugees 18+ yrs* | STRENGTHS criteria [c]; *Being under guardianship; Inability to follow study procedures* | ClinicalTrials.gov: NCT04574466 | Feb 2020-*ongoing* |
| Turkey | Pilot | gPM+ | Community | STRENGTHS criteria [b] | STRENGTHS criteria [c] | ClinicalTrials.gov: NCT03567083 | Sept 2018-Jan 2019 |
| | RCT | gPM+ | Community | STRENGTHS criteria [b] | STRENGTHS criteria [c] | ClinicalTrials.gov: NCT03960892 | Aug 2019-Nov 2021 |


For peer review only - http://bmjopen.bmj.com/site/about/guidelines.xhtml
For peer review only

| | | | | | | | |
|---|---|---|---|---|---|---|---|
| Jordan | Pilot | gPM+ | Refugee camp | STRENGTHS criteria [b] + *Having a child or dependent aged 10-16 yrs* | STRENGTHS criteria [c] | ANZCTR: ACTRN12619000168156 | Jan-April 2019 |
| | RCT | gPM+ | Refugee camp | STRENGTHS criteria [b] + *Having a child or dependent aged 10-16 yrs* | STRENGTHS criteria [c] | ANZCTR: ACTRN12619001386123 | Aug 2019-July 2020 |
| Germany | Pilot | SbS | Digital | STRENGTHS criteria [b] | *Signs of imminent suicide risk* | DRKS: DRKS00017838 | Dec 2019-Oct 2020 |
| | RCT | SbS | Digital | STRENGTHS criteria [b] | *Signs of imminent suicide risk* | DRKS: DRKS00022143 | Aug 2020-o*ngoing* |
| Sweden | RCT | SbS | Digital | STRENGTHS criteria [b] | *Signs of imminent suicide risk* | DRKS: DRKS00022144 | Aug 2020-*ongoing* |
| Egypt | RCT | SbS | Digital | STRENGTHS criteria [b] | *Signs of imminent suicide risk* | DRKS: DRKS00023505 | March 2021-o*ngoing* |

[a] Recruitment to 12-month follow-up; [b] STRENGTHS inclusion criteria = Arabic-speaking Syrian refugees 18+ yrs + K10 >15 + WHODAS 2.0 >16; [c] STRENGTHS exclusion criteria = Signs of imminent suicide risk, Acute medical conditions, Expressed acute needs or protection risks, and/or Indications of severe mental disorders; Pilot = pilot randomized controlled trial (RCT); RCT = fully-powered RCT; PM+ = Problem Management Plus; gPM+ = group PM+; SbS = Step-by-Step; K10 = Kessler Psychological Distress Scale; WHODAS 2.0 = WHO Disability Assessment Schedule 2.0; NTR = Netherlands Trial Register; ANZCTR = Australian New Zealand Clinical Trials Registry; DRKS: German Clinical Trials Register


For peer review only - http://bmjopen.bmj.com/site/about/guidelines.xhtml

**PRISMA-P (Preferred Reporting Items for Systematic review and Meta-Analysis Protocols) 2015 checklist: recommended items to address in a systematic review protocol***

| Section and topic | Item No | Checklist item | Page |
|---|---|---|---|
| **ADMINISTRATIVE INFORMATION** | | | |
| Title: | | | |
| Identification | 1a | Identify the report as a protocol of a systematic review | 1 |
| Update | 1b | If the protocol is for an update of a previous systematic review, identify as such | N/A |
| Registration | 2 | If registered, provide the name of the registry (such as PROSPERO) and registration number | OSF DOI 10.17605/OSF.IO/WUHGF |
| Authors: | | | |
| Contact | 3a | Provide name, institutional affiliation, e-mail address of all protocol authors; provide physical mailing address of corresponding author | 1-2 |
| Contributions | 3b | Describe contributions of protocol authors and identify the guarantor of the review | 14 |
| Amendments | 4 | If the protocol represents an amendment of a previously completed or published protocol, identify as such and list changes; otherwise, state plan for documenting important protocol amendments | N/A |
| Support: | | | |
| Sources | 5a | Indicate sources of financial or other support for the review | 14 |
| Sponsor | 5b | Provide name for the review funder and/or sponsor | 14 |
| Role of sponsor or funder | 5c | Describe roles of funder(s), sponsor(s), and/or institution(s), if any, in developing the protocol | 14 |
| **INTRODUCTION** | | | |
| Rationale | 6 | Describe the rationale for the review in the context of what is already known | 5-7 |
| Objectives | 7 | Provide an explicit statement of the question(s) the review will address with reference to participants, interventions, comparators, and outcomes (PICO) | 7 |
| **METHODS** | | | |
| Eligibility criteria | 8 | Specify the study characteristics (such as PICO, study design, setting, time frame) and report characteristics (such as years considered, language, publication status) to be used as criteria for eligibility for the review | 7-8 |
| Information | 9 | Describe all intended information sources (such as electronic databases, contact with study authors, trial registers or other | 7 |

For peer review only

| | | | |
|---|---|---|---|
| sources | | grey literature sources) with planned dates of coverage | |
| Search strategy | 10 | Present draft of search strategy to be used for at least one electronic database, including planned limits, such that it could be repeated | N/A |
| Study records: | | | |
| Data management | 11a | Describe the mechanism(s) that will be used to manage records and data throughout the review | 10 |
| Selection process | 11b | State the process that will be used for selecting studies (such as two independent reviewers) through each phase of the review (that is, screening, eligibility and inclusion in meta-analysis) | N/A |
| Data collection process | 11c | Describe planned method of extracting data from reports (such as piloting forms, done independently, in duplicate), any processes for obtaining and confirming data from investigators | 10 |
| Data items | 12 | List and define all variables for which data will be sought (such as PICO items, funding sources), any pre-planned data assumptions and simplifications | 10-12 |
| Outcomes and prioritization | 13 | List and define all outcomes for which data will be sought, including prioritization of main and additional outcomes, with rationale | 10 |
| Risk of bias in individual studies | 14 | Describe anticipated methods for assessing risk of bias of individual studies, including whether this will be done at the outcome or study level, or both; state how this information will be used in data synthesis | 11 |
| Data synthesis | 15a | Describe criteria under which study data will be quantitatively synthesised | 11-12 |
| | 15b | If data are appropriate for quantitative synthesis, describe planned summary measures, methods of handling data and methods of combining data from studies, including any planned exploration of consistency (such as $I^2$, Kendall's $\tau$) | 11-12 |
| | 15c | Describe any proposed additional analyses (such as sensitivity or subgroup analyses, meta-regression) | 11 |
| | 15d | If quantitative synthesis is not appropriate, describe the type of summary planned | N/A |
| Meta-bias(es) | 16 | Specify any planned assessment of meta-bias(es) (such as publication bias across studies, selective reporting within studies) | 12-13 |
| Confidence in cumulative evidence | 17 | Describe how the strength of the body of evidence will be assessed (such as GRADE) | 11 |

*** It is strongly recommended that this checklist be read in conjunction with the PRISMA-P Explanation and Elaboration (cite when available) for important clarification on the items. Amendments to a review protocol should be tracked and dated.** **The copyright for PRISMA-P (including checklist) is held by the PRISMA-P Group and is distributed under a Creative Commons Attribution Licence 4.0.**

*From: Shamseer L, Moher D, Clarke M, Ghersi D, Liberati A, Petticrew M, Shekelle P, Stewart L, PRISMA-P Group. Preferred reporting items for systematic review and meta-analysis protocols (PRISMA-P) 2015: elaboration and explanation. BMJ. 2015 Jan 2;349(jan02 1):g7647.*

For peer review only

For peer review only

BMJ Open

# Scalable Psychological Interventions for Syrian Refugees in Europe and the Middle East: STRENGTHS Study Protocol for a Prospective Individual Participant Data Meta-Analysis



| | |
|---|---|
| Journal: | *BMJ Open* |
| Manuscript ID | bmjopen-2021-058101.R2 |
| Article Type: | Protocol |
| Date Submitted by the Author: | 03-Mar-2022 |





| | |
|---|---|
| Complete List of Authors: | de Graaff, Anne; Vrije Universiteit Amsterdam, Department of Clinical, Neuro- and Developmental Psychology, World Health Organization Collaborating Center for Research and Dissemination of Psychological Interventions, Amsterdam Public Health Research Institute<br>Cuijpers, Pim; Vrije Universiteit Amsterdam, Department of Clinical, Neuro- and Developmental Psychology, World Health Organization Collaborating Center for Research and Dissemination of Psychological Interventions, Amsterdam Public Health Research Institute<br>Acarturk, Ceren ; Koç University, Department of Psychology<br>Akhtar, Aemal; University of New South Wales, School of Psychology<br>Alkneme, Mhd; Freie Universitat Berlin, Division of Clinical-Psychological Intervention, Department of Education and Psychology<br>Aoun, May; War Child, Research and Development Department<br>Awwad, Manar; International Medical Corps, Technical Unit<br>Bawaneh, Ahmad; International Medical Corps, Technical Unit<br>Brown, Felicity; War Child, Research and Development Department; University of Amsterdam, Amsterdam Institute of Social Science Research<br>Bryant, Richard ; University of New South Wales<br>Burchert, Sebastian; Freie Universitat Berlin, Division of Clinical-Psychological Intervention, Department of Education and Psychology<br>Carswell, Kenneth; World Health Organization, Department of Mental Health and Substance Abuse<br>Drogendijk, Annelieke; ARQ National Psychotrauma Centre, ARQ International<br>Engels, Michelle; International Federation of Red Cross and Red Crescent Societies Reference Centre for Psychosocial Support<br>Fuhr, Daniela; London School of Hygiene and Tropical Medicine, Department of Health Services Research and Policy<br>Hansen, Pernille; International Federation of Red Cross and Red Crescent Societies Reference Centre for Psychosocial Support<br>van 't Hof, Edith; World Health Organization, Department of Mental Health and Substance Abuse<br>Giardinelli, Luana; International Medical Corps, Technical Unit<br>Hemmo, Mahmoud; University Hospital Zurich, Department of Consultation-Liaison Psychiatry and Psychosomatic Medicine<br>Hessling, Jonas; Freie Universitat Berlin, Division of Clinical-Psychological Intervention, Department of Education and Psychology<br>Ilkkursun, Zeynep; Koç University, Department of Psychology |

| | |
|---|---|
| | Jordans, Mark J. D.; War Child, Research and Development Department; University of Amsterdam, Amsterdam Institute of Social Science Research<br>Kiselev, Nikolai; University Hospital Zurich, Department of Consultation-Liaison Psychiatry and Psychosomatic Medicine<br>Knaevelsrud, Christine; Freie Universitat Berlin, Division of Clinical-Psychological Intervention, Department of Education and Psychology<br>Kurt, Gulsah; Koç University, Department of Psychology<br>Martinmaki, Saara; ARQ National Psychotrauma Centre, ARQ International<br>McDaid, David ; The London School of Economics and Political Science, Care Policy and Evaluation Centre, Department of Health Policy<br>Morina, Naser; University Hospital Zurich, Department of Consultation-Liaison Psychiatry and Psychosomatic Medicine<br>Naser, Hadeel; International Medical Corps, Technical Unit<br>Park, A-La; London School of Hygiene and Tropical Medicine, Care Policy and Evaluation Centre, Department of Health Policy<br>Pfaltz, Monique; University Hospital Zurich, Department of Consultation-Liaison Psychiatry and Psychosomatic Medicine; Mid Sweden University, Department of Psychology and Social Work<br>Roberts, Bayard; London School of Hygiene and Tropical Medicine, Department of Health Services Research and Policy<br>Schick, Matthis; University Hospital Zurich, Department of Consultation-Liaison Psychiatry and Psychosomatic Medicine<br>Schnyder, Ulrich; University Hospital Zurich, Department of Consultation-Liaison Psychiatry and Psychosomatic Medicine<br>Spaaij, Julia; University Hospital Zurich, Department of Consultation-Liaison Psychiatry and Psychosomatic Medicine<br>Steen, Frederik; War Child, Research and Development Department<br>Taha, Karine; War Child, Research and Development Department<br>Uygun, Ersin; Istanbul Bilgi University, Trauma and Disaster Mental Health Master Programme<br>Ventevogel, Peter; United Nations High Commissioner for Refugees, Public Health<br>Whitney, Claire; International Medical Corps, Technical Unit<br>Witteveen, Anke; Vrije Universiteit Amsterdam, Department of Clinical, Neuro- and Developmental Psychology, World Health Organization Collaborating Center for Research and Dissemination of Psychological Interventions, Amsterdam Public Health Research Institute<br>Sijbrandij, Marit; Vrije Universiteit Amsterdam, Department of Clinical, Neuro- and Developmental Psychology, World Health Organization Collaborating Center for Research and Dissemination of Psychological Interventions, Amsterdam Public Health Research Institute |
| <b>Primary Subject Heading</b>: | Mental health |
| Secondary Subject Heading: | Global health, Health economics |
| Keywords: | MENTAL HEALTH, Anxiety disorders < PSYCHIATRY, Depression & mood disorders < PSYCHIATRY |
| | |

SCHOLARONE™ Manuscripts

# Scalable Psychological Interventions for Syrian Refugees in Europe and the Middle East: STRENGTHS Study Protocol for a Prospective Individual Participant Data Meta-Analysis

Anne M. de Graaff (a.m.de.graaff@vu.nl),[1] Pim Cuijpers (p.cuijpers@vu.nl),[1] Ceren Acarturk (cacarturk@ku.edu.tr),[2] Aemal Akhtar (a.akhtar@unsw.edu.au),[3] Mhd Salem Alkneme (salem.alkneme@fu-berlin.de),[4] May Aoun (mayaoun71@gmail.com),[5] Manar Awwad (mawwad@InternationalMedicalCorps.org),[6] Ahmad Y. Bawaneh (abawaneh@InternationalMedicalCorps.org),[6] Felicity L. Brown (felicity.brown@warchild.nl), [5, 7] Richard Bryant (r.bryant@unsw.edu.au),[3] Sebastian Burchert (s.burchert@fu-berlin.de),[4] Kenneth Carswell (carswellk@who.int),[8] Annelieke Drogendijk (a.drogendijk@arq.org),[9] Michelle Engels (mieng@rodekors.dk),[10] Daniela C. Fuhr (Daniela.Fuhr@lshtm.ac.uk),[11] Pernille Hansen (pehan@rodekors.dk),[10] Edith van 't Hof (vanthofe@who.int),[8] Luana Giardinelli (luana.giardinelli@gmail.com),[6] Mahmoud Hemmo (Mahmoud.Hemmo@usz.ch),[12] Jonas M. Hessling (j.hessling@fu-berlin.de),[4] Zeynep Ilkkursun (zilkkursun20@ku.edu.tr),[2] Mark J.D. Jordans (mark.jordans@warchild.nl),[5, 7] Nikolai Kiselev (Nikolai.Kiselev@usz.ch),[12] Christine Knaevelsrud (christine.knaevelsrud@fu-berlin.de),[4] Gulsah Kurt (gkurt18@ku.edu.tr),[2] Saara Martinmaki (s.martinmaki@impact.arq.org),[9] David McDaid (D.Mcdaid@lse.ac.uk),[13] Naser Morina (naser.morina@usz.ch),[12] Hadeel Naser (hnaser@InternationalMedicalCorps.org),[6] A-La Park (a.park@lse.ac.uk),[13] Monique C. Pfaltz (monique.pfaltz@miun.se) [12, 14] Bayard Roberts (bayard.roberts@lshtm.ac.uk),[11] Matthis Schick (matthis.schick@usz.ch),[12] Ulrich Schnyder (ulrich.schnyder@access.uzh.ch),[12] Julia Spaaij (julia.spaaij@usz.ch),[12] Frederik Steen (frederik.steen@warchild.nl),[5] Karine Taha (karine.taha@hotmail.com),[5] Ersin Uygun (ersin.uygun@bilgi.edu.tr),[15] Peter Ventevogel (ventevog@unhcr.org),[16] Claire Whitney (cwhitney@InternationalMedicalCorps.org),[6] Anke B. Witteveen (a.b.witteveen@vu.nl),[1] Marit Sijbrandij (e.m.sijbrandij@vu.nl),[1] on behalf of the STRENGTHS consortium


For peer review only - http://bmjopen.bmj.com/site/about/guidelines.xhtml

[1] Department of Clinical, Neuro- and Developmental Psychology, WHO Collaborating Center for Research and Dissemination of Psychological Interventions, Amsterdam Public Health Research Institute, Vrije Universiteit Amsterdam, Amsterdam, The Netherlands

[2] Koc University, Department of Psychology, Istanbul, Turkey

[3] School of Psychology, University of New South Wales, Sydney, Australia

[4] Division of Clinical-Psychological Intervention, Department of Education and Psychology, Freie Universität Berlin, Berlin, Germany

[5] Research and Development Department, War Child Holland, Amsterdam, The Netherlands

[6] Technical Unit, International Medical Corps, London, UK

[7] Amsterdam Institute of Social Science Research, University of Amsterdam, Amsterdam, the Netherlands

[8] Department of Mental Health and Substance Use, World Health Organization, Geneva, Switzerland

[9] ARQ National Psychotrauma Centre, ARQ International, Diemen, The Netherlands

[10] International Federation of Red Cross and Red Crescent Societies Reference Centre for Psychosocial Support, Copenhagen, Denmark

[11] London School of Hygiene and Tropical Medicine, Department of Health Services Research and Policy, London, UK

[12] University Hospital Zurich, Department of Consultation-Liaison Psychiatry and Psychosomatic Medicine, Zurich, Switzerland

[13] Care Policy and Evaluation Centre, Department of Health Policy, London School of Economics and Political Science, London, UK

[14] Department of Psychology and Social Work, Mid Sweden University, Ostersund, Sweden

[15] Bilgi University, Trauma and Disaster Mental Health Master Programme, Istanbul, Turkey

[16] United Nations High Commissioner for Refugees, Public Health Section, Genève, Switzerland

For peer review only


For peer review only - http://bmjopen.bmj.com/site/about/guidelines.xhtml

**Corresponding author:** Anne M. de Graaff, a.m.de.graaff@vu.nl, Department of Clinical, Neuro- and Developmental Psychology, WHO Collaborating Center for Research and Dissemination of Psychological Interventions, Amsterdam Public Health Research Institute, Vrije Universiteit Amsterdam, Van der Boechorststraat 7, 1081 BT, Amsterdam, The Netherlands

For peer review only

**Word count**: 4069

**Abstract word count**: 290


For peer review only - http://bmjopen.bmj.com/site/about/guidelines.xhtml

**Abstract**

**Introduction**

The World Health Organization's (WHO) scalable psychological interventions, such as Problem Management Plus (PM+) and Step-by-Step (SbS) are designed to be cost-effective non-specialist delivered interventions to reduce symptoms of common mental disorders, such as anxiety, depression and posttraumatic stress disorder (PTSD). The STRENGTHS consortium aims to evaluate the effectiveness, cost-effectiveness and implementation of the individual format of PM+ and its group version (gPM+), as well as of the digital SbS intervention among Syrian refugees in seven countries in Europe and the Middle East. This is a study protocol for a prospective individual participant data (IPD) meta-analysis to evaluate 1) overall effectiveness and cost-effectiveness, and 2) treatment moderators of PM+, gPM+ and SbS with Syrian refugees.

**Methods and analysis**

Five pilot RCTs and seven fully-powered RCTs conducted within STRENGTHS will be combined into one IPD meta-analytic dataset. The RCTs include Syrian refugees of 18 years and above with elevated psychological distress (Kessler Psychological Distress Scale (K10 >15)) and impaired daily functioning (WHO Disability Assessment Schedule 2.0 (WHODAS 2.0 >16)). Participants are randomized into the intervention or care as usual control group, and complete follow-up assessments at 1-week, 3-month and 12-month follow-up. Primary outcomes are symptoms of depression and anxiety (25-item Hopkins Symptom Checklist). Secondary outcomes include daily functioning (WHODAS 2.0), PTSD symptoms (PTSD Checklist for DSM-5), and self-identified problems (PSYCHLOPS). We will conduct a one-stage IPD meta-analysis using linear mixed models. Quality of evidence will be assessed using the GRADE approach, and the economic evaluation approach will be assessed using the CHEC-list.

**Ethics and dissemination**

For peer review only


For peer review only - http://bmjopen.bmj.com/site/about/guidelines.xhtml

Local ethical approval has been obtained for each RCT. This IPD meta-analysis does not require ethical approval. The results of this study will be published in international peer-reviewed journals.

**Registration**

Pre-registered on OSF: DOI 10.17605/OSF.IO/WUHGF

**Strengths and limitations of this study**

- An advantage of individual participant data (IPD) meta-analysis concerns the acquired power to study overall treatment effect estimates, predictors and moderators.
- Selected randomized controlled trials are intentionally similar in study design, outcome measures and target population to allow for optimal pooling of data.
- The study sample will be large (estimated > 1000 participants) and relatively homogeneous (i.e., Syrian refugees).
- The meta-analyses will have reduced power in case individual RCTs do not meet the required sample size.

**Key words:** Mental Health; Anxiety Disorders; Depression and Mood Disorders

For peer review only


For peer review only - http://bmjopen.bmj.com/site/about/guidelines.xhtml

## Introduction

Since the outbreak of the civil war in Syria, over 13 million Syrians have been displaced. The leading destinations of Syrians displaced across borders include Turkey (3.6 million), Lebanon (910,600), and Jordan (654,700). One million fled to Europe by boat or overland [1]. Refugees often reside in unstable and insecure locations in urban areas or refugee camps [2–4], and may face uncertainty about their legal status in seeking asylum [5]. In this paper, we use 'Syrian refugees' to refer to Syrians who have been granted a refugee status and to those whose request for sanctuary still has to be processed. Due to their exposure to potentially traumatic events and other stressors before, during, and after displacement, refugees are at risk of developing mental disorders [6–9]. However, health systems are often under-resourced to respond to the mental health needs of refugee populations, especially in low- and middle-income countries, where health systems are already overburdened [10,11]. The rapidly escalated refugee crisis also poses challenges for health systems in well-resourced settings in Europe [12], where mental health services are underutilized due to barriers such as communication difficulties and fear of stigma, and access is hampered by lack of culturally appropriate mental health services and long wait lists [13–15].

The Syrian REfuGees MeNTal HealTH Care Systems (STRENGTHS) research consortium aims to evaluate the effectiveness, cost-effectiveness, and implementation of scalable psychological interventions among Syrian refugees in Europe and the Middle-East [16]. The interventions have been developed by the World Health Organization to scale up coverage for priority conditions, including depression and anxiety [17], to improve access to mental health and psychosocial support in resource-constrained settings [18]. Scalable interventions are strongly protocolized and use evidence-based psychological techniques from therapies such as cognitive behavioural therapy and interpersonal therapy. Typical features include the duration of the intervention (i.e., few sessions instead of lengthy treatment programs), the target of the intervention (i.e., transdiagnostic instead of focused on a single disorder), and the delivery agent (i.e., task-shifting to non-specialist providers) [17,19].


For peer review only - http://bmjopen.bmj.com/site/about/guidelines.xhtml

One of the interventions that is currently being tested by the STRENGTHS consortium is Problem Management Plus (PM+), which can be delivered by trained and supervised non-specialists to reduce psychological distress in communities affected by adversity [20]. The first randomized controlled trials (RCTs) on PM+ have been conducted in communities affected by violence in Pakistan and Kenya [21–23], and in a disaster-prone community in Nepal [24]. PM+ for individuals led to improvements in anxiety, depression, posttraumatic stress disorder (PTSD), psychosocial functioning, and self-identified problems three months after the intervention [21,23]. Although PM+ was more effective in reducing symptoms of common mental disorders, it was also costlier compared to CAU alone [25]. PM+ delivered in groups (gPM+) to women in Pakistan was effective in reducing symptoms of anxiety, depression, and self-identified problems, but not for symptoms of PTSD [22]. In Nepal, gPM+ for men and women was also associated with improvements in psychological distress, depression, and “heart-mind” problems, but not for psychosocial functioning and symptoms of PTSD [24]. Another non-specialist delivered intervention based on PM+ that is currently being tested by the STRENGTHS consortium is the digital Step-by-Step (SbS) intervention [26]. SbS is a minimally guided e-mental health intervention with ‘e-helper’ support. Pilot studies on SbS in Lebanon, including Syrian participants, found that the intervention was acceptable [27], and likely effective in improving psychosocial outcomes [28].

Pilot studies among Syrian refugees carried out by the STRENGTHS consortium indicated that PM+ delivered by peers is an acceptable and feasible intervention, and likely effective in reducing psychological distress [29–32]. Currently, fully-powered (i.e., based on a priori power analysis) pragmatic RCTs among adult Syrian refugees in Europe and the Middle East are being conducted to test the effectiveness of PM+ in individual format in Switzerland and the Netherlands [33], PM+ in group format (gPM+) in Jordan [34] and Turkey [35], and the digital Step-by-Step (SbS) intervention in Germany, Sweden and Egypt [16]. The interventions are compared with care as usual (CAU). CAU refers to all (mental) health services available to refugees in the setting where the pragmatic trial is


For peer review only - http://bmjopen.bmj.com/site/about/guidelines.xhtml

conducted [36]. Although study sites differ with regard to the availability of services, barriers to accessing care have been identified across host countries [13,15,37].

Initial results of PM+/gPM+ delivered by trained peer-refugees are promising when looking at overall effects. However, individual differences in symptom severity, socio-demographic background, comorbidities, living circumstances, and life events may result in individual differences in treatment response. Single trials do not have sufficient power to detect genuine interaction effects between participant characteristics and treatment outcome [38]. To determine which individuals are more or less likely to benefit from these scalable psychological interventions, an individual participant data (IPD) meta-analytic approach can be used [39,40]. This approach does not rely on aggregate (study-level) data used in conventional meta-analysis techniques, but uses the original data of each individual participant (participant-level) from eligible RCTs. The IPD meta-analysis approach offers greater statistical power and precision to better understand the predictive nature of individual characteristics on treatment outcome [38,41]. Furthermore, it also allows us to get a better estimate of overall effectiveness and cost-effectiveness, by pooling several large data sets together.

This protocol outlines a planned IPD meta-analysis on combined datasets from the STRENGTHS project, aiming to examine 1) the overall effectiveness and cost-effectiveness of scalable psychological interventions (i.e., PM+, gPM+, SbS) as compared to CAU alone on improving depression and anxiety (primary outcomes), PTSD symptoms, psychosocial functioning, and self-identified problems (secondary outcomes) among Syrian refugees, and 2) sociodemographic, migratory, and clinical predictors and moderators of treatment outcome (i.e., treatment-covariate interactions).

## Methods

This protocol was pre-registered in Open Science Framework (DOI 10.17605/OSF.IO/WUHGF), and all individual RCTs were prospectively registered online (see Table 1). The PRISMA-P checklist [42]



For peer review only - http://bmjopen.bmj.com/site/about/guidelines.xhtml

is appended. The IPD meta-analysis, including merging all datasets and data analyses will be conducted between January 2022 and December 2022.

[INSERT TABLE 1 ABOUT HERE]

**Patient and public involvement**

The STRENGTHS project installed an independent external expert board (Project Advisory Board) including mental health professionals from Syria. The expert board is regularly consulted on the quality of the results and output of STRENGTHS, as well as on the dissemination and exploitation of the results. Prior to conducting the RCTs, qualitative studies among Syrian refugees in each participating country were conducted to inform intervention development, study design and recruitment strategies.

**Inclusion of datasets**

We will include all STRENGTHS randomized controlled trials (RCTs) on the effectiveness of PM+ for individuals (Switzerland and the Netherlands), PM+ for groups (gPM+) (Jordan and Turkey), and SbS (Sweden, Germany and Egypt). These RCTs involve a relatively homogeneous population, and have similar study design and outcomes measures. The trials are presented in Table 1.

**Participants and procedure**

In the trials, Arabic-speaking Syrian refugees of 18 years and above are included if they report psychological distress (Kessler Psychological Distress Scale (K10 >15)) [43] and impaired daily functioning (WHO Disability Assessment Schedule 2.0 (WHODAS 2.0 >16)) [44]. The K10 and WHODAS have been found to be valid screening instruments among refugee populations [45–47].



For peer review only - http://bmjopen.bmj.com/site/about/guidelines.xhtml

For peer review only

The fully-powered RCT on PM+ in Switzerland also includes Arabic-speaking refugees from other countries. In Jordan, an additional criterion is having a child/dependent between the ages 10-16 years.

Across trials, participants are excluded if there are signs of imminent suicide risk (PM+ manual questionnaire) [48]. Furthermore, all trials except those on SbS in Sweden, Germany and Egypt exclude participants with acute medical conditions, expressed acute needs or protection risks, and indications of severe mental disorders (e.g., psychotic disorders or substance-dependence) or cognitive impairment (e.g., severe intellectual disability or dementia) (PM+ manual observation checklist) [48]. The fully-powered PM+ trial in the Netherlands also excludes participants currently receiving specialized psychological treatment. Other exclusion criteria in Switzerland include being under guardianship and inability to follow study procedures.

For peer review only

In line with the main aim of the STRENGTHS project, the IPD meta-analysis will include only Syrian refugees. The RCTs in Turkey, Switzerland and the Netherlands are conducted in a community setting, while the RCTs in Jordan are conducted in a refugee camp. At this moment, all pilot trials have been completed, with sample sizes ranging from 46 to 64 (total *N*=302). Recruitment has been completed for the fully-powered trials in Turkey (*N*=369), Jordan (*N*=410), and Egypt (*N*±538). All trials randomise individuals 1:1 to the intervention (PM+/CAU, gPM+/CAU, or SbS/CAU) or control group (CAU only). Assessments are conducted at baseline, one-week after the intervention, three months after the intervention, and 12 months after baseline. The 12-month assessment was not part of the pilot RCTs. The pilot RCT in Jordan only included a baseline and one-week follow-up assessment. Treatment conditions are further explained below.

**The interventions**

The manuals for all interventions were adapted by the STRENGTHS consortium according to a framework for the cultural adaptation of psychological interventions [49], and materials to provide training for trainers and helpers/facilitators were developed [50,51].


For peer review only - http://bmjopen.bmj.com/site/about/guidelines.xhtml

**Problem Management Plus (PM+) for individuals and groups**

PM+ is a brief, transdiagnostic psychological intervention developed to reduce symptoms of common mental disorders such as depression, anxiety, and PTSD [20]. PM+ includes five weekly sessions provided by trained non-specialist helpers. During this 5-week intervention, the participant learns skills in arousal reduction, problem-solving, behavioural activation and accessing social support. These strategies include a relaxation exercise using slow breathing (session 1), a 7-step plan to manage practical problems (session 2), behavioural activation by re-engaging with pleasant and task-oriented activities (session 3), and a strategy to strengthen social support (session 4). Homework is scheduled between sessions and strategies are reviewed in each subsequent session. Session 5 focuses on relapse prevention. The individual PM+ intervention consists of five 90-minute sessions. The group PM+ intervention consists of five 2-hour sessions in groups of 6-10 participants of the same gender led by two gender-matched group facilitators [20,51].

PM+ is delivered by Arabic-speaking peer-refugees who have completed high school, and have a background in education, social work, health care, or another related field. In Jordan, group facilitators were members of the host community. Helpers/group facilitators in Switzerland, the Netherlands and Turkey also needed to be proficient in English or the local language (i.e., German, Dutch or Turkish). Helpers/group facilitators received an eight-day training of helpers (ToH) on common mental disorders, basic counselling skills, delivery of intervention strategies and self-care. The ToH was followed by practice cases. Helpers/group facilitators receive weekly supervision by trained PM+ trainers and supervisors throughout the trial. PM+ trainers/supervisors are mental health care professionals who received a five-day training of trainers (ToT) on elements covered in the ToH, as well as on training and supervision skills. They receive monthly supervision by a PM+ master trainer [20].

For peer review only


For peer review only - http://bmjopen.bmj.com/site/about/guidelines.xhtml

### Step-by-Step (SbS)

The SbS intervention is a 5-session, digital self-help intervention for iOS, Android and Web platforms, aimed at reducing depressive symptoms. The strategies that are introduced in this 5-week intervention include psychoeducation and trying small and pleasant activities (session 1), behavioural activation (session 2), stress management (session 3), accessing social support (session 4), and positive self-verbalization and relapse prevention (session 5). Homework practice is scheduled between sessions. WHO removed problem-solving from SbS, as it was found to be too complex to implement in a digital application with or without helper support [26]. The intervention uses an illustrated, narrative story of a fictional character that seeks help for depression from a professional. The narrative provides educative information on the strategies, supplemented by interactive exercises (e.g., activity scheduling). Sessions last 30 minutes, and involve an introduction part, practice part, and reinforcement part with optional contact-on-demand to non-specialist, Arabic-speaking "e-helpers" available through a text and audio messaging system. E-helpers are university students trained and supervised by mental health professionals on basic helping skills, providing basic support, and on the identification, assessment and management of risk and (serious) adverse events.

### Care as usual (CAU)

The identified studies compared PM+, gPM+ or SbS in addition to CAU, to CAU alone. CAU includes all (mental) health services available to refugees across settings. In high-income countries, this includes referral to (specialized) mental health services through primary care practitioners (e.g., general practitioner). In Turkey, refugees have free access to mental health services in public hospitals and migrant health centres. Refugees residing in refugee camps in Jordan can access services inside the camp provided by national and international non-governmental organisations. However, across sites, access to mental health services is hampered by numerous barriers, such as the lack of Arabic-speaking health providers in European countries, and overburdened health systems of neighbouring


For peer review only - http://bmjopen.bmj.com/site/about/guidelines.xhtml
For peer review only

countries hosting most refugees [13,14]. All study participants received information about available mental health services.

**Outcomes**

Similar to the individual RCTs, the primary outcomes in this IPD meta-analysis will be symptoms of depression and anxiety at 3-month follow-up, measured by the subscales of the Hopkins Symptom Checklist (HSCL-25) [52–54]. Secondary outcomes include daily functioning (WHODAS 2.0), PTSD symptoms (PTSD Checklist for DSM-5; PCL-5) [55], and self-identified problems (PSYCHLOPS) [56]. Measures were selected based on their acceptable validity and reliability in refugee and Arabic-speaking groups, as well as their availability in the Arabic language and prior use with Syrian refugees [45,46,53,54,57,58]. We will also examine these outcomes at the 1-week post-assessment and 12-month follow-up.

To calculate cost-effectiveness, in addition to documented resources required for intervention delivery, we will examine the pooled changes in resource use associated with use of health-related services, and time out of usual activities that have been collected using an adapted version of the Client Service Receipt Inventory (CSRI) [59]. All measures were pilot tested using cognitive interviews with members of the target community [34].

**Study-level variables**

We will obtain study-level data for each trial including country and setting in which the study was conducted, and the type of intervention tested (e.g., PM+, gPM+, SbS). We will thus test overall effectiveness for all interventions together as well as the differential effect of each type of intervention.

**Individual-level variables**


For peer review only - http://bmjopen.bmj.com/site/about/guidelines.xhtml

We will extract individual-level data from each dataset to test a range of variables that may moderate the effects of the interventions on primary and secondary outcomes, to determine differential treatment effects based on participant characteristics. We will gather and synthesize all socio-demographic variables (i.e., age, gender, education level, work status, marital status), clinical characteristics (i.e., symptoms of depression, anxiety and PTSD, daily functioning, the exposure and type of traumatic events, post-migration stressors), and migration variables (i.e., time spent in host country, refugee status).

**IPD collection and aggregation**

All research teams have agreed to contribute RCT data to these IPD meta-analyses. De-identified primary datasets will be shared using secure password protected data links. Data will be stored in a secure cloud service (Surfdrive) developed for the Dutch education and research community, which can only be accessed by the IPD meta-analysis research team. Data transfer, storage and handling will follow the EU General Data Protection Regulations (GDPR).

Data accuracy of the primary datasets will be examined to explore if it matches data reported in published reports. We will specifically check frequencies of socio-demographic variables (e.g., age, gender, marital status, education level) and descriptive statistics (e.g., mean scores) of continuous scales. If discrepancies between the primary dataset and published report arise, clarification will be sought from authors. After the primary datasets are checked for accuracy, all eligible datasets will be merged into the IPD meta-analytic dataset.

**Quality of evidence**

We will assess the quality of evidence by applying the Grading of Recommendations, Assessment, Development and Evaluation (GRADE) approach [60]. The GRADE approach is a system for rating the quality (i.e., high, moderate, low, and very low) applied to a body of evidence, and not to the


For peer review only - http://bmjopen.bmj.com/site/about/guidelines.xhtml

individual studies. GRADE offers a transparent and structured process for developing and presenting evidence summaries and for carrying out the steps involved in developing recommendations. The GRADE approach consists of rating eight criteria, including five that may lead to rating down the quality of evidence (i.e., risk of bias, inconsistency, indirectness, imprecision, publication bias) and three that may lead to rating up (i.e., large effect, dose-response gradient, minimal influence of residual plausible confounding). Results will be summarized in a GRADE evidence profile [61]. For the economic analysis, we will assess the economic evaluation approach using the Consensus Health Economic Criteria list (CHEC-list) [62].

**The IPD meta-analysis**

All analyses will be conducted in R version 4.0.3 [63] using the packages metafor [64] and lme4 [65]. The economic evaluation will be conducted in STATA statistical software [66]. We will use a 'one-stage IPD meta-analytic' approach, in which the IPD of all identified studies will be merged into one large dataset. The resulting dataset is analysed in one step, as if all participants, clustered within studies, belonged to a single trial. This approach is preferred over the 'two-stage IPD meta-analytic' approach, in which aggregated summary points are pooled in an appropriate meta-analysis model. The one-stage approach allows for more advanced modelling of the moderators, can adopt more appropriate likelihood functions and has fewer assumptions [67].

The IPD meta-analysis will be conducted according to the intention-to-treat principle (i.e., all randomized participants will be included in the analyses). Missing values for outcome data will be estimated under the missing-at-random assumption, using multiple imputation (100 imputations) [68,69]. To estimate the missing values, valid predictor variables such as individual clinical and socio-demographic variables will be used (e.g., distress levels at baseline, age, gender). Sensitivity analyses will be conducted on complete cases only (i.e., with outcome data) to test for differences between imputed and completed values, and with completers of the interventions only.


For peer review only - http://bmjopen.bmj.com/site/about/guidelines.xhtml

To examine the effects of PM+, gPM+ and SbS on primary and secondary outcomes at 3-month follow-up, we will perform a mixed effect linear regression model with random intercepts with each trial having a random effect and a fixed effect for the intervention and the outcome measures. The severity of depressive and anxiety symptoms, PTSD symptoms, daily functioning, and self-identified problems, and costs of care at the 3-month follow-up will be used as dependent variables, and condition (e.g., treatment versus control) will be the independent variable while adjusting for baseline scores. The standardized $\beta$ coefficient indicates how many standard deviations (*SD*) the dependent variables (i.e., anxiety, depression, PTSD, daily functioning, self-identified problems, and costs of care) change per *SD* increase in the independent variable. Thus, the larger the $\beta$ coefficient, the greater the effect of the independent variable on the respective dependent variable, with no association among the variables if $\beta$ is 0.

To ensure robustness of our findings, the analysis of the primary outcomes will be repeated using the ‘two-stage IPD meta-analytic approach’. In a two-stage approach, Cohen’s *d* will be calculated for all outcomes for every single trial separately (step 1), and will then be combined to calculate the pooled effect sizes using the random-effects model (step 2) [70].

For the economic analysis incremental cost-effectiveness ratios comparing changes in effect and costs between the intervention group and controls on the primary outcome will also be estimated. Uncertainty in mean differences in intervention resource use as well as the use of health and other services between baseline and 3-month follow-up between the two groups will be accounted for using bias-corrected and accelerated bootstrapping. Purchasing power parity adjusted unit costs expressed in international dollars will be applied to resource use. Discounting will not be applied given the short duration of the study. Cost-effectiveness acceptability curves (CEACs) will be generated to showing the likelihood that intervention is cost-effective at different willingness-to-pay thresholds.

**Participant- and study-level moderators**





For peer review only - http://bmjopen.bmj.com/site/about/guidelines.xhtml

For peer review only

We will test whether socio-demographic, clinical and migration variables moderate the effects of psychological interventions among Syrian refugees at 3-month follow-up. To examine the effects of potential moderators, we will add an interaction term between each moderator variable and condition on depressive and anxiety symptoms into the mixed-effects linear/logistic regression models. Each potential moderator variable will be added into separate bivariate models.

**Heterogeneity**

We will calculate the $I^2$ statistic to assess statistical heterogeneity indicated in percentages, with 0% indicating no heterogeneity, 25% low heterogeneity, 50% moderate heterogeneity, and 75% high heterogeneity [71]. We will also calculate 95% Confidence Intervals (CI) around $I^2$ using the non-central chi-squared-based approach to provide the full magnitude of heterogeneity [72].

**Small sample bias**

The present study protocol describes a prospective IPD meta-analysis of RCTs conducted within the STRENGTHS project. We will examine small sample bias by inspecting the funnel plot in which sample size is plotted against the effect estimates of the primary outcome measures. Egger's test of the intercept will be carried out to inspect the degree of asymmetry of the plot [73]. Lastly, we will estimate the effect size adjusted for bias using the trim and fill procedure [74].

**Ethics and dissemination**

Local ethical approval has been obtained for each RCT. This IPD meta-analysis does not require ethical approval. The results of this study will be published in international peer-reviewed journals, and will be made publicly available through the STRENGTHS website (www.STRENGTHS-project.eu).



For peer review only - http://bmjopen.bmj.com/site/about/guidelines.xhtml

## Discussion

Scalable psychological interventions such as PM+, gPM+ and SbS for communities affected by adversity have been found to be effective in various RCTs [21–24]. Pilot studies on PM+ adapted for Syrian refugees indicated the intervention's feasibility and likely effectiveness when delivered by peer-refugees to individuals [30,31] or groups [29,32]. The STRENGTHS consortium tests the effectiveness of different types of scalable interventions (i.e., PM+, gPM+ and SbS) among Syrian refugees across different settings (e.g., refugee camp, community setting). The present paper describes the procedures of an IPD meta-analysis aimed at examining the overall effectiveness and cost-effectiveness of the interventions compared with CAU alone, and at examining individual participant differences in treatment response.

This study protocol has several strengths. An essential advantage of IPD meta-analytic approaches over using conventional meta-analytic approaches concerns the acquired power to study overall effect estimates, predictors and moderators, by synthesizing the original data from all individual RCTs into one large dataset. Single RCTs frequently do not have enough statistical power to investigate moderators. In conventional meta-analysis, moderation analyses are commonly done using subgroup analyses of aggregate data (e.g., effect sizes), but this limits statistical power and accuracy due to a loss of degrees of freedom and variability in the moderator of interest [67]. IPD meta-analysis also allows us to investigate outcome variables that primary studies have not reported. Another strength of our protocol concerns the availability of study trials with similar population, study design and outcome variables. This enables us to determine who benefits more or less from scalable psychological interventions using a meta-analytic approach that is least biased and most reliable for addressing this question.

This study has important limitations that have to be taken into account when interpreting the results. First, overall statistical power may be reduced in case individual RCTs do not meet the required sample size based on a priori power calculation. Second, study participants reside in different


For peer review only - http://bmjopen.bmj.com/site/about/guidelines.xhtml

settings, including refugee camps with limited access to mental health care, and community settings in high-income countries where specialist services are widely available although not always easily accessible to refugees. Although we will register the CAU delivered at each site using the CSRI measure, it cannot be precluded that differences in CAU may affect the results. Furthermore, migratory patterns as well as the ongoing stressors might differ considerably across settings [1,75]. Third, the use of different recruitment strategies across and within trials may have affected sample composition and treatment effects [76]. Fear of stigma has been found to be one of the reasons refugees may not access mental health services [15]. For trials where participants are mainly self-selected (e.g., through social media campaigns in Germany or the Netherlands), participants may also hold more positive attitudes towards mental health services. Participants in Jordan, however, were actively recruited door-to-door by members of the research team. Thus, study samples across sites may systematically differ from each other regarding their motivation to engage in mental health services, which in turn may affect the interpretation of the IPD meta-analysis results [76]. Furthermore, the results may be influenced by response-shifts at follow-up on self-report measures such as the HSCL-25 (e.g., lack of unidimensionality and temporal invariance) [77], especially when response-shifts differ between treatment and control groups [e.g., 78].

This IPD meta-analysis protocol will extend our current knowledge on the effectiveness of scalable psychological interventions for refugees and other populations affect by adversity. It will allow for more accurate estimations of treatment effects, cost-effectiveness, and the exploration of important predictors and treatment moderators of scalable interventions for sub-groups of refugees in Europe and the Middle East. The results of this study will be disseminated through publication in peer-reviewed journals.

**Authors' contributions**


For peer review only - http://bmjopen.bmj.com/site/about/guidelines.xhtml

AG drafted the study protocol. AG, PC, CA, AA, MSA, MAw, AB, RB, SB, ME, DF, LG, MH, JH, ZI, NK, CK, GK, DM, NM, HN, AP, MP, RB, MSch, US, JS, EU, CW, AW and MSij contributed to the original data acquisition. DM and AP provided input for the economic analysis. AG, PC, CA, AA, MSA, MAo, MAw, AB, FB, RB, SB, KC, AD, ME, DF, PH, EH, LG, MH, JH, ZI, MJ, NK, CK, GK, SM, DM, NM, HN, AP, MP, BR, MSch, US, FS, JS, KT, EU, PV, CW, AW and MSij all provided input for the study design. AG, PC, CA, AA, MSA, MAo, MAw, AB, FB, RB, SB, KC, AD, ME, DF, PH, EH, LG, MH, JH, ZI, MJ, NK, CK, GK, SM, DM, NM, HN, AP, MP, BR, MSch, US, FS, JS, KT, EU, PV, CW, AW and MSij all read, commented and approved the final protocol. MSij is the guarantor of the protocol. This protocol is written on behalf of the STRENGTHS consortium.

**Funding**

The STRENGTHS project is funded under Horizon 2020 – the Framework Programme for Research and Innovation (2014-2020). The authors alone are responsible for the views expressed in this article and they do not necessarily represent the views, decisions or policies of the institutions with which they are affiliated. The European Community is not liable for any use that may be made of the information contained therein.

**Competing interests**

David McDaid: Payment to institution for advice (not related to the interventions evaluated in STRENGTHS) given to the British Association for Counselling and Psychotherapy in January 2022.

**Patient consent for publication**

Not required.

**Data**

The Vrije Universiteit Amsterdam (VU) will keep a central data repository of all data collected in the STRENGTHS project. The data will be available upon reasonable request to the STRENGTHS


For peer review only - http://bmjopen.bmj.com/site/about/guidelines.xhtml

consortium. Data access might not be granted to third parties when this would interfere with relevant data protection and legislation in the countries participating in this project and any applicable EU legislation regarding data protection. Interested researchers can contact Dr Marit Sijbrandij at e.m.sijbrandij@vu.nl to initiate the process.

**ORCID IDs**

Anne M. de Graaff https://orcid.org/0000-0001-6686-4432

Pim Cuijpers https://orcid.org/0000-0001-5497-2743

Ceren Acarturk https://orcid.org/0000-0001-7093-1554

Aemal Akhtar https://orcid.org/0000-0002-8510-3636

Mhd Salem Alkneme https://orcid.org/0000-0002-4593-6705

Felicity Brown: https://orcid.org/0000-0001-6800-1657

Richard Bryant https://orcid.org/0000-0002-9607-819X

Sebastian Burchert https://orcid.org/0000-0003-3126-5485

Kenneth Carswell: https://orcid.org/0000-0001-5344-5802

Michelle Engels https://orcid.org/0000-0002-3782-1609

Daniela C. Fuhr https://orcid.org/0000-0001-9020-4629

Pernille Hansen https://orcid.org/0000-0002-4782-458X

Mahmoud Hemmo https://orcid.org/0000-0002-2958-0702

Edith van 't Hof https://orcid.org/0000-0002-5856-3573

Zeynep Ilkkursun https://orcid.org/0000-0001-6691-1809

Mark J.D. Jordans https://orcid.org/0000-0001-5925-8039

Nikolai Kiselev https://orcid.org/0000-0003-0617-0353

Christine Knaevelsrud https://orcid.org/0000-0003-1342-7006

Saara E. Martinmäki https://orcid.org/0000-0003-0230-4641



For peer review only - http://bmjopen.bmj.com/site/about/guidelines.xhtml

David McDaid https://orcid.org/0000-0003-0744-2664

Naser Morina https://orcid.org/0000-0002-6470-4408

A-La Park https://orcid.org/0000-0002-4704-4874

Monique Pfaltz https://orcid.org/0000-0002-7762-6226

Ulrich Schnyder https://orcid.org/0000-0003-3556-7990

Marit Sijbrandij https://orcid.org/0000-0001-5430-9810

Peter Ventevogel https://orcid.org/0000-0002-3567-8861

Claire Whitney https://orcid.org/0000-0002-1100-5363

Anke Witteveen https://orcid.org/0000-0002-9636-7522

For peer review only



For peer review only - http://bmjopen.bmj.com/site/about/guidelines.xhtml

## Literature

1. UNHCR. Trends at a glance: Global trends forced displacement in 2019. 2020. Available from: https://www.unhcr.org/5ee200e37.pdf

2. Silove D, Ventevogel P, Rees S. The contemporary refugee crisis: an overview of mental health challenges. World Psychiatry. 2017;16:130–9.

3. Alshawawreh LA, Smith RS, Wood JB. Assessing the Sheltering Response in the Middle East: Studying Syrian Camps in Jordan. Int Sch Sci Res Innov. 2017;11:1751–7. Available from: https://reliefweb.int/sites/reliefweb.int/files/resources/01 Azraq Fact Sheet January 2021.pdf

4. UNHCR. Zaatari Camp Fact Sheet. 2021. Available from: https://reliefweb.int/sites/reliefweb.int/files/resources/01 Zaatari Fact Sheet January 2021.pdf

5. Cange CW, Brunell C, Acarturk C, Fouad FM. Considering chronic uncertainty among Syrian refugees resettling in Europe. Lancet Public Heal. 2019;4:e14. Available from: http://dx.doi.org/10.1016/S2468-2667(18)30261-5

6. Steel Z, Chey T, Silove D, Marnane C, Bryant RA, Van Ommeren M. Association of torture and other potentially traumatic events with mental health outcomes among populations exposed to mass conflict and displacement: A systematic review and meta-analysis. JAMA - J Am Med Assoc. 2009;302:537–49.

7. Schick M, Zumwald A, Knopfli B, Nickerson A, Bryant RA, Schnyder U, et al. Challenging future, challenging past: The relationship of social integration and psychological impairment in traumatized refugees. Eur J Psychotraumatol. 2016;7:1–9.

8. UNHCR. Global Trends Forced Displacement in 2020. 2021 [cited 2021 Sep 27]. Available from: https://www.unhcr.org/flagship-reports/globaltrends/

9. Fazel M, Reed R V, Panter-Brick C, Stein A. Mental health of displaced and refugee children resettled in high-income countries: Risk and protective factors. Lancet. 2012;379:266–82. Available from: mina.fazel@psych.ox.ac.uk



For peer review only - http://bmjopen.bmj.com/site/about/guidelines.xhtml

10. WHO. Building Back Better: Sustainable Mental Health Car after Emergencies. World Heal. Organ. 2013.

11. Fuhr DC, Acarturk C, McGrath M, Ilkkursun Z, Sondorp E, Sijbrandij M, et al. Treatment gap and mental health service use among Syrian refugees in Sultanbeyli, Istanbul: a cross-sectional survey. Epidemiol Psychiatr Sci. 2020;29:e70.

12. Lebano A, Hamed S, Bradby H, Gil-Salmerón A, Durá-Ferrandis E, Garcés-Ferrer J, et al. Migrants' and refugees' health status and healthcare in Europe: A scoping literature review. BMC Public Health. BMC Public Health; 2020;20.

13. Satinsky E, Fuhr DC, Woodward A, Sondorp E, Roberts B. Mental health care utilisation and access among refugees and asylum seekers in Europe: A systematic review. Health Policy (New York). Elsevier Ireland Ltd; 2019;123:851–63. Available from: https://doi.org/10.1016/j.healthpol.2019.02.007

14. Kiselev N, Pfaltz M, Haas F, Schick M, Kappen M, Sijbrandij M, et al. Structural and socio-cultural barriers to accessing mental healthcare among Syrian refugees and asylum seekers in Switzerland. Eur J Psychotraumatol. Taylor & Francis; 2020;11. Available from: https://doi.org/10.1080/20008198.2020.1717825

15. Due C, Green E, Ziersch A. Psychological trauma and access to primary healthcare for people from refugee and asylum-seeker backgrounds: A mixed methods systematic review. Int J Ment Health Syst. BioMed Central; 2020;14:1–18. Available from: https://doi.org/10.1186/s13033-020-00404-4

16. Sijbrandij M, Acarturk C, Bird M, Bryant RA, Burchert S, Carswell K, et al. Strengthening mental health care systems for Syrian refugees in Europe and the Middle East: integrating scalable psychological interventions in eight countries. Eur J Psychotraumatol. Taylor & Francis; 2017;8:1388102. Available from: https://www.tandfonline.com/doi/full/10.1080/20008198.2017.1388102

For peer review only


For peer review only - http://bmjopen.bmj.com/site/about/guidelines.xhtml

17. WHO. Scalable psychological interventions for people in communities affected by adversity. A new area of mental health and psychosocial work at WHO. 2017.

18. WHO. Mental Health Gap Action Programme: scaling up care for mental, neurological and substance use disorders. mhGAP Ment. Heal. Gap Action Program. 2008. Available from: http://www.who.int/mental_health/evidence/mhGAP/en/

19. Singla DR, Kohrt BA, Murray LK, Anand A, Chorpita BF, Patel V. Psychological treatment for the world: Lessons from low- and middle-income countries. Annu Rev Clin Psychol. 2017;49:149–81.

20. Dawson KS, Bryant RA, Harper M, Kuowei Tay A, Rahman A, Schafer A, et al. Problem Management Plus (PM+): a WHO transdiagnostic psychological intervention for common mental health problems. World Psychiatry. 2015;14:354–7.

21. Rahman A, Hamdani SU, Awan NR, Bryant RA, Dawson KS, Khan MF, et al. Effect of a multicomponent behavioral intervention in adults ipairment by psychological distress in a conflict-affected area of Pakistan. A randomized controlled trial. JAMA. 2016;316:2609–17.

22. Rahman A, Khan MN, Hamdani SU, Chiumento A, Akhtar P, Nazir H, et al. Effectiveness of a brief group psychological intervention for women in a post-conflict setting in Pakistan: a single-blind, cluster, randomised controlled trial. Lancet. Elsevier Ltd; 2019; Available from: https://linkinghub.elsevier.com/retrieve/pii/S0140673618323432

23. Bryant RA, Schafer A, Dawson KS, Anjuri D, Mulili C, Ndogoni L, et al. Effectiveness of a brief behavioural intervention on psychological distress among women with a history of gender-based violence in urban Kenya: A randomised clinical trial. PLoS Med. 2017;14:e1002371.

24. Jordans MJD, Kohrt BA, Sangraula M, Turner EL, Wang X, Shrestha P, et al. Effectiveness of Group Problem Management Plus, a brief psychological intervention for adults affected by humanitarian disasters in Nepal: A cluster randomized controlled trial. PLoS Med. 2021;18:1–22.

25. Hamdani SU, Huma ZE, Rahman A, Wang D, Chen T, Van Ommeren M, et al. Cost-

For peer review only



For peer review only - http://bmjopen.bmj.com/site/about/guidelines.xhtml

effectiveness of WHO Problem Management plus for adults with mood and anxiety disorders in a post-conflict area of Pakistan: Randomised controlled trial. Br J Psychiatry. 2020;217:623–9.

26. Carswell K, Harper-Shehadeh M, Watts S, van't Hof E, Abi Ramia J, Heim E, et al. Step-by-Step: a new WHO digital mental health intervention for depression. mHealth. 2018;4:34–34.

27. Harper Shehadeh MJ, Abi Ramia J, Cuijpers P, El Chammay R, Heim E, Kheir W, et al. Step-by-Step, an E-Mental Health Intervention for Depression: A Mixed Methods Pilot Study From Lebanon. Front Psychiatry. 2020;10:1–11.

28. Heim E, Ramia JA, Hana RA, Burchert S, Carswell K, Cornelisz I, et al. Step-by-step: Feasibility randomised controlled trial of a mobile-based intervention for depression among populations affected by adversity in Lebanon. Internet Interv. Elsevier B.V.; 2021;24:100380. Available from: https://doi.org/10.1016/j.invent.2021.100380

29. de Graaff AM, Cuijpers P, McDaid D, Park A, Woodward A, Bryant RA, et al. Peer-provided Problem Management Plus (PM+) for adult Syrian refugees; a pilot randomized controlled trial on effectiveness and cost-effectiveness. Epidemiol Psychiatr Sci. 2020;29:1–24.

30. Akhtar A, Giardinelli L, Bawaneh A, Awwad M, Naser H, Whitney C, et al. Group problem management plus (gPM+) in the treatment of common mental disorders in Syrian refugees in a Jordanian camp: Study protocol for a randomized controlled trial. BMC Public Health. BMC Public Health; 2020;20:1–8.

31. Acarturk C, Uygun E, Ilkkursun Z, Yurtbakan T, Adam-Troian J, Senay I, et al. Group Problem Management Plus (PM+) to decrease psychological distress among Syrian refugees in Turkey: A pilot randomised controlled trial. BMC Psychiatry. 2022;22:8.

32. Spaaij J, Kiselev N, Berger C, Bryant R, Cuijpers P, de Graaff A, et al. Feasibility and acceptability of Problem Management Plus (PM+) among Syrian refugees in Switzerland: a pilot randomized controlled trial.

33. Akhtar A, Giardinelli L, Bawaneh A, Awwad M, Al-Hayek H, Whitney C, et al. Feasibility trial

For peer review only


For peer review only - http://bmjopen.bmj.com/site/about/guidelines.xhtml

of a scalable transdiagnostic group psychological intervention for Syrians residing in a refugee camp. European Journal of Psychotraumatology; 2021;12:1932295. Available from: https://doi.org/10.1080/20008198.2021.1932295

34. de Graaff A, Cuijpers P, Acarturk C, Bryant R, Burchert S, Fuhr D, et al. Effectiveness of a peer-refugee delivered psychological intervention to reduce psychological distress among adult Syrian refugees in the Netherlands: study protocol. European Journal of Psychotraumatology; 2020;11:1694347.

35. Uygun E, Ilkkursun Z, Sijbrandij M, Aker AT, Bryant R, Cuijpers P, et al. Protocol for a randomized controlled trial: Peer-to-peer Group Problem Management plus (PM+) for adult Syrian refugees in Turkey. Trials. Trials; 2020;21:1–9.

36. Cuijpers P, Quero S, Papola D, Cristea IA, Karyotaki E. Care-as-usual control groups across different settings in randomized trials on psychotherapy for adult depression: a meta-analysis. Psychol Med. 2021;51:634–44.

37. Cratsley K, Brooks MA, Mackey TK. Refugee Mental Health, Global Health Policy, and the Syrian Crisis. Front Public Heal. 2021;9:1–20.

38. Riley RD, Debray TPA, Fisher D, Hattle M, Marlin N, Hoogland J, et al. Individual participant data meta-analysis to examine interactions between treatment effect and participant-level covariates: Statistical recommendations for conduct and planning. Stat Med. 2020;39:2115–37.

39. Berlin JA, Santanna J, Schmid CH, Szczech LA, Feldman HI. Individual patient- versus group-level data meta-regressions for the investigation of treatment effect modifiers: Ecological bias rears its ugly head. Stat Med. 2002;21:371–87.

40. Fisher DJ, Carpenter JR, Morris TP, Freeman SC, Tierney JF. Meta-analytical methods to identify who benefits most from treatments: Daft, deluded, or deft approach? BMJ. 2017;356:1–6.

41. Stewart L, Parmar M. Meta-analysis pof the literature or of individual patient data: is there a difference? Lancet. 1993;341:418–22.



For peer review only - http://bmjopen.bmj.com/site/about/guidelines.xhtml

42. Moher D, Shamseer L, Clarke M, Ghersi D, Liberati A, Petticrew M, et al. Preferred reporting items for systematic review and meta-analysis protocols (PRISMA-P) 2015 statement. Syst Rev. 2015;4:1.

43. Kessler RC, Andrews G, Colpe LJ, Hiripi E, Mroczek DK, Normand S-LT, et al. Short screening scales to monitor population prevalences and trends in non-specific psychological distress. Psychol Med. 2002;32:959–76. Available from: http://www.journals.cambridge.org/abstract_S0033291702006074

44. WHO. Measuring Health and Disability: Manual for WHO Disability Assessment Schedule WHODAS 2.0. Üstun TB, Kostansjek N, Chatterji S, Rehm J, editors. Geneva, Switzerland: WHO; 2010.

45. Sulaiman-Hill CM, Thompson SC. Selecting instruments for assessing psychological wellbeing in Afghan and Kurdish refugee groups. BMC Res Notes. 2010;3:237.

46. Akhtar A, Cuijpers P, Morina N, Sijbrandij M, Bryant R. Exploratory and confirmatory factor analysis of the 12-item Arabic World Health Organization Disability Assessment Schedule (WHODAS 2.0) as a screening tool for Syrian refugees. BJPsych Open. 2021;7:1–7.

47. Slewa-Younan S, Mond JM, Bussion E, Melkonian M, Mohammad Y, Dover H, et al. Psychological trauma and help seeking behaviour amongst resettled Iraqi refugees in attending English tuition classes in Australia. Int J Ment Health Syst. 2015;9:1–6.

48. WHO. Problem Management Plus (PM+). Individual psychological help for adults impaired by distress in communities exposed to adversity. Generic fi. Geneva: WHO; 2016. Available from: http://www.who.int/entity/mental_health/emergencies/problem_management_plus/en/index.html

49. Bernal G, Sáez-Santiago E. Culturally centered psychosocial interventions. J Community Psychol. 2006;34:121–32.

50. Burchert S, Alkneme MS, Bird M, Carswell K, Cuijpers P, Hansen P, et al. User-centered app adaptation of a low-intensity e-mental health intervention for Syrian refugees. Front Psychiatry.



For peer review only - http://bmjopen.bmj.com/site/about/guidelines.xhtml

2019;10.

51. Akhtar A, Engels MH, Bawaneh A, Bird M, Bryant R, Cuijpers P, et al. Cultural Adaptation of a Low-Intensity Group Psychological Intervention for Syrian Refugees. Intervention. 2021;19:48–57.

52. Derogatis LR, Lipman RS, Rickels K, Uhlenhuth EH, Covi L. The Hopkins Symptom Checklist (HSCL): A self-report inventory. Behav Sci. 1974;19:1–15.

53. Mahfoud Z, Kobeissi L, Peters TJ, Araya R, Ghantous Z, Khoury B. The Arabic validation of the Hopkins Symptoms Checklist-25 against MINI in a disadvantaged suburb of Beirut, Lebanon. Int J Educ Psychol Assess. 2013;13:17–33.

54. Selmo P, Koch T, Brand J, Wagner B, Knaevelsrud C. Psychometric Properties of the Online Arabic Versions of BDI-II, HSCL-25, and PDS. Eur J Psychol Assess. 2016;35:46–54. Available from: http://econtent.hogrefe.com/doi/10.1027/1015-5759/a000367

55. Blevins CA, Weathers FW, Davis MT, Witte TK, Domino JL. The Posttraumatic Stress Disorder Checklist for DSM-5 (PCL-5): Development and initial psychometric evaluation. J Trauma Stress. 2015;28:489–98.

56. Ashworth M, Shepher M, Christey J, Matthews V, Wright K, Parmentier H, et al. A client-generated psychometric instrument: the development of “PSYCHLOPS.” Couns Psychother Res. 2004;4:27–32.

57. Ibrahim H, Ertl V, Catani C, Ismail AA, Neuner F. The validity of Posttraumatic Stress Disorder Checklist for DSM-5 (PCL-5) as screening instrument with Kurdish and Arab displaced populations living in the Kurdistan region of Iraq. BMC Psychiatry. BMC Psychiatry; 2018;18:259.

58. Acarturk C, Konuk E, Cetinkaya M, Senay I, Sijbrandij M, Gulen B, et al. The efficacy of eye movement desensitization and reprocessing for post-traumatic stress disorder and depression among Syrian refugees: Results of a randomized controlled trial. Psychol Med. 2016;46:2583–93.


For peer review only - http://bmjopen.bmj.com/site/about/guidelines.xhtml

59. Beecham J, Knapp M. Costing psychiatric interventions. In: Thornicroft G, Brewin C, Wing J, editors. Meas Ment Heal Needs. 1st ed. London: Gaskell; 1992. p. 163–83.

60. The GRADE Working Group. GRADE handbook for grading quality of evidence and strength of recommendations. Schünemann H, Brożek J, Guyatt G, Oxman A, editors. 2013. Available from: guidelinedevelopment.org/handbook

61. Balshem H, Helfand M, Schünemann HJ, Oxman AD, Kunz R, Brozek J, et al. GRADE guidelines: 3. Rating the quality of evidence. J Clin Epidemiol. 2011;64:401–6.

62. Evers S, Goossens M, De Vet H, Van Tulder M, Ament A. Criteria list for assessment of methodological quality of economic evaluations: Consensus on Health Economic Criteria. Int J Technol Assess Health Care. 2005;21:240–5.

63. R Core Team. R: A language and environment for statistical computing. Vienna, Austria: R Foundation for Statistical Computing; 2021.

64. Viechtbauer W. Conducting meta-analyses in R with the metafor. J Stat Softw. 2010;36:1–48.

65. Bates D, Mächler M, Bolker BM, Walker SC. Fitting linear mixed-effects models using lme4. J Stat Softw. 2015;67.

66. StataCorp. Stata Statistical Software. College Station, TX: StataCorp LLC; 2021.

67. Burke DL, Ensor J, Riley RD. Meta-analysis using individual participant data: one-stage and two-stage approaches, and why they may differ. Stat Med. 2017;36:855–75.

68. Bell ML, Fiero M, Horton NJ, Hsu CH. Handling missing data in RCTs; A review of the top medical journals. BMC Med Res Methodol. 2014;14:1–8.

69. White I, Horton N, Carpenter J, Pocock S. Strategy for intention to treat analysis in randomised trials with missing outcome data. BMJ. 2011;342:d40. Available from: https://www.ncbi.nlm.nih.gov/pmc/articles/PMC3230114/

70. Riley RD, Lambert PC, Abo-Zaid G. Meta-analysis of individual participant data: Rationale, conduct, and reporting. BMJ. 2010;340:521–5.


For peer review only


For peer review only - http://bmjopen.bmj.com/site/about/guidelines.xhtml

71. Higgins JPT, Thompson SG, Deeks JJ, Altman DG. Measuring inconsistency in meta-analyses. Br Med J. 2003;327:557–60.

72. Orsini N, Bottai M, Higgins J, Buchan I. Heterogi: Stata module to quantify heterogeneity in a meta-analysis. Boston: Boston College Department of Economics; 2005. p. S449201.

73. Egger M, Smith GD, Schneider M, Minder C. Bias in meta-analysis detected by a simple, graphical test. BMJ. 1997;315:629–34.

74. Duval S, Tweedie R. Trim and Fill: A Simple Funnel-Plot-Based Method. Biometrics. 2000;56:455–63.

75. Drescher A, Kiselev N, Akhtar A, Acarturk C, Bryant RA, Ilkkursun Z, et al. Problems after flight: understanding and comparing Syrians' perspectives in the Middle East and Europe. BMC Public Health. BMC Public Health; 2021;21:1–12.

76. Winhusen T, Winstanley EL, Somoza E, Brigham G. The potential impact of recruitment method on sample characteristics and treatment outcomes in a psychosocial trial for women with co-occurring substance use disorder and PTSD. Drug Alcohol Depend. Elsevier Ireland Ltd; 2012;120:225–8. Available from: http://dx.doi.org/10.1016/j.drugalcdep.2011.06.014

77. Fried EI, van Borkulo CD, Epskamp S, Schoevers RA, Tuerlinckx F, Borsboom D. Measuring depression over time...or not? lack of unidimensionality and longitudinal measurement invariance in four common rating scales of depression. Psychol Assess. 2016;28:1354–67.

78. Fokkema M, Smits N, Kelderman H, Cuijpers P. Response shifts in mental health interventions: an illustration of longitudinal measurement invariance. Psychol Assess. 2013;25:520–31.

For peer review only



For peer review only - http://bmjopen.bmj.com/site/about/guidelines.xhtml

Table 1.

*Trial Descriptions*

| Study site | Trial | Intervention | Setting | Inclusion criteria | Exclusion criteria | Pre-registration trial | Trial dates [a] |
|---|---|---|---|---|---|---|---|
| Netherlands | Pilot | PM+ | Community | STRENGTHS criteria [b] | STRENGTHS criteria [c] | NTR: NL6665 | April 2018-March 2019 |
| | RCT | PM+ | Community | STRENGTHS criteria [b] | STRENGTHS criteria [c]; *Current receipt of specialized psychological treatment* | NTR: NL7552 | Feb 2019-*ongoing* |
| Switzerland | Pilot | PM+ | Community | STRENGTHS criteria [b] | STRENGTHS criteria [c]; *Being under guardianship; Inability to follow study procedures* | ClinicalTrials.gov: NCT03830008 | Dec 2018-March 2020 |
| | RCT | PM+ | Community | STRENGTHS criteria [b] + *Arabic-speaking refugees 18+ yrs* | STRENGTHS criteria [c]; *Being under guardianship; Inability to follow study procedures* | ClinicalTrials.gov: NCT04574466 | Feb 2020-*ongoing* |
| Turkey | Pilot | gPM+ | Community | STRENGTHS criteria [b] | STRENGTHS criteria [c] | ClinicalTrials.gov: NCT03567083 | Sept 2018-Jan 2019 |
| | RCT | gPM+ | Community | STRENGTHS criteria [b] | STRENGTHS criteria [c] | ClinicalTrials.gov: NCT03960892 | Aug 2019-Nov 2021 |


For peer review only - http://bmjopen.bmj.com/site/about/guidelines.xhtml

| | | | | | | | |
|---|---|---|---|---|---|---|---|
| Jordan | Pilot | gPM+ | Refugee camp | STRENGTHS criteria [b] + *Having a child or dependent aged 10-16 yrs* | STRENGTHS criteria [c] | ANZCTR: ACTRN12619000168156 | Jan-April 2019 |
| | RCT | gPM+ | Refugee camp | STRENGTHS criteria [b] + *Having a child or dependent aged 10-16 yrs* | STRENGTHS criteria [c] | ANZCTR: ACTRN12619001386123 | Aug 2019-July 2020 |
| Germany | Pilot | SbS | Digital | STRENGTHS criteria [b] | *Signs of imminent suicide risk* | DRKS: DRKS00017838 | Dec 2019-Oct 2020 |
| | RCT | SbS | Digital | STRENGTHS criteria [b] | *Signs of imminent suicide risk* | DRKS: DRKS00022143 | Aug 2020-o*ngoing* |
| Sweden | RCT | SbS | Digital | STRENGTHS criteria [b] | *Signs of imminent suicide risk* | DRKS: DRKS00022144 | Aug 2020-*ongoing* |
| Egypt | RCT | SbS | Digital | STRENGTHS criteria [b] | *Signs of imminent suicide risk* | DRKS: DRKS00023505 | March 2021-o*ngoing* |

[a] Recruitment to 12-month follow-up; [b] STRENGTHS inclusion criteria = Arabic-speaking Syrian refugees 18+ yrs + K10 >15 + WHODAS 2.0 >16; [c] STRENGTHS exclusion criteria = Signs of imminent suicide risk, Acute medical conditions, Expressed acute needs or protection risks, and/or Indications of severe mental disorders; Pilot = pilot randomized controlled trial (RCT); RCT = fully-powered RCT; PM+ = Problem Management Plus; gPM+ = group PM+; SbS = Step-by-Step; K10 = Kessler Psychological Distress Scale; WHODAS 2.0 = WHO Disability Assessment Schedule 2.0; NTR = Netherlands Trial Register; ANZCTR = Australian New Zealand Clinical Trials Registry; DRKS: German Clinical Trials Register

For peer review only



For peer review only - http://bmjopen.bmj.com/site/about/guidelines.xhtml

## PRISMA-P (Preferred Reporting Items for Systematic review and Meta-Analysis Protocols) 2015 checklist: recommended items to address in a systematic review protocol*

| Section and topic | Item No | Checklist item | Page |
|---|---|---|---|
| **ADMINISTRATIVE INFORMATION** | | | |
| Title: | | | |
| Identification | 1a | Identify the report as a protocol of a systematic review | 1 |
| Update | 1b | If the protocol is for an update of a previous systematic review, identify as such | N/A |
| Registration | 2 | If registered, provide the name of the registry (such as PROSPERO) and registration number | OSF DOI 10.17605/OSF.IO/WUHGF |
| Authors: | | | |
| Contact | 3a | Provide name, institutional affiliation, e-mail address of all protocol authors; provide physical mailing address of corresponding author | 1-2 |
| Contributions | 3b | Describe contributions of protocol authors and identify the guarantor of the review | 14 |
| Amendments | 4 | If the protocol represents an amendment of a previously completed or published protocol, identify as such and list changes; otherwise, state plan for documenting important protocol amendments | N/A |
| Support: | | | |
| Sources | 5a | Indicate sources of financial or other support for the review | 14 |
| Sponsor | 5b | Provide name for the review funder and/or sponsor | 14 |
| Role of sponsor or funder | 5c | Describe roles of funder(s), sponsor(s), and/or institution(s), if any, in developing the protocol | 14 |
| **INTRODUCTION** | | | |
| Rationale | 6 | Describe the rationale for the review in the context of what is already known | 5-7 |
| Objectives | 7 | Provide an explicit statement of the question(s) the review will address with reference to participants, interventions, comparators, and outcomes (PICO) | 7 |
| **METHODS** | | | |
| Eligibility criteria | 8 | Specify the study characteristics (such as PICO, study design, setting, time frame) and report characteristics (such as years considered, language, publication status) to be used as criteria for eligibility for the review | 7-8 |
| Information | 9 | Describe all intended information sources (such as electronic databases, contact with study authors, trial registers or other | 7 |

For peer review only

| | | | |
|---|---|---|---|
| sources | | grey literature sources) with planned dates of coverage | |
| Search strategy | 10 | Present draft of search strategy to be used for at least one electronic database, including planned limits, such that it could be repeated | N/A |
| Study records: | | | |
| Data management | 11a | Describe the mechanism(s) that will be used to manage records and data throughout the review | 10 |
| Selection process | 11b | State the process that will be used for selecting studies (such as two independent reviewers) through each phase of the review (that is, screening, eligibility and inclusion in meta-analysis) | N/A |
| Data collection process | 11c | Describe planned method of extracting data from reports (such as piloting forms, done independently, in duplicate), any processes for obtaining and confirming data from investigators | 10 |
| Data items | 12 | List and define all variables for which data will be sought (such as PICO items, funding sources), any pre-planned data assumptions and simplifications | 10-12 |
| Outcomes and prioritization | 13 | List and define all outcomes for which data will be sought, including prioritization of main and additional outcomes, with rationale | 10 |
| Risk of bias in individual studies | 14 | Describe anticipated methods for assessing risk of bias of individual studies, including whether this will be done at the outcome or study level, or both; state how this information will be used in data synthesis | 11 |
| Data synthesis | 15a | Describe criteria under which study data will be quantitatively synthesised | 11-12 |
| | 15b | If data are appropriate for quantitative synthesis, describe planned summary measures, methods of handling data and methods of combining data from studies, including any planned exploration of consistency (such as $I^2$, Kendall's $\tau$) | 11-12 |
| | 15c | Describe any proposed additional analyses (such as sensitivity or subgroup analyses, meta-regression) | 11 |
| | 15d | If quantitative synthesis is not appropriate, describe the type of summary planned | N/A |
| Meta-bias(es) | 16 | Specify any planned assessment of meta-bias(es) (such as publication bias across studies, selective reporting within studies) | 12-13 |
| Confidence in cumulative evidence | 17 | Describe how the strength of the body of evidence will be assessed (such as GRADE) | 11 |

*** It is strongly recommended that this checklist be read in conjunction with the PRISMA-P Explanation and Elaboration (cite when available) for important clarification on the items. Amendments to a review protocol should be tracked and dated. The copyright for PRISMA-P (including checklist) is held by the PRISMA-P Group and is distributed under a Creative Commons Attribution Licence 4.0.**

*From: Shamseer L, Moher D, Clarke M, Ghersi D, Liberati A, Petticrew M, Shekelle P, Stewart L, PRISMA-P Group. Preferred reporting items for systematic review and meta-analysis protocols (PRISMA-P) 2015: elaboration and explanation. BMJ. 2015 Jan 2;349(jan02 1):g7647.*

For peer review only

For peer review only
